# Antileishmanial and Antitoxoplasmal Activities of 1,4-Dihydropyridines

**DOI:** 10.1021/acsomega.5c04551

**Published:** 2025-07-10

**Authors:** Thaís A. S. Oliveira, Yan R. Robles, Ibrahim S. Al Nasr, Waleed S. Koko, Tariq A. Khan, Ismail Daoud, Seyfeddine Rahali, Noureddine Amdouni, Ridha B. Said, Antônio E. M. Crotti

**Affiliations:** † Department of Chemistry, Faculty of Philosophy, Sciences, and Letters at Ribeirão Preto, 124588 University of São Paulo, Ribeirão Preto 14900-001, São Paulo, Brazil; ‡ Department of Biology, College of Science, 158007 Qassim University, Qassim 51452, Saudi Arabia; § Department of Basic Health Sciences, College of Applied Medical Sciences, Qassim University, Qassim 51452, Saudi Arabia; ∥ Department of Matter Sciences, University Mohamed Khider, BP 145 RP, Biskra 07000, Algeria; ⊥ Laboratory of Natural and Bio-Active Substances, Faculty of Science, Tlemcen University, Tlemcen 13000 P.O. Box 119, Algeria; # Department of Chemistry, College of Science, Qassim University, Qassim 51452, Saudi Arabia; ¶ Laboratoire de Caracte’risations, Applications et Mode’lisations des Mate’riaux, Faculte’ des Sciences de Tunis, Universite’ Tunis El Manar, Tunis 1068, Tunisia

## Abstract

We have synthesized 24 1,4-dihydropyridine compounds (1,4-DHPs) with different substituents at the aromatic ring by microwave-assisted one-pot Hantzsch multicomponent reaction and evaluated their in vitro activities against *Toxoplasma gondii* and *Leishmania major*. We have found that compound **9** ((±)-ethyl 2,7,7-trimethyl-4-(2-nitrophenyl)-5-oxo-1,4,5,6,7,8-hexahydroquinoline-3-carboxylate) is active against *L. major* amastigotes (IC_50_ = 10.6 μM), but it is poorly selective for *L. major* over Vero cells (SI = 1.13) and macrophages (SI = 0.42). Among the evaluated 1,4-DHPs, compound **4** ((±)-ethyl 4-(4-(benzyloxy)­phenyl)-2,7,7-trimethyl-5-oxo-1,4,5,6,7,8-hexahydroquinoline-3-carboxylate) is the most active against *T. gondii*, providing the lowest IC_50_ (3.1 μM) and the highest selectivity for this parasite (SI = 5.57) over Vero cells. Docking studies revealed that compound **4** has a high affinity for the *T. gondii* target (PDB ID: 4JBV). Furthermore, ADME-T predictions indicated that compound **4** meets the drug-likeness criteria without violating any Lipinski, Veger, or Egan’s rules.

## Graphical abstract

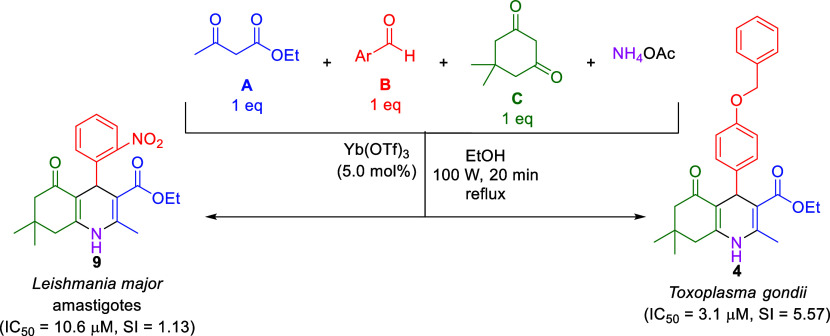

## 1 Introduction

Leishmaniasis, a neglected tropical disease (NTD) that results in about 30,000 deaths annually,^1,2^ is caused by protozoa of the genus *Leishmania* and is clinically subdivided into cutaneous leishmaniasis (CL), mucocutaneous leishmaniasis (MCL), and visceral leishmaniasis (VL). *Leishmania major* is one of the species that trigger CL, which affects up to 1 million individuals every year and can result in severe and disfiguring skin lesions, thereby posing the risk of stigmatization, especially among young patients.^3–5^ Current treatment options for CL patients include antimonials, miltefosine, amphotericin B, and pentamidine.^6^ However, these drugs are toxic, not to mention that drug-resistant parasite forms have emerged, which calls for new potent antileishmanial drugs.^7^

Toxoplasmosis, a zoonotic infectious disease caused by the apicomplexan protozoan *Toxoplasma gondii*, infects about one-third of the world’s human population.^8^ Immunocompromised individuals are particularly vulnerable to serious complications following infection by *T. gondii*.^9^ Currently, the available antiparasitic drugs for toxoplasmosis include sulfonamides (e.g., sulfadiazine), DHFR inhibitors (pyrimethamine, trimethoprim), and the naphthoquinone atovaquone.^10^ However, adverse effects such as nephrotoxicity,^11^ alongside the emergence of drug-resistant clinical isolates, highlight the urgent need for novel and effective therapeutic agents for the treatment and management of toxoplasmosis.^12^

1,4-DHPs are known for their ability to block calcium channels.^13^ Nevertheless, over the past decade, several studies on their antiparasitic activity have been published. For example, the antileishmanial activity of 1,4-DHPs against *Leishmania amazonensis*, the main *Leishmania* species responsible for CL in the Americas, has been extensively reported.^1,14^ Some studies on 1,4-DHPs have also demonstrated the antiparasitic effects against *T. gondii*.^3^

As part of our interest in exploring the antiparasitic potential of natural^15^ and synthetic products,^16,17^ and based on previous reports on the antiparasitic activities of 1,4-dihydropyridines (1,4-DHPs),^1,14,18^ we have assessed the antiparasitic activities of 24 synthetic 1,4-DHPs against *L. major* and *T. gondii*.

## 2 Materials and Methods

### 2.1 Synthesis of Compounds **1**–**24**

The 1,4-DHPs were synthesized according to the multicomponent one-pot methodology described in the literature.^1,19^ In the general procedure, 2.0 mmol of dimedone (Aldrich), 2.0 mmol of ethyl acetoacetate (Aldrich), and 0.06 g (5.0 mol %) of ytterbium triflate (Aldrich), as reaction catalyst, were diluted in ethanol (5.0 mL). Subsequently, 2.0 mmol of benzaldehyde (Aldrich) and 2.0 mmol of ammonium acetate (Scientific Exodus) were added. All the reagents were added at room temperature. The reaction mixture was taken to the microwave reactor CEM FocusedMicrowave Synthesis System, model Discover (CEM Corp, Matthews, NC) set in the Power Time, where it was maintained for 30 min at a fixed power of 100 W. Compounds **1**–**24** were identified based on their NMR (^1^H, ^13^C, and DEPT 135) and mass spectra (see Supporting Information). NMR experiments were performed on a Bruker Avance DRX400 spectrometer (Karlsruhe, Germany, 400.13 MHz for ^1^H and 100.61 MHz for ^13^C). A direct 5 mm probe head (BBO) was used for ^13^C­{^1^H} NMR experiments and an inverse 5 mm probe head (BBI) was used for other experiments. All compounds were dissolved in CDCl_3_ using tetramethylsilane (TMS) as an internal reference to achieve concentrations in the range of 10–15 mg mL^–1^. All the experiments were performed at 300 K.

Mass spectra were recorded on a triple quadrupole MS equipment (QqQ) Xevo TQS (Waters, Milford, MA, USA) equipped with Z-spray operating in the positive ion mode and Acquiti-H class UPLC system. The sample was dissolved in methanol/water (9:1, v/v) at a concentration of 0.5 mg mL^–1^ and infused directly into the ESI source by using a Harvard Apparatus system (model 1746, Houston, MA, USA) at a flow rate of 5 μL min^–1^. The capillary voltage was 3.20 kV, and the gas flow was 700 L/h (0.15 V). The desolvation temperature was set at 250 °C.

Compounds **1–16** were isolated as mixtures of enantiomers. All the tested compounds displayed purity higher than 95%, as confirmed by HPLC analyses. To this end, compounds **1–24** were dissolved in an acetonitrile/water 4:1 (v/v) solution to achieve a concentration of 0.5 mg mL^–1^. These compounds were analyzed on a Shimadzu LC 6AD chromatograph fitted with a UV–vis SPD-20A operating at 270 nm and a DGU-20A5 degasser. A Phenomenex Luna C_18_ 100 Å (5 μm × 250 mm × 4.6 mm) column was used. The injection volume was 500 μL. A mixture of acetonitrile/water 4:1 (v/v) was used as the mobile phase at a flow rate of 1 mL min^–1^. The total time of each analysis was 1 h. All the HPLC chromatograms are given in the Supporting Information.

### 2.2 Anti-Toxoplasma Activity

Tachyzoites of the *T. gondii* RH strain were cultivated in Vero cells according to a published method.^20^ Complete RPMI 1640 medium (Invitrogen, USA) with 10% fetal bovine serum (FBS, Invitrogen, USA) was used to culture Vero cells in 96-well plates (5 × 10^3^ cells/well in 200 μL of medium), which were incubated at 37 °C and under 5% CO_2_ for 24 h. This was followed by washing with phosphate-buffered saline (PBS) to remove the medium. Then, RPMI 1640 medium with 2% FBS containing *T. gondii* tachyzoites (RH strain) at a ratio of 5 (parasite): 1 (Vero cells) was added. After incubation at 37 °C and under 5% CO_2_ for 5 h, the cells were washed with PBS and overlaid with medium containing one of the tested compounds or atovaquone (ATO) at 50, 25, 12.5, 6.25, 3.13, 1.65, 0.75, or 0.37 μg mL^–1^.

After incubation at 37 °C and under 5% CO_2_ for 72 h, the cells were stained with 1% toluidine before being examined under an inverted photomicroscope (MCD-400, Leica, Japan) to determine the *T. gondii* infection index (number of infected cells from 200 tested cells). The experiment was performed in triplicate.^4^

### 2.3 *Leishmania major* Promastigotes and Amastigotes

*L. major* promastigotes were isolated and maintained according to a described method.^21^ BALB/c mice were injected at the hind footpads with 1 × 10^6^ *L. major* metacyclic promastigotes. *L. major* amastigotes were collected from the infected mice. After 8 weeks had elapsed since inoculation, Schneider’s medium (Invitrogen, USA) containing antibiotics and 10% FBS was used to transform isolated amastigotes into promastigotes by incubation at 26 °C. Amastigote-derived promastigotes with less than five in vitro passages were only used for infection.

To assess the activity of the compounds against *L. major* promastigotes, 10^6^ promastigotes/mL were cultured in 96-well plates in Schneider’s medium with 10% FBS. Then, the compounds or amphotericin B (AmB) were added to obtain the final concentrations (50, 25, 12.5, 6.25, 3.13, 1.65, 0.75, or 0.37 μg mL^–1^). Negative control wells contained cultures with DMSO (1%) only. Plates were incubated at 26 °C for 72 h to evaluate the antiproliferative effect. The colorimetric MTT method was used to count viable promastigotes under a microplate absorbance spectrophotometer (xMark, Bio-Rad, USA) at 570 nm. The experiment was performed in triplicate.^4^

Female BALB/c mouse peritoneal macrophages (6–8 weeks old) were used to culture *L. major* amastigotes to assess the activity of the compounds against amastigotes. Briefly, 1 mL of 3% Brewer’s thioglycollate medium/mouse was injected into the peritoneal cavity. After 4 days, the abdominal skin was removed to expose the peritoneal wall. Then, 3 mL of RPMI 1640 medium was injected before cells were collected by aspiration, whereupon 96-well plates containing RPMI 1640 medium with 10% FBS were used to culture 5 × 10^4^ cells/well, which were incubated at 37 °C under 5% CO_2_ for 4 h. After washing with PBS, RPMI 1640 medium containing 5 × 10^5^ promastigotes were added to each well. Next, the cells were incubated at 37 °C under humidified 5% CO_2_ for 24 h to enhance infection and differentiation of amastigotes. Washing with PBS several times removed free promastigotes. Fresh complete RPMI 1640 medium containing one of the tested compounds or AmB at the desired final concentration (50, 25, 12.5, 6.25, 3.13, 1.65, 0.75, or 0.37 μg mL^–1^) was added and incubated at 37 °C under a humidified 5% CO_2_ atmosphere for 72 h. DMSO (1%), only in complete RPMI media, was used as the negative control. The percentage of infected macrophages was assessed microscopically after the medium was removed, followed by washing with PBS, fixation with methanol, and staining with Giemsa. The reading was performed in triplicate.^4^ Handling of the laboratory animals followed the instructions and rules of the Committee of Research Ethics, Deanship of Scientific Research, Qassim University, permission number 20–03–20.

### 2.4 Cytotoxicity Assay

The MTT colorimetric assay was conducted to assess the cytotoxicity of the compounds according to a method described previously.^22^ Briefly, Vero cells or macrophages were cultured in 96-well plates (5 × 10^3^ cells/well/200 μL) in RPMI 1640 medium with 10% FBS at 37 °C and under 5% CO_2_ for 24 h. Thereafter, PBS was used to wash the cells. Then, the compounds in RPMI complete medium with 10% FBS (at varying concentrations of 50, 25, 12.5, 6.25, 3.13, 1.65, 0.75, or 0.37 μg mL^–1^) were added to 96-well plates and incubated for 72 h. Cells treated with a medium containing only DMSO (1%) were used as the negative control. After washing, MTT (1 mg mL^–1^ in RPMI 1640 medium) was added to each well. The cells were incubated for 4 h, and the supernatant was removed. Next, 150 μL of DMSO was added, and a microplate absorbance spectrophotometer was applied for colorimetric analysis (λ = 540 nm). Cytotoxic effects were expressed by IC_50_ values (concentration that caused a 50% reduction of viable cells). The experiment was performed in triplicate.^23^

### 2.5 Molecular Docking

#### 2.5.1 Ligands and Target Preparation

The geometry of compound **4** was optimized by using the Density Functional Theory (DFT) method.^24–26^ The effect of the solvent (water) was considered by using CPCM.^27^ The exchange Becke three-parameter Lee–Yang–Parr correlation (B3LYP) level of theory^24^ in conjunction with dispersion correction D3,^26^ already implemented in Gaussian 16 ^28^ and the base 6–311G (d, p),^29^ were employed.

#### 2.5.2 Target Preparations

The crystal structure of calcium-dependent protein kinase-1 (PDB ID: 4JBV, resolution 1.95 Å) was obtained from the Protein Data Bank (PDB) (https://www.rcsb.org/). This *T. gondii* target (*Tg*CDPK1) was selected based on the recent literature.^30,31^

#### 2.5.3 Molecular Docking Protocol and Validation

Docking calculations were performed on the selected proteins by using the “MOE Docking Option” implemented in the MOE software (Molecular Operating Environment (MOE) 2014.09).^32^ Therefore, the software was used to calculate the s-score energy and to predict interactions between proteins and compounds by keeping the macromolecule rigid and the compound flexible. The crystal structures of the studied proteins were simplified by removing water molecules, ions, cofactors, and native ligands from the PDB structures. The molecular docking simulation protocol used in this study has been detailed.^23,31^ The molecular docking method is validated by redocking the crystallized ligands with their targets and calculating the RMSD (the RMSD of the resulting complex lies between 1 and 2 Å),^33^ which represents the accuracy and satisfaction of the docking method. The 2D Interaction diagrams between compound **4** and *T. gondii* target (4JBV). Furthermore, ADME-T predictions indicated that this compound meets the drug-likeness criteria without violating any Lipinski, V) were visualized by using the BIOVIA DS visualization package (Dassault Systèmes BIOVIA, Discovery Studio modeling environment, 2020).

#### 2.5.4 Drug-Likeness Prediction and ADME-T

The number of hydrogen bond acceptors (nHA), the number of hydrogen bond donors (nHD), TPSA, nROT, MW, and LogP were obtained from the SwissADME server (http://www.swissadme.ch/ accessed on 15 April 2024).^34^ Besides that, Absorption, Distribution, Metabolism, Excretion, and Toxicity (ADME-T) were predicted by using the pkCSM server (http://biosig.unimelb.edu.au/pkcsm/prediction accessed on 15 April 2024).^35^

## 3 Results

### 3.1 Synthesis of Compounds **1**–**24**

We synthesized compounds **1**–**24** by a microwave-assisted one-pot Hantzsch multicomponent reaction between an aromatic aldehyde, ethyl acetoacetate, and a β-dicarbonyl compound (i.e., dimedone for the asymmetric compounds **1**–**16**, and ethyl acetoacetate excess for the symmetric compounds **17**–**24**) as previously reported^19^ (Scheme 1). We employed ytterbium triflate (Yb­(OTf)_3_) as a catalyst^36^ and ammonium acetate as a nitrogen donor.^37^ We used ethanol as the solvent, and the reaction time was 30 min. We isolated the product from the reaction mixture by vacuum filtration and purified it by recrystallization, to achieve yields of 15–40%.

**Scheme 1:**
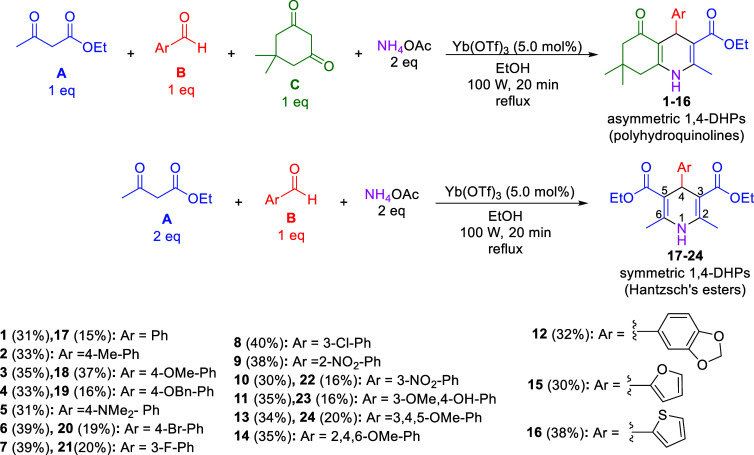
Synthesis of Compounds **1**–**24** Through a Microwave-Assisted One-Pot Hantzsch Multicomponent Reaction

### 3.2 Antileishmanial Activity of Compounds **1**–**24** against *L. major*

Table 1 depicts the antileishmanial activity of compounds **1**–**24** against *L. major* promastigotes and amastigotes.

**Table 1:** Effective Concentrations (EC_50_, in μM) of Compounds **1**–**24** against *Leishmania major* Promastigotes (pro**m**) and Amastigotes (am**a**) and *Toxoplasma gondii*; IC_50_ (in μM) of Compounds **1**–**24** against Macrophages (Macr**o**) and Vero (African Green Monkey Kidney Epithelial) Cells^a^

1,4-DHP	**EC**_**50**_			**IC**_**50**_			SI		
	*L. major*		*T. gondii*	Vero	macro	*L. major*			Vero/*T. gondii*
	prom	ama				Vero/prom	Vero/prom	macro/ama	
**1**	>61.3	>61.3	>61.3	>61.3	>61.3	n.d	n.d	n.d	n.d
**2**	>58.8	>58.8	>58.8	52.9 ± 9.8	47.8 ± 7.6	<0.90	<0.90	<0.81	<0.89
**3**	>56.3	>56.3	>56.3	49.8 ± 8.9	38.7 ± 6.8	<0.88	<0.88	<0.69	<1.13
**4**	>46.7	23.3 ± 3.4	3.1 ± 0.4	17.5 ± 3.0	25.1 ± 3.7	<0.37	<0.75	1.08	5.57
**5**	>54.4	>54.4	29.5 ± 4.1	49.4 ± 8.8	49.9 ± 7.4	<0.91	<0.91	0.92	1.67
**6**	>49.7	>49.7	23.4 ± 3.5	17.4 ± 2.9	23.4 ± 3.5	<0.35	<0.35	0.47	0.74
**7**	>58.2	>58.2	>58.2	>58.2	>58.2	n.d	n.d	n.d	n.d
**8**	>55.6	>55.6	>55.6	>55.6	>55.6	n.d	n.d	n.d	n.d
**9**	>58.2	10.6 ± 2.1	12.0 ± 1.9	4.5 ± 0.7	12.0 ± 1.9	<0.08	<0.42	1.13	0.37
**10**	>54.1	26.3 ± 3.7	16.4 ± 2.5	10.7 ± 2.1	24.5 ± 3.6	<0.20	<0.41	0.93	0.65
**11**	>54.0	>54.0	26.5 ± 3.6	45.1 ± 7.9	48.0 ± 8.1	<0.84	<0.89	0.89	1.70
**12**	47.5 ± 6.2	>60.6	30.3 ± 5.2	14.9 ± 2.6	42.5 ± 7.2	0.31	<0.25	0.70	0.49
**13**	>48.4	>48.4	>48.4	>48.4	>48.4	n.d	n.d	n.d	n.d
**14**	18.9 ± 2.9	21.0 ± 3.9	>48.4	22.8 ± 3.3	16.8 ± 3.1	0.89	1.09	0.8	<0.47
**15**	40.0 ± 6.4	>51.3	>51.3	>51.3	>51.3	1.28	n.d	n.d	n.d
**16**	>49.3	31.8 ± 5.4	8.1 ± 1.3	12.6 ± 2.1	29.9 ± 5.1	0.61	<0.40	0.94	1.55
**17**	>63.1	>63.1	49.8 ± 6.5	60.1 ± 10.6	>63.1	n.d	<0.95	n.d	1.21
**18**	>57.9	>57.9	>57.9	45.4 ± 7.8	>57.9	n.d	<0.78	n.d	<0.78
**19**	>47.8	>47.8	>47.8	33.5 ± 6.2	43.4 ± 7.5	0.70	<0.70	0.91	<0.70
**20**	>50.9	>50.9	>50.9	35.0 ± 5.9	44.8 ± 8.2	0.69	<0.69	0.88	<1.49
**21**	>59.9	>59.9	>59.9	>59.9	>59.9	n.d	n.d	n.d	n.d
**22**	46.5 ± 7.7	>55.6	17.4 ± 2.7	29.9 ± 4.4	46.5 ± 7.4	1.0	<0.54	0.84	1.72
**23**	>55.4	>55.4	29.8 ± 4.8	22.1 ± 3.2	40.5 ± 6.8	0.73	<0.40	0.73	0.74
**24**	42.7 ± 8.0	>49.6	>49.6	14.8 ± 2.6	34.1 ± 5.5	0.35	<0.30	0.69	<0.29
AmB	0.83 ± 0.2	0.47 ± 0.1	—	10.3 ± 1.2	8.1 ± 1.1	12.4	21.9	17.2	
ATO	—	—	0.07 ± 0.01	9.5 ± 1.5		—	—	—	135

a Amphotericin B (AmB) and atovaquone (ATO) were applied as positive controls. n.d.: not determined. Values are the means of at least three independent experiments ± SD and were obtained from concentration–response curves by calculating the percentage of treated cells compared to untreated controls after 72 h. ^b^The selectivity index (SI) (IC_50_/EC_50_) was calculated from the corresponding IC_50_ against Vero cells and EC_50_ against *L. major* promastigotes and *T. gondii*, or IC_50_ against macrophages. Linear regression equation was used for the IC_50_ and EC_50_ calculations using Microsoft Excel program version 16.

Only five compounds (**12**, **14**, **15**, **22**, and **24**) were active (IC_50_ < 50 μM) against *L. major* promastigotes. We obtained the lowest half-effective concentration (EC_50_) for compound **14** (EC_50_ = 18.9 μM). Similarly, only compounds **9** (EC_50_ = 10.6 μM), **4** (EC_50_ = 23.3 μM), **10** (EC_50_ = 26.3 μM), **14** (EC_50_ = 21.0 μM), and **16** (EC_50_ = 31.8 μM) had EC_50_ lower than 50 μM against *L. major* amastigotes. However, except for compound **4**, the IC_50_ of all the compounds against Vero cells resembled their EC_50_ against *L. major* promastigotes and amastigotes, which resulted in a low selectivity index (i.e., the ratio between EC_50_ and IC_50,_ which measures the window between cytotoxicity and biological activity) (SI < 1). Besides, compounds **4, 6**, **9**, **10**, and **14** were the most toxic to macrophages, having the lowest IC_50_ (25.1, 23.4, 12.0, 24.5, and 16.8 μM, respectively). Nevertheless, compound **9** was the most selective for *L. major* amastigotes (which grow within macrophages) over macrophages (SI = 1.13), but this selectivity was low as compared to the selectivity of amphotericin B (AmB).

### 3.3 Anti-*T. gondii* Activity of Compounds **1**–**24**

Table 1 lists the results of the anti-*T. gondii* activity of compounds **1**–**24**. Only 11 compounds had EC_50_ lower than 50 μM: **6** (23.4 μM), **9** (12.0 μM), **4** (3.1 μM), **5** (29.5 μM), **11** (26.5 μM), **10** (16.4 μM), **12** (30.3 μM), **16** (8.1 μM), **17** (49.8 μM), **23** (29.8 μM), and **22** (17.4 μM). On the other hand, Vero cells were slightly more sensitive to compounds **6**, **9**, **2**, **3**, **10**, **14**, **12**, **20**, **19**, and **23** than *T. gondii* (Table 1) as evidenced by their IC_50_. Compound **4** gave the highest SI (SI = 5.57). Meanwhile, most compounds were not selective for *T. gondii* over Vero cells. Atovaquone (ATO), the positive control, had EC_50_ of 0.07 μM and SI of 135.

### 3.4 Computational Approach

#### 3.4.1 Ligands and Target Preparations

Figure 1 illustrates the optimized geometry of compound **4**.

**Figure 1:**
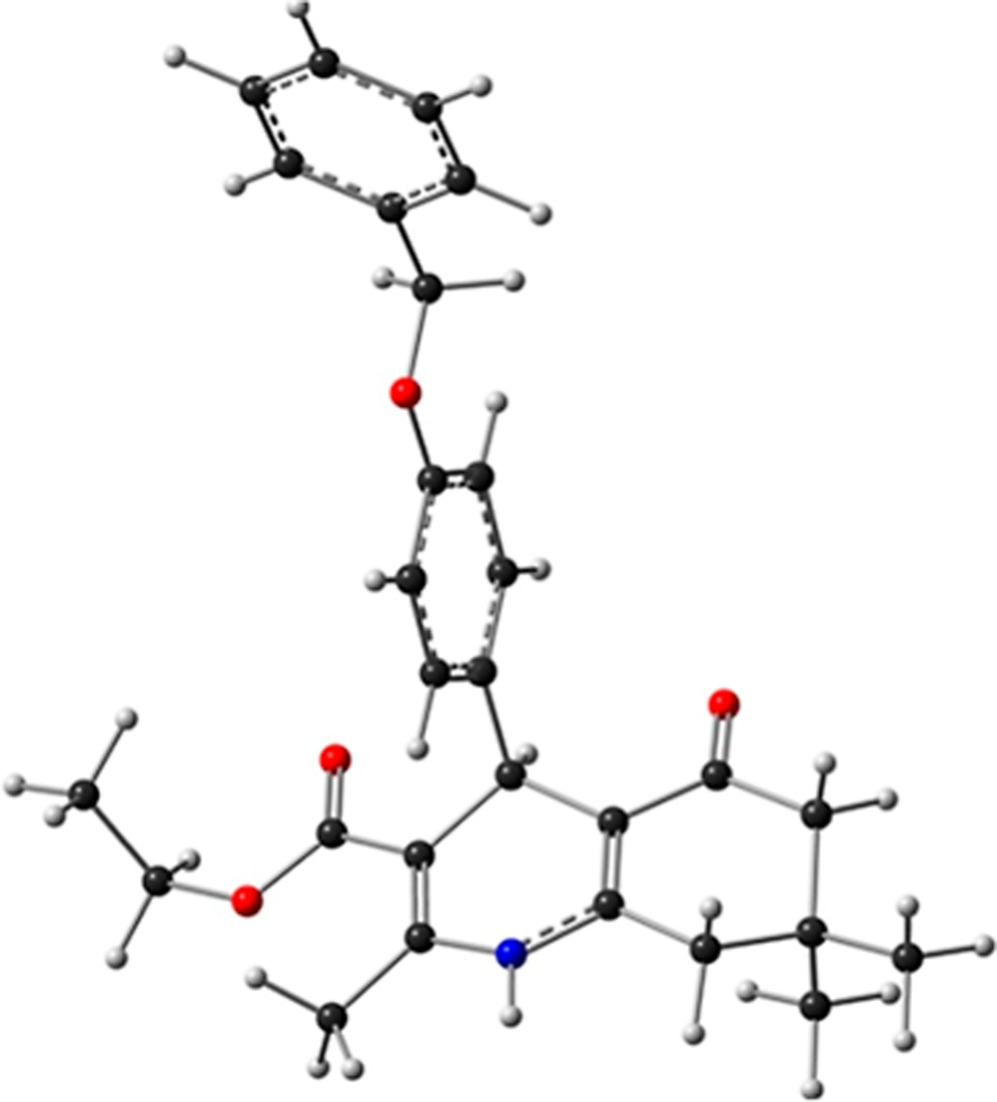
Optimized equilibrium structure of compound **4**.

#### 3.4.2 Score Energy and Best Poses Analysis

Table 2 regroups the docking simulation results obtained for compound **4** with the pocket of the *T. gondii* protein. The energy score for the complex of compound **4** with the *T. gondii* target (PDB ID: 4JBV) was −7.174 kcal/mol (Table 2).

**Table 2:** Docking Results for the Binding of Compound **4** and Co-crystallized Ligand (ATO) to the *Toxoplasma gondii* (PDB ID: 4JBV) Target^a^

compound	S-Score (kcal/mol)	RMSD (Å)	bonds between atoms of compounds and active site residues				
			atom of compound	involved receptor atoms	involved receptor	category	type
**4**	–7.174		H	O	LEU57	H-bond	conventional H-bond
				SD	MET112	other	Pi-sulfur
					LEU57	hydrophobic	Pi-alkyl
					VA65	hydrophobic	Pi-alkyl
		2.193			ALA78	hydrophobic	Pi-alkyl
					LEU181	hydrophobic	Pi-alkyl
					ILE194	hydrophobic	Pi-alkyl
					LYS80	hydrophobic	Pi-alkyl
					ILE194	hydrophobic	Pi-alkyl
cocrystallized ligand (ATO)	–10.891		O	HAD1	GLU129	H-bond	conventional H-bond
			NBF	OE2	GLU135	electrostatic	attractive charge
				SD	MET112	other	Pi-sulfur
				HG13	VA65	hydrophobic	Pi-sigma
			CAB		LEU114	hydrophobic	alkyl
			CAB		LEU126	hydrophobic	alkyl
			CAB		LEU198	hydrophobic	alkyl
			CAA		LEU103	hydrophobic	alkyl
			CAA		LEU198	hydrophobic	alkyl
		0.775			ALA78	hydrophobic	Pi-alkyl
					LEU181	hydrophobic	Pi-alkyl
					ILE194	hydrophobic	Pi-alkyl
					LEU157	hydrophobic	Pi-alkyl
					ALA78	hydrophobic	Pi-alkyl
					LEU181	hydrophobic	Pi-alkyl
					VA65	hydrophobic	Pi-alkyl
					LYS80	hydrophobic	Pi-alkyl
					MET112	hydrophobic	Pi-alkyl

a ATO: atovaquone.

Compound **4** established a strong conventional hydrogen bond with LEU57 residues (2.09 Å) and a Pi–Sulfur interaction with MET112 residues. Moreover, compound **4** established seven hydrophobic interactions (Pi-Alkyl) with LEU57, VA65, ALA78, LEU181, ILE194, and LYS80 residues (Table 2 and Figure 2).

**Figure 2:**
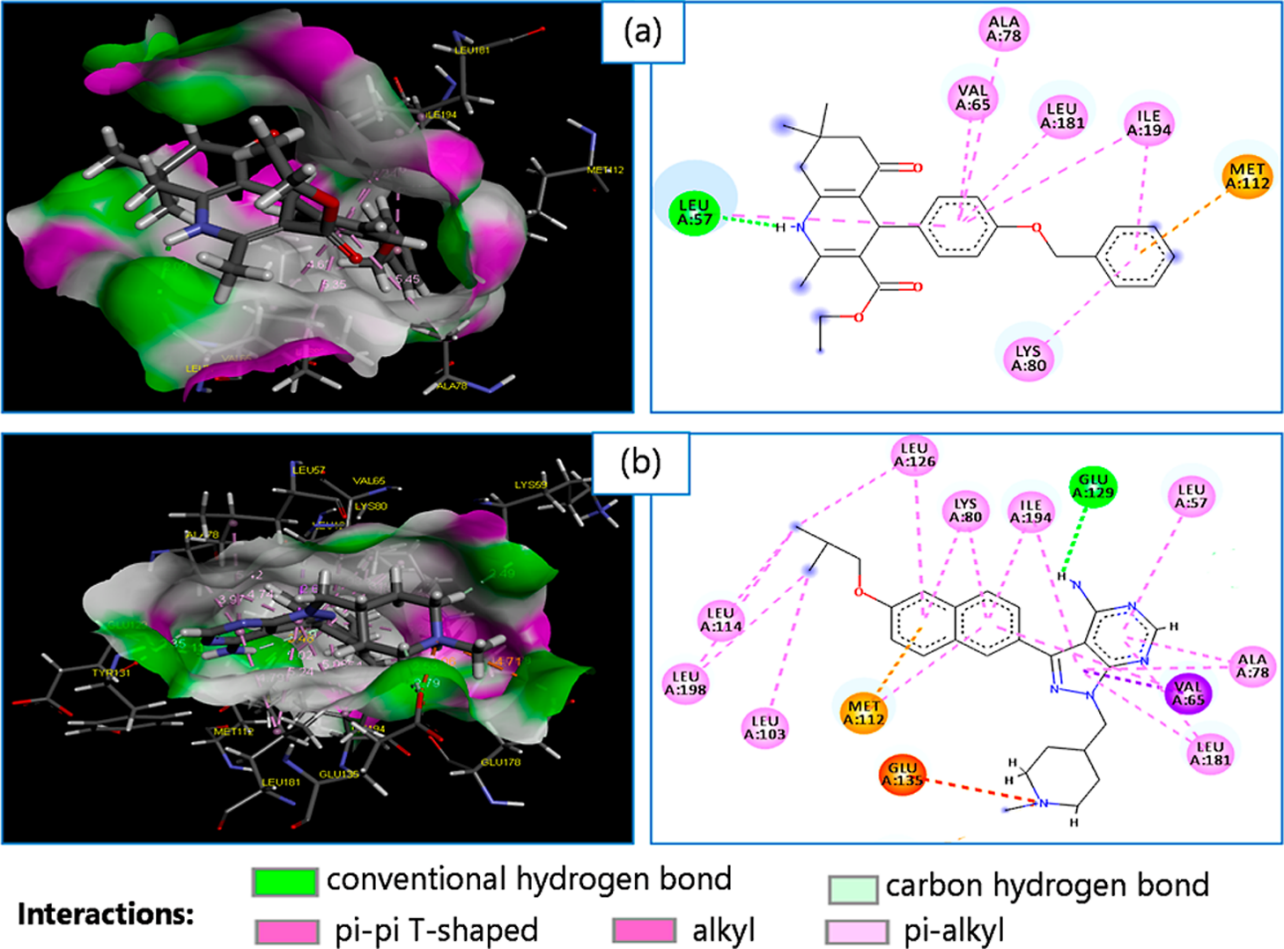
3D and 2D diagrams of interactions of the compound **4** (a) and its native ligand (b) with the active site residues of *Toxoplasma gondii* (PDB ID: 4JBV) target.

#### 3.4.3 Physicochemical Parameters and ADME-T Prediction

We evaluated the drug-likeness of compound **4** by using ADME-T predictions and calculating physicochemical properties. Table 3 summarizes the results.

**Table 3:** ADME-T and Drug-likeness Properties of Compound **4** ^a^

entry	TPSA (Å^2^)	*n*-ROTB	MW	MLog P	WLog P	*n*–ON acceptors	*n*–OHNH donors	Lipinski’s violations	Veber's violations	Egan's violations
	<140	<11	<500	≤5		<10	<5	≤1	≤1	≤1
4	64.63	7	445.55	3.36	4.90	4	1	accepted	accepted	accepted

	absorption		distribution		metabolism			excretion		toxicity	
ADME-T	Caco2 (10^–6^ cm/s)	HIA %	CNS (Log PS)	BBB (Log BB)	CYP1A2 inhibitor	CYP2C19 inhibitor	CYP2D6 substrate	renal OCT2 substrate	total clearance (mL/min/kg)	AMES toxicity	hepatotoxicity
4	1.04	96.17	–1.77	0.002	YES	YES	NO	NO	0.484	NO	YES

a TPSA: topological polar surface area, *n*-ROTB: number of rotatable bonds, MW: molecular weight, MLog*P*: logarithm of partition coefficient of compound between *n*-octanol and water, n-ON acceptors: number of hydrogen bond acceptors, n–OHNH donors: number of hydrogen bonds donors. Caco-2: colon adenocarcinoma, HIA: human intestinal absorption, CNS: central nervous system permeability, BBB: blood–brain barrier permeability. Renal OCT2 substrate: organic cation transporter 2.

Compound **4** had TPSA lower than 140 Å. Additionally, compound **6** had fewer than 5 n–OHNH donors and more than 10 n-ON acceptors. On the other hand, the molecular weight of this compound was lower than 500 g/mol, and its MLogP and WLogP were lower than 5. Besides, nROTB was less than 11.

Data presented in Table 3 indicates that (1) Compound **4** and ATO had Caco-2 greater than −5.15 cm/s; (2) Compound **6** had HIA (96.17%) greater than 30%; (3) The logPS of compound **4** lay in the −3 < logPS < −2 range; (4) the logBB of compound **4** and ATO was 0.119 and 0.002, respectively (Table 3); (5) Compound **4** inhibits CYP1A2 and CYP2C19 but not CYP2D6; (6) Neither compound **4** nor ATO are likely OCT2 substrates. Moreover, compound **4** has average excretion clearance (<5 mL/min/kg). (7) Compound **4** does not exhibit AMES toxicity, but it can be hepatotoxic.

## 4 Discussion

### 4.1 Synthesis of Compounds **1**–**24**

We synthesized compounds **1**– **24** by a “green” microwave-assisted one-pot multicomponent methodology. “Green chemistry” is on the rise as an environmentally friendly and sustainable way to prepare drug candidates to treat cancer and infectious diseases.^38^ The methodology used herein is attractive from the synthetic point of view: (1) compounds **1**– **24** are obtained in only one synthetic step (i.e., a one-pot Hantzsch multicomponent reaction); (2) the methodology employs nontoxic solvents, (3) the reaction times are short (about 30 min), (4) the products are solid and easy to isolate (i.e., dismissing the need for time-demanding chromatographic steps), and yields range from 15% to 40% due to formation of byproducts (e.g., pyridines,^39^ 1,2-dihydropyridines,^39^ and acridine-1,8-diones,^40^ which were detected in the ethanol-soluble phase of recrystallization; data not shown).

### 4.2 Antileishmanial Activity of Compounds **1**–**24** against *L. major*

The antileishmanial activity of 1,4-DHPs against *L. amazonensis* has been extensively exploited.^1,14^ While *L. amazonensis* is the main *Leishmania* species responsible for CL in the New World (i.e., South America), *L. major* is known to cause CL in the Old World (i.e., Central Asia, the Middle East, and Africa).^41^ Based on the different susceptibility of *L. amazonensis* to primary *S*-nitrosothiols^42^ and azithromycin,^43^ miltefosine, edelfosine, and amphotericin B^44^ compared to other *Leishmania* species, we investigated the in vitro antileishmanial activity of compounds **1–24** against *L. major* promastigotes and amastigotes.

In general, compounds with IC_50_ lower than 10 μM, between 10 and 50 μM, between 50 and 100 μM, and higher than 100 μM are considered very active, active, moderately active, and inactive, respectively.^16^ Based on these criteria, we found that compounds **12**, **14**, **15**, **22**, and **24** are active against extracellular *L. major* promastigotes. Oliveira and co-workers reported that compounds **12**, **14**, **22**, and **24** are inactive against *L. amazonensis* promastigotes.^1^ Conversely, among the most active compounds against *L. amazonensis* promastigotes (compounds **13**, IC_50_ = 24.62 μM) and amastigotes (compounds **7** and **9**, IC_50_ = 12.53 and 13.67 μM, respectively),^1^ only compound **9** (IC_50_ = 13.67 μM) is active against *L. major* amastigotes at concentrations lower than 50 μM. Escobar and co-workers described different sensitivities of *Leishmania* promastigotes and amastigotes to miltefosine, edelfosine, and amphotericin B, which are among the drugs that are currently used to treat leishmaniasis.^1,44^ Intrinsic variations in the sensitivity of these two *Leishmania* species to drugs have important implications for clinical treatments.^44^ On the other hand, among the compounds tested here, we found that only compounds **4**, **9**, **10**, **14**, and **16** are active against intracellular *L. major* amastigotes. Interestingly, we verified that none of the symmetric compounds (compounds **17**– **24**) are active against *L. major* amastigotes. When analyzed together, these data suggest that the presence of fluorine (in compound **9**), a nitro (in compounds **10** and **22**), or an aromatic ether (in compounds **4**, **12**, **14**, **15**, and **24**) is essential for the antileishmanial activity against *L. major* promastigotes and amastigotes. Indeed, fluorophenyl has been identified as a key feature in the structure of other compounds that display potent antileishmanial activity against *L. major*,^4,45,46^ such as 4-amino-2-(3-fluorophenyl)-1,2-dihydropyrimido­[1,2-*a*]­benzimidazole-3-carbonitrile.^4^ Similarly, nitroaromatic compounds **10** and **22** have also been reported to be effective against *L. major*.^47^ The antileishmanial activity of these compounds may be related to their ability to act as redox-active agents and to increase ROS (reactive oxygen species) generation in *Leishmania* parasites, to dissipate the mitochondrial potential.^48^ In the case of the methoxylated compounds **14** and **24**, Genestra and co-workers demonstrated the role played by methoxy groups in the antileishmanial activity.^49^ However, only the presence of the methoxy group does not ensure antileishmanial activity, as evidenced by the inactivity of compounds **3**, **11**, **18**, and **23**. Indeed, combined with other structural features (e.g., the symmetry of the 1,4-DHP structure core), the number and location of these substituents at the aromatic ring may affect the antileishmanial activity significantly. Mohammadi-Ghalehbin reported a similar finding for *n*-aryl enamino amide derivatives.^50^ Compounds bearing benzyloxy and methylenodioxy substituents in their structures other than compounds **4** and **12** have been described as potent antileishmanial agents.^45,51^

The selectivity index (SI) is an essential parameter when developing new chemotherapeutic agents: SI allows the toxicity of the tested compounds to normal cells to be evaluated and compared to the target cells and helps to predict their therapeutic potential.^52^ In the case of the antileishmanial activity, the IC_50_ of compounds **1**–**24** against Vero cells is generally lower than the IC_50_ of these compounds against *L. major* promastigotes and amastigotes and macrophages. This gives SI lower than 1, which shows poor selectivity for *L. major* promastigotes and amastigotes over Vero cells. Therefore, despite the promising activity of compounds **14** and **9** against *L. major* promastigotes and amastigotes, further docking studies were not carried out because of their poor selectivity over Vero cells.

### 4.3 Antitoxoplasmal Activity of Compounds **1**–**24** against *T. gondii*

Most of the 1,4-DHPs that proved active against *T. gondii* are asymmetric (i.e., derived from dimedone), also known as “polyhydroquinolines”. On the other hand, some symmetric 1,4-DHPs (commonly known as “Hantzsch esters”) were also active (IC_50_ < 50 μM), such as compounds **17**, **22**, and **23**. Therefore, the activity of 1,4-DHPs cannot be understood in terms of the type of 1,4-DHP only. On the other hand, the presence of particular substituents in specific positions of the aromatic ring was found to be important for the antitoxoplasmal activity of 1,4-DHPs, such as the groups 3-NO_2_ (for compounds **10** and **22**) and 3-OMe/4-OH (for compounds **11** and **23**). Although these groups have also been found in the structure of other compounds active against *T. gondii*,^53^ their role in the antitoxoplasmal activity was not investigated more deeply in this study.

### 4.4 Computational Approach

Molecular docking is a computational strategy that predicts the precise alignment of a ligand molecule with a receptor protein, resulting in a stable complex formation. This alignment is essential for determining the binding affinity and strength of the ligand–protein interaction, accomplished through a scoring function.^54^ The interaction between a drug and its receptor lowers the overall free energy of the system, offering critical insights into the molecule’s affinity and activity, which are vital for advancing drug design and discovery.^55^ In this study, we have investigated the complex formed after docking of compound **4** with the *T. gondii* target (4JBV). Furthermore, ADME-T predictions indicated that this compound meets the drug-likeness criteria without violating any Lipinski, V). This target belongs to a family of Calcium-Dependent Protein Kinases (CDPKs) that regulate the complex life cycle of *T. gondii*, and it has been widely used in research for new therapeutics against *T. gondii*.^56^ The compound **4**: 4JBV). Furthermore, ADME-T predictions indicated that this compound meets the drug-likeness criteria without violating any Lipinski, V complex had an energy score of −7.174 kcal/mol, which was slightly closer to the energy score of the native ligand (ATO) (energy score = −10.891 kcal/mol). A Pi-sulfur interaction with MET112 residues interaction and seven hydrophobic interactions Pi-Alkyl with LEU57, VA65, ALA78, LEU181, ILE194, and LYS80 residues was observed (Table 2 and Figure 2). Previous studies indicated that MET112, VA65, ILE194, and LYS80 residues are the key to the inhibition of *T. gondii* target (4JBV). Furthermore, ADME-T predictions indicated that this compound meets the drug-likeness criteria without violating any Lipinski, V).^31,57^ Likewise, molecular docking results showed that the binding types of compound **4** with the *T. gondii* target (PDB ID: 4JBV) were similar to the native ligand (ATO), which is justified by the presence of several common residues.

The drug-like behavior of compound **4** was also estimated based on how physicochemical properties fit Lipinski’s “rule-of-five” (Table 3). According to this rule, an orally active drug-like compound should not have more than one violation of the following criteria: hydrogen bond donors not greater than 5, hydrogen bond acceptors not greater than 10, molecular weight not greater than 500 Da, and an octanol–water partition coefficient (log P) not greater than 5.^58,59^ As shown in Table 3, compound **4** complies with the drug-likeness criteria without violating Lipinski, Veber, or Egan’s rules.^59,60^

The ADME-T (absorption, distribution, metabolism, excretion, and toxicity) properties of compound **4** were also predicted.^61^ Based on the data presented in Table 3, it is shown that (1) compound **4** has good human intestinal permeability (Caco-2 greater than −5.15 cm/s); (3) compound **4** can be easily absorbed by the gastrointestinal system after it is orally administered, as it has HIA (96.17%) greater than 30%; (4) compound **4** can penetrate the CNS (−3 < logPS < −2); (5) Compound **4** (logBB = 0.119) and ATO (logBB = 0.002) are poorly distributed in the brain (logBB < −10); (6) Compound **4** inhibits CYP1A2 and CYP2C19 but not CYP2D6; (7) Neither compound **4** nor ATO are likely OCT2 substrates (8) Compound **4** has average excretion clearance (<5 mL/min/kg). Based on the data presented in Table 3, it is also shown that compound **4** does not exhibit AMES toxicity, but it can be hepatotoxic.

## 5 Conclusions

We have found that seven 1,4-dihydropyridines display good antiparasitic activity against *L. major* promastigotes and amastigotes, albeit without selectivity for these parasites over Vero and macrophage cells. Compound **4** is active against *T. gondii* and about five times more selective for the parasite cells than for Vero cells. Docking studies revealed that compound **4** has a strong affinity for the *T. gondii* target (PDB ID: 4JBV). Furthermore, ADME-T predictions indicated that this compound meets the drug-likeness criteria without violating any Lipinski, Veber, or Egan rules.

Our results reinforce the antiparasitic potential of 1,4-DHPs. Structural modification-based strategies should be adopted in future works to increase the selectivity for *T. gondii* and *L. major*.

## Supplementary Material



## Notes

The following files are available free of charge. The NMR and MS spectra of compounds **1–24** (PDF)

## Abbreviations

1: 4-DHP 1,4-dihydropyridine
ADME-T: absorption, distribution, metabolism, excretion, and toxicity
AmB: amphotericin B
ATO: atovaquone
BALB: Bagg Albino Laboratory Bred
CL: cutaneous leishmaniasis
CPCM: conductor-like polarizable continuum model
DEPT: distortionless enhancement by polarization transfer
DFT: density functional theory
DHFR: dihydrofolate reduyctase
DMSO: dimethyl sulfoxide
EC_50_: concentration causing 50% of the maximum effect
FBS: fetal bovine serum
IC_50_: half inhibitory minimal concentration
LogP: logarithm of octanol/water partition coefficient
MCL: mucocutaneous leishmaniasis
MOE: molecular operating environment
MTT: 3-(4,5-dimethylthiazol-2-yl)-2,5-diphenyltetrazolium bromide
MW: molecular weight
nHA: number of hydrogen bond acceptors
nHD: number of hydrogen bond donors
NMR: nuclear magnetic resonance
nROT: number of rotable bonds
NTD: neglected tropical diseases
PBS: phosphate-buffered saline
PDB: Protein Data Bank
RH: strain: right heart strain
RMSD: root mean square deviation
RPMI: Roswell Park Memorial Institute
SI: selectivity index
TPSA: topological polar surface area
VL: visceral leishmaniasis.
