# Supplementary material for: Antileishmanial and Antitoxoplasmal Activities of 1,4-Dihydropyridines
Source: ACS Omega. 2025 Jul 10;10(28):31066–76. doi: 10.1021/acsomega.5c04551 (PMC12290673; doi:10.1021/acsomega.5c04551)
Supplement: Supplementary file 1 [file ao5c04551_si_001.pdf]

Supporting Information

Antileishmanial and Antitoxoplasmal Activities of 1,4-dihydropyridines

Thaís A. S. Oliveira [1], Yan R. Robles[1], Ibrahim S. Al Nasr [2], Waleed S. Koko[2], Tariq A. Khan [3], Ismail Daoud[4,5], Seyfeddine Ra-hali[6], Noureddine Amdouni[7], Ridha B. Said [6], and Antônio E. M. Crotti [1,]*

1 Department of Chemistry, Faculty of Philosophy, Sciences, and Letters at Ribeirão Preto, University of São Paulo, 14900-001, Ribeirão Preto, SP-Brazil

2 Department of Biology, College of Science, Qassim University, Qassim 51452, Saudi Arabia

3 Department of Basic Health Sciences, College of Applied Medical Sciences, Qassim University, Qassim 51452, Saudi Arabia

4 Department of Matter Sciences, University Mohamed Khider, BP 145 RP, Biskra 07000, Algeria

5 Laboratory of Natural and Bio-Active Substances, Faculty of Science, Tlemcen University, Tlemcen P.O. Box 119, Algeria

6 Department of Chemistry, College of Science, Qassim University, Qassim 51452, Saudi Arabia

7 Laboratoire de Caractérisations, Applications et Modélisations des Matériaux, Faculté des Sciences de Tunis, Université Tunis El Manar, Tunis 1068, Tunisia

* Correspondence: millercrotti@ffclrp.usp.br (A.E.M.C.)

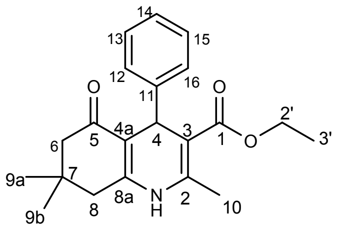

1

(±)-ethyl 2,7,7-trimethyl-5-oxo-4-phenyl-1,4,5,6,7,8-hexahydroquinoline-3-carboxylate (**1**). White powder, 31% yield. Purity (HPLC): > 95% (**Fig. S1**). ^{1}H NMR (400 MHz, *CDCl$_3$*, **Fig. S2**): *δ* 0.98 (3H, s, H9b), 1.11 (3H, *s*, H9a), 1.25 (3H, *t*, *J* = 7.0 Hz, H3′), 2.15-2.35 (4H, *m*, H6 and H8), 2.40 (3H, *s*, H10), 4.12 (2H, *q*, *J*= 6.8 Hz, H2′), 5.10 (1H, *s*, H4), 7.10 – 7.40 (5H, *m*, H12=16, H13=H15, and H14).^{13}C NMR (100 MHz, *CDCl$_3$*, **Fig. S3** and **S4**): *δ* 14.0 (CH$_3$, C3′), 19.2 (CH$_3$, C10), 27.0 (CH$_3$, C9a), 29.2 (CH$_3$, C9b), 32.5 (C, C7), 36.5 (CH, C4), 41.0 (CH$_2$, C8), 50.8 (CH$_2$, C6), 59.7 (CH$_2$, C2′), 105.9 (C, C3), 111.9 (C, C4a), 125.8 (CH, C14), 127.8 (CH, C12=C16, C13=C15), 143.4 (C, C11), 146.9 (C, C8a), 148.4 (C, C2), 167.7 (C1′), 195.6 (C, C5). ESI(+)-MS (**Fig. S5**): *m/z* 378 (5%, [M+K]$^+$), *m/z* 362 (49%, [M+Na]$^+$), *m/z* 340 (100%, [M+H]$^+$), *m/z* 262 (15%, [M+H-C$_6$H$_6$]$^+$).

(±)-ethyl 2,7,7-trimethyl-5-oxo-4-(p-tolyl)-1,4,5,6,7,8-hexahydroquinoline-3-carboxylate (2). Yellowish powder, 33% yield. Purity (HPLC): > 95% (**Fig. S6**). ^{1}H NMR (400 MHz, *CDCl$_3$*, **Fig. S7**): δ 0.94 (3H, *s*, H9b), 1.07 (3H, *s*, H9a), 1.21 (3H, *t*, *J* = 7.1 Hz, H3′), 2.13-2.30 (4H, *m*, H6 and H8), 2.25 (3H, *s*, H17), 2.35 (3H, *s*, H10), 4.05 (2H, *q*, *J*= 7.1 Hz, H2′), 5.01 (1H, *s*, H4), 6.99 (2H, *d*, *J*=7.9 Hz, H13=H15), 7.18 (2H, *d*, *J*=7.9 Hz, H12=H16). ^{13}C (400 MHz, *CDCl$_3$*, **Fig. S8** and **S9**): δ 14.0 (CH_3, C3′), 19.3 (CH_3, C10), 20.9 (CH_3, C17), 27.1 (CH_3, C9a), 29.2 (CH_3, C9b), 32.6 (C, C7), 35.9 (CH, C4), 40.9 (CH_2, C8), 50.5 (CH_2, C6), 59.6 (CH_2, C2′), 106.1 (C, C3), 112.2 (C, C4a), 127.7 (CH, C13=C15), 128.4 (CH, C12=C16), 135.2 (C, C14), 143.0 (C, C11), 143.9 (C, C8a), 147.7 (C, C2), 167.3 (C, C1), 195.3 (C, C5). ESI(+)-MS (**Fig. S10**): *m/z* 392 (5%, [M+K]$^+$), *m/z* 376 (90%, [M+Na]$^+$), *m/z* 354 (100%, [M+H]$^+$), *m/z* 262 ([M+H-C_7H_7]$^+$).

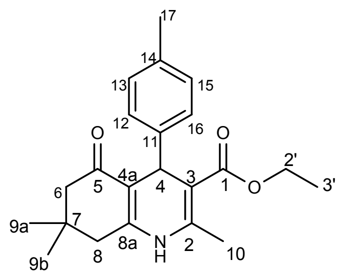

2

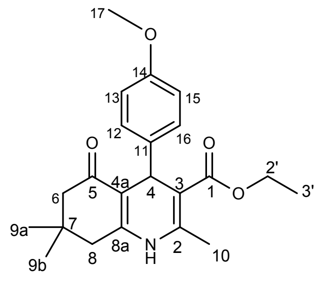

3

(±)-ethyl 4-(4-methoxyphenyl)-2,7,7-trimethyl-5-oxo-1,4,5,6,7,8-hexahydroquinoline-3-carboxylate (**3**). Yellowish powder, 35% yield. Purity (HPLC): > 95% (**Fig. S11**). NMR ^{1}H (400 MHz, *CDCl$_3$*, **Fig. S12**): δ 0.93 (3H, s, H9b), 1.07 (3H, *s*, H9a), 1.20 (3H, *t*, *J* = 7.1 Hz, H3′), 2.13-2.34 (4H, *m*, H6 and H8), 2.36 (3H, *s*, H10), 3.73 (3H, *s*, H17) 4.04 (2H, *q*, *J*= 7.1 Hz, H2′), 4.99 (1H, *s*, H4), 6.74 (2H, *d*, *J*=8.7 Hz, H13=H15), 7.21(2H, *d*, *J*=8.7 Hz, H12=H16).^{13}C (400 MHz, *CDCl$_3$*, **Fig. S13** and **S14**): δ 14.1 (CH_3, C3′), 19.2 (CH_3, C10), 27.1 (CH_3, C9a), 29.2 (C9b CH_3,), 32.4 (C, C7), 35.6 (CH, C4), 41.1 (CH_2, C8), 50.5 (CH_2, C6), 54.9 (CH_3, C17), 60.0 (CH_2, C2′), 106.3 (C, C3), 112.3 (C, C4a), 113.3 (CH, C13=C15), 129.1 (CH, C12=C16), 139.5 (C, C11) , 143.0 (C, C8a) , 147.4 (C, C2), 157.6 (C, C14), 167.4 (C, C1′), 197.6 (C, C5). ESI(+)-MS (**Fig. S15**): *m/z* 408 (5%, [M+K]$^+$), *m/z* 392 (100%, [M+Na]$^+$), *m/z* 370 (80%, [M+H]$^+$), *m/z* 262 (100%, [M+H-C_7H_8O]$^+$).

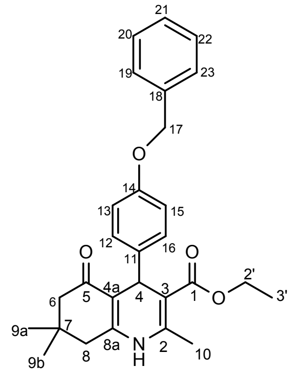

4

(±)-ethyl 4-(4-(benzyloxy)phenyl)-2,7,7-trimethyl-5-oxo-1,4,5,6,7,8-hexahydroquinoline-3-carboxylate (**4**). Yellowish powder, 33% yield. Purity (HPLC): > 95% (**Fig. S16**). ^{1}H NMR (400 MHz, *CDCl$_3$*, **Fig. S17**): δ 0.94 (3H, *s*, H9b), 1.07 (3H, *s*, H9a), 1.20 (3H, *t*, *J* = 7.1 Hz, H3′), 2.13-2.30 (4H, *m*, H6 and H8), 2.37 (3H, *s*, H10), 4.05 (2H, *q*, *J*=7.1 Hz, H2′), 4.98 (2H, *s*, H17), 5.00 (1H, *s*, H4), 6.81 (2H, *d*, *J* = 8.5 Hz, H13=H15), 7.21 (2H, *d*, *J* = 8.5 Hz, H12=H16), 7.30-7.41 (5H, *m*, H19=H23, H20=H22, and H23).^{13}C NMR (100 MHz, *CDCl$_3$*, **Fig. S18** and **S19**): δ 14.2 (CH_3, C3′), 19.7 (CH_3, C10), 27.4 (CH_3, C9a), 29.3 (CH_3, C9b), 32.8 (C, C7), 35.8 (CH, C4), 41.1 (CH_2, C8), 50.8 (CH_2, C6), 59.9 (CH_2, C2′), 70.0 (CH_2, C17), 106.4 (C, C3), 112.4 (C, C4a), 114.2 (CH, C13=C15), 128.6 (CH, C19=C23), 129.1 (CH, C21), 137.4 (CH, C20=C22), 140.0 (CH, C12=C16), 143.4 (C, C11=C18), 148.2 (C, C8a), 157.2 (C, C2),

167.7 (C, C1), 195.9 (C, C5). ESI(+)-MS (**Fig. S20**): *m/z* 484 (5%, [M+K]$^+$), *m/z* 468 (50%, [M+Na]$^+$), *m/z* 446 (40%, [M+H]$^+$), *m/z* 262 ([M+H-$C_{13}H_{12}O$]$^+$).

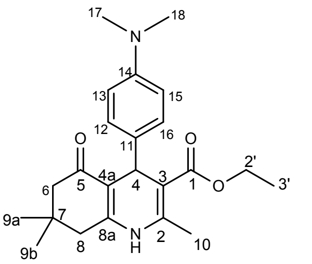

5

(±)-ethyl 4-(4-(dimethylamino)phenyl)-2,7,7-trimethyl-5-oxo-1,4,5,6,7,8-hexahydroquinoline-3-carboxylate (**5**). Yellow powder, 31% yield. Purity (HPLC): > 95% (**Fig. S21**). ^{1}H NMR (400 MHz, *CDCl$_3$*, **Fig. S22**): δ 0.96 (3H, *s*, H9b), 1.07 (3H, *s*, H19a), 1.22 (3H, *t*, *J* = 7.0 Hz, H3′), 2.17-2.33 (4H, *m*, H6 and H8), 2.35 (3H, *s*, H10), 2.86 (6H, *s*, H17=H18), 4.04 (2H, *q*, *J*=7.0 Hz, H2′), 4.94 (1H, *s*, H4), 6.57 (2H, *d* , *J*= 8.8 Hz, H13=H15), 7.15 (2H, *d*, *J*= 8.8 Hz, H12=H16).^{13}C NMR (100 MHz, *CDCl$_3$*, **Fig. S23** and **24**): δ 14.4 (CH_3, C3′), 19.4 (CH_3, C10), 27.4 (CH_3, C9a), 29.3 (CH_3, C9b), 32.7 (C, C7), 35.4 (CH, C4), 40.7 (CH_3, C17=C18), 40.9 (CH_2, C8), 50.9 (CH_2, C6), 59.9 (CH_2, C2′), 106.4 (C, C3),112.4 (C, C4a), 112.5 (CH, C12=C16), 128.9 (CH, C13=C15), 135.9 (C, C11), 143.4 (C, C14), 148.2 (C, C8a), 149.1 (C, C2), 167.9 (C, C1′), 196.1 (C, C5). ESI(+)-MS (**Fig. S25**): *m/z* 405 (35%, [M+Na]$^+$), *m/z* 383 (40%, [M+H]$^+$), *m/z* 262 (100%, [M+H-$C_6H_5NMe_2$]$^+$).

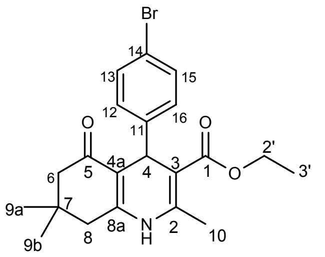

6

(±)-ethyl 4-(4-bromophenyl)-2,7,7-trimethyl-5-oxo-1,4,5,6,7,8-hexahydroquinoline-3-carboxylate (**6**). Yellowish powder, 39% yield. Purity (HPLC): > 95% (**Fig. S26**). NMR ^{1}H (400 MHz, *CDCl$_3$*, **Fig. S27**): δ 0.92 (3H, *s*, H9a), 1.06 (3H, *s*, H9b), 1.19 (3H, *t*, *J* = 7.1 Hz, H3′), 2.12-2.29 (4H, *m*, H6 and H8), 2.36 (3H, *s*, H10), 4.04 (2H, *q*, *J*=7.1 Hz, H2′), 5.00 (1H, *s*, H4), 7.17 (2H, *d* , *J*= 8.4 Hz, H12=H16), 7.30 (2H, *d*, *J*= 8.4 Hz, H13=H15).^{13}C NMR (100 MHz, *CDCl$_3$*, **Fig. S28** and **29**): δ 14.7 (CH_3, C3′), 19.9 (CH_3, C10), 27.5 (CH_3, C9a), 29.9 (CH_3, C9b), 33.2 (C, C7), 36.7 (CH, C4) , 41.5 (CH_2, C8), 51.1 (CH_2, C6), 60.2 (CH_2, C2′), 106.1 (C, C3), 112.2 (C, C4a), 120.2 (C, C14), 130.3 (CH, C12=C16), 131.4 (CH, C13=C15), 144.1 (C, C11), 146.5 (C, C8a), 148.7 (C, C2), 167.6 (C, C1) ,195.9 (C, C5). ESI(+)-MS (**Fig. S30**): *m/z* 440/442 (30%, [M+Na]$^+$), *m/z* 418/420 (100%, [M+H]$^+$), *m/z* 262 (50%, [M+H-C_6H_5Br]$^+$).

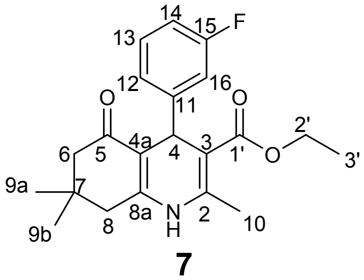

7

(±)-ethyl 4-(3-fluorophenyl)-2,7,7-trimethyl-5-oxo-1,4,5,6,7,8-hexahydroquinoline-3-carboxylate (**7**). White powder, 39% yield. Purity (HPLC): > 95% (**Fig. S31**). ^{1}H NMR (400 MHz, *CDCl$_3$*, **Fig. S32**): δ 0.94 (3H, s, H9b), 1.08 (3H, *s*, H9a), 1.19 (3H, *t*, *J* = 7.1 Hz, H3′), 2.30-2.43 (4H, *m*, H6 and H8), 2.38 (3H, *s*, H10), 4.06 (2H, *q*, *J*=7.1 Hz, H2′), 5.06 (1H, *s*, H14), 6.79 (1H, *t*, *J*= 7.8 Hz, H13), 6.97 (1H, *d*, *J*= 9.9 Hz H12), 7.08-7.18 (2H, *m*, H14 and H16) .^{13}C NMR (100 MHz, *CDCl$_3$*, **Fig. S33** and **S34**): δ 14.2 (CH_3, C3′), 19.7 (CH_3, C10), 27.0 (CH_3, C9a), 29.2 (CH_3, C9b), 32.8 (C, C7), 35.7 (CH, C4), 41.1 (CH_2, C8), 50.8 (CH_2, C6), 60.1 (CH_2, C2′), 105.7 (C, C3),111.6 (C, C4a), 112.5 (CH, C16), 114.4 (CH, C13),

122.2 (C, C11), 129.8 (CH, C14), 143.7 (CH, C12), 148.5 (C, C8a), 149.3 (C, C2), 163.9 (C, C15), 167.4 (C, C1′),195.7 (C, C5). ESI(+)-MS (**Fig. S35**): *m/z* 380 (45%, $[M+Na]^+$), *m/z* 358 (100%, $[M+H]^+$), *m/z* 262 (30%, $[M+H-C_6H_5F]^+$).

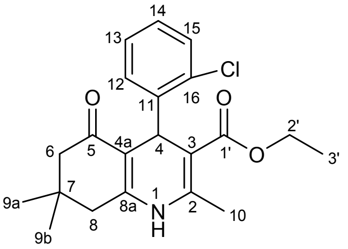

8

(±)-ethyl 4-(2-chlorophenyl)-2,7,7-trimethyl-5-oxo-1,4,5,6,7,8-hexahydroquinoline-3-carboxylate (**8**). White powder, 40% yield. Purity (HPLC): > 95% (**Fig. S36**). ^{1}H NMR (400 MHz, *CDCl*$_3$, **Fig. S37**): *δ* 0.93 (3H, *s*, H9a), 1.08 (3H, *s*, H9b), 1.19 (3H, *t*, *J* = 7.1 Hz, H3′), 2.12-2.36 (4H, *m*, H6 and H8), 2.40 (3H, *s*, H10), 4.05 (2H, *q*, *J*= 7.1 Hz, H2′), 5.15 (1H, *s*, H4), 7.70 (1H, *d*, *J*= 7.7 Hz, H12), 7.91 (1H, *t*, *J* = 7.9 Hz, H13), 7.97 (1H, *d*, *J*=8.2 Hz, H15), 8.10 (1H, *t*, *J*= 1.9 Hz, H16).^{13}C NMR (100 MHz, *CDCl*$_3$, **Fig. S38** and **S39**): *δ* 14.0 (CH_3, C3′), 19.4 (CH_3, C10), 26.9 (CH_3, C9a), 29.2 (CH_3, C9b), 32.6 (C, C7) , 36.8 (CH, C4), 40.91 (CH_2, C8), 50.4 (CH_2, C6), 59.9 (CH_2, C2′), 105.0 (C, C3), 111.2 (C, C4a), 121.1 (CH, C12), 122.6 (CH, C13), 128.4 (CH, C14), 134.7 (CH, C15), 144.03 (C, C16), 148.2 (C, C8a) , 148.9 (C, C11), 157 (C, C2), 166.7 (C, C1′), 195.2 (C, C5). ESI(+)-MS (**Fig. S40**): *m/z* 396 (100%, $[M+Na]^+$), *m/z* 374 (100%, $[M+H]^+$), *m/z* 262 (50%, $[M+H-C_6H_5Cl]^+$).

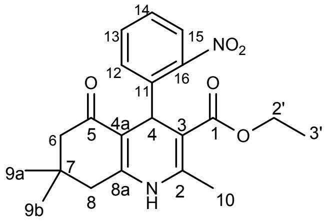

9

(±)-ethyl 2,7,7-trimethyl-4-(2-nitrophenyl)-5-oxo-1,4,5,6,7,8-hexahydroquinoline-3-carboxylate (**9**). Yellowish powder, 38% yield. Purity (HPLC): > 95% (**Fig. S41**). ^{1}H NMR (400 MHz, *CDCl*$_3$, **Fig. S42**): *δ* 0.93 (3H, *s*, H9b), 1.08 (3H, *s*, H9a), 1.18 (3H, *t*, *J* = 7.1 Hz, H3′), 2.11-2.35 (4H, *m*, H6 andH8), 2.39 (3H, *s*, H10), 4.03 (2H, *q*, *J*=7.1 Hz, H2′), 5.14 (1H, *s*, H4), 7.36 (1H, *t*, *J*= 7.9 Hz, H14), 7.70 (1H, *d*, *J*= 7.7 Hz, H12) 7.99 (1H, *d*, *J*= 8.2 Hz, H15), 8.10 (1H, *t*, *J*= 8.4 Hz, H=13).^{13}C NMR (100 MHz, *CDCl*$_3$, **Fig. S43** and **S44**): *δ* 13.9 (CH_3, C3′), 19.4 (CH_3, C10), 26.8 (CH_3, C9a), 29.3 (CH_3, C9b), 32.9 (C, C7), 37.1 (CH, C4), 40.9 (CH_2, C8), 50.7 (CH_2, C6), 60.2 (CH_2, C2′), 104.8 (C, C3), 111.2 (C, C4a), 121.2 (CH, C15), 122.5 (CH, C14), 128.3 (CH, C12) , 134.7 (CH, C13), 144.1 (C, C11), 148.2 (C, C8a), 148.9 (C, C16), 166.5 (C, C1), 195.2 (C, C5). ESI(+)-MS (**Fig. S45**): *m/z* 407 (55%, $[M+Na]^+$), *m/z* 385 (100%, $[M+H]^+$).

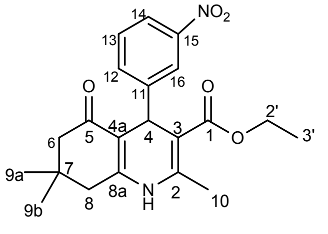

10

(±)-ethyl 2,7,7-trimethyl-4-(3-nitrophenyl)-5-oxo-1,4,5,6,7,8-hexahydroquinoline-3-carboxylate (**10**). White powder, 30% yield. Purity (HPLC): > 95% (**Fig. S46**). ^{1}H NMR (400 MHz, *CDCl*$_3$, **Fig. S47**): *δ* 0.93 (3H, *s*, H9b), 1.09 (3H, s, H9a), 1.19 (3H, *t*, *J* = 7.1 Hz, H3′), 2.12-2.36 (4H, *m*, H6 and H8),), 2.40 (3H, *s*, H10), 4.05 (2H, *q*, *J*=7.3 Hz, H2′), 5.15 (1H, s, H4), 7.41 (1H, *t* , *J*= 7.9 Hz, H14), 7.72 (1H, *d*, *J*= 7.7 Hz, H12) 7.97 (1H, *d*, *J*= 8.2 Hz, H15), 8.10 (1H, *t*, *J*=1.4 Hz, H13).^{13}C NMR (100 MHz, *CDCl*$_3$, **Fig. S48** and **S49**): *δ* 14.2 (CH_3, C3′), 18.6 (CH_3, C10), 27.1 (CH_3, C9a), 29.6 (CH_3, C9b), 32.6 (C, C7), 35.4

(CH, C4), 41.6 (CH_2, C8), 51.0 (CH_2, C6), 59.5 (CH_2, C2′), 104.2 (C, C3), 110.6 (C, C4a), 121.2 (CH, C14), 122.5 (CH, C12), 128.3 (CH, C13) , 134.7 (CH, C16), 143.1 (C, C15), 147.9 (C, C11), 148.5 (C, C8a), 159.1 (C, C2), 168.4 (C, C1′), 195.7 (C, C5). ESI(+)-MS (**Fig. S50**): *m/z* 378 (5%, $[M+K]^+$), *m/z* 362 (70%, $[M+Na]^+$), *m/z* 340 (15%, $[M+H]^+$), *m/z* 262 (100%, $[M+H-C_6H_5NO_2]^+$).

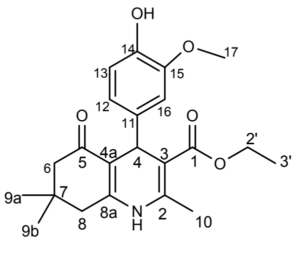

11

(±)-ethyl 4-(4-hydroxy-3-methoxyphenyl)-2,7,7-trimethyl-5-oxo-1,4,5,6,7,8-hexahydroquinoline-3-carboxylate (11). Orangish powder, 35% yield. Purity (HPLC): > 95% (**Fig. S51**). ^1H NMR (400 MHz, *CDCl₃*, **Fig. S52**):): *δ* 0.94 (3H, *s*, H9b), 1.07 (3H, *s*, H9a), 1.21 (3H, *t*, *J* = 7.1 Hz, H3′), 2.14-2.34 (4H, *m*, H6 and H8), 2.36 (3H, *s*, H10), 3.84 (3H, *s*, H17), 4.08 (2H, *q*, *J*= 7.1 Hz, H2′), 4.96 (1H, *s*, H4), 6.69 (1H, *dd*, *J*=8.2 Hz, H12), 6.72 (1H, *d*, *J*=8.1 Hz, H13), 6.92 (1H, *d*, *J*=1.3 Hz, H16). ^{13}C NMR (400 MHz, *$CDCl_3$*, **Fig. S53** and **S54**): *δ* 14.4 (CH_3, C3′), 19.5 (CH_3, C10), 27.1 (CH_3, C9a), 29.6 (CH_3, C9b), 32.7 (C, C7), 36.0 (CH, C4), 41.3 (CH_2, C8), 50.8 (CH_2, C6), 55.9 (CH_2, C2′), 59.7 (CH_3, C17), 106.3 (C, C3), 111.3 (C, C4a), 112.3 (CH, C16) , 113.9 (CH, C13), 119.9 (CH, C12), 139.2 (C, C11), 143.3 (C, C14), 145.5 (C, C15), 146.9 (C, C8a), 147.8 (C, C2), 167.4 (C, C1), 195.6 (C, C5). ESI(+)-MS (**Fig. S55**): *m/z* 408 (40%, $[M+Na]^+$), *m/z* 386 (20%, $[M+H]^+$), *m/z* 262 (100%, $[M+H-C_7H_8O_2]^+$).

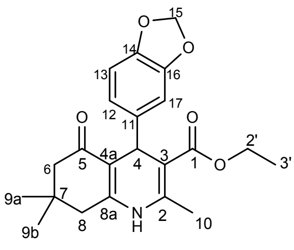

12

(±)-ethyl 4-(benzo[*d*][1,3]dioxol-5-yl)-2,7,7-trimethyl-5-oxo-1,4,5,6,7,8-hexahydroquinoline-3-carboxylate (**12**). White powder, 32% yield. Purity (HPLC): > 95% (**Fig. S56**). ^1H NMR (400 MHz, *$CDCl_3$*, **Fig. S57**): *δ* 0.96 (3H, *s*, H9b), 1.07 (3H, *s*, H9a), 1.21 (3H, *t*, *J* = 7.1 Hz, H21), 2.14-2.30 (4H, *m*, H6 and H8), 2.35 (3H, s, H10), 4.07 (2H, *q*, *J*= 7.1, Hz, H2′), 4.97 (1H, s, H4), 5.86 (2H, *d* *J*=1.4 Hz, H15), 6.63 (1H, *d*, *J*=7.9 Hz, H13), 6.76-6.80 (2H, *m*, H12 and H17). ^{13}C NMR (100 MHz, *$CDCl_3$*, **Fig. S58** and **S59**): *δ* 14.1 (CH_3, C3′), 19.2 (CH_3, C10), 27.10 (CH_3, C9a), 29.2 (CH_3, C9b), 32.5 (C, C7), 36.1 (CH, C4), 40.9 (CH_2, C8), 50.5 (CH_2, C6), 59.7 (CH_2, C2′), 100.4 (CH_2, C15), 106.1 (C, C3), 107.5 (CH, C13), 108.5 (CH, C17), 112.1 (C, C4a), 120.9 (CH, C12), 141.1 (C, C11), 142.9 (C, C14), 145.4 (C, C16), 146.9 (C, C8a), 147.6 (C, C2), 167.3 (C, C1), 195.4 (C, C5). ESI(+)-MS (**Fig. S60**): *m/z* 406 (100%, $[M+Na]^+$), *m/z* 384 (100%, $[M+H]^+$), *m/z* 262 (70%, $[M+H-C_7H_6O_2]^+$).

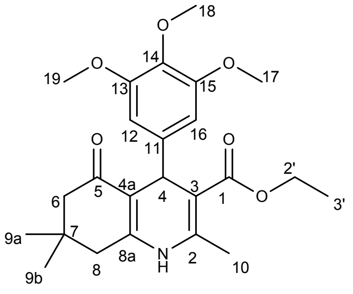

13

(±)-ethyl 2,7,7-trimethyl-5-oxo-4-(3,4,5-trimethoxyphenyl)-1,4,5,6,7,8-hexahydroquinoline-3-carboxylate (**13**). White powder, 34% yield. Purity (HPLC): > 95% (**Fig. S61**). ^1H NMR (400 MHz, *$CDCl_3$*, **Fig. S62**): *δ* 0.97 (3H, *s*, H9b), 1.06 (3H, *s*, H9a), 1.23 (3H, *t*, *J* = 6.8 Hz, H3′), 2.21 (3H, *s*, H10), 2.34 (4H, *s*, H6-H8), 3.77 (9H, *s*, H17=H19 and H18), 4.09 (2H, *q*, *J*=6.8 Hz, H2′), 5.00 (1H, *s*, H4), 6.52 (2H, *s* , H12=H16).^{13}C NMR (100 MHz, *$CDCl_3$*, **Fig. S63** and **S64**): *δ* 14.2

(CH$_3$, C3′), 19.7 (CH$_3$, C10), 27.1 (CH$_3$, C9a), 29.2 (CH$_3$, C9b), 32.9 (C, C7), 36.7 (CH, C4), 41.2 (CH$_2$, C8), 50.9 (CH$_2$, C6), 56.1 (CH$_3$, C19=C17), 59.9 (CH$_2$, C2′), 60.5 (CH$_3$, C18), 104.2 (C, C3), 105.1 (CH, C12=C16), 111.9 (C, C4a), 136.3 (C, C14) , 142.98 (C, C11), 143.4 (C, C13=C15), 148.4 (C, C8a), 152.7 (C, C2), 167.5 (C, C1), 195.8 (C, C5). ESI(+)-MS (**Fig. S65**): *m/z* 452 (55%, $[M+Na]^+$), *m/z* 430 (190%, $[M+H]^+$), *m/z* 262 (100%, $[M+H-C_9H_{12}O_3]^+$).

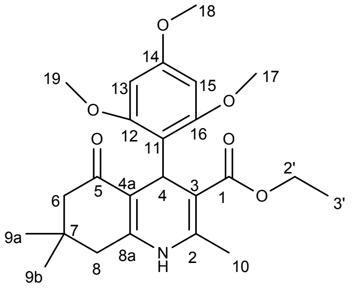

14

(±)-ethyl 2,7,7-trimethyl-5-oxo-4-(2,4,6-trimethoxyphenyl)-1,4,5,6,7,8-hexahydroquinoline-3-carboxylate (**14**). White powder, 39% yield. Purity (HPLC): > 95% (**Fig. S66**). ^{1}H NMR (400 MHz, *CDCl$_3$*, **Fig. S67**): *δ* 0.90 (3H, s, H9b), 1.03 (3H, *s*, H9a), 1.24 (3H, *t*, *J* = 7.1 Hz, H3′), 2.01-2.19 (4H, *m*, H6 and H8), 2.25 (3H, *s*, H10), 3.74 (3H, *s*, H18), 3.79 (6H, *s*, H17=H19), 3.98 (2H, *q*, *J*= 7.1 Hz, H2′), 5.53 (1H, *s*, H4), 6.05 (2H, *s*, H13=H15).^{13}C NMR (100 MHz, *CDCl$_3$*, **Fig. S68** and **S69**): *δ* 14.1 (CH$_3$, C3′), 18.6 (CH$_3$, C10), 26.9 (CH$_3$, C9a), 29.67 (CH$_3$, C9b), 32.6 (C, C7), 35.4 (CH, C4), 41.8 (CH$_2$, C8) 51.1 (CH$_2$, C6), 55.9 (CH$_3$, C17=C19), 58.6 (CH$_3$, C18), 59.6 (CH$_2$, C2′), 90.7 (C, C3), 104.4 (C, C11) , 110.8 (CH, C13=C15), 115.9 (C, C4a), 143.1 (C, C8a), 148.2 (C, C2), 148.6 (C, C12=C16), 159.3 (C, C14), 168.7 (C1′), 195.6 (C, C5). ESI(+)-MS (**Fig. S70**): *m/z* 468 (12%, $[M+K]^+$), *m/z* 452 (50%, $[M+Na]^+$), *m/z* 430 (25%, $[M+H]^+$), *m/z* 262 (100%, $[M+H-C_9H_{12}O_3]^+$).

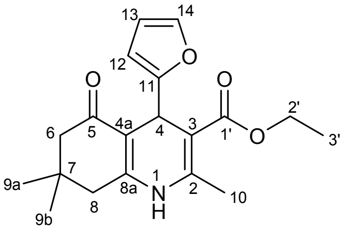

15

(±)-ethyl 4-(furan-2-yl)-2,7,7-trimethyl-5-oxo-1,4,5,6,7,8-hexahydroquinoline-3-carboxylate (**15**). White powder, 30% yield. Purity (HPLC): > 95% (**Fig. S71**). ^{1}H NMR (400 MHz, *CDCl$_3$*, **Fig. S72**): *δ* 1.06 (3H, *s*, H9b), 1.08 (3H, *s*, H9a), 1.21 (3H, *t*, *J*=7.2 Hz, H3′), 2.19-2.34 (4H, *m*, H6-H8), 2.36 (3H, *s*, H10), 4.12 (2H, *q*, *J*= 7.1 Hz, H2′), 5.43 (1H, s, H4), 6.02 (1H, *d*, *J*= 3.2 Hz, H12), 6.21 (1H, *t*, *J*=6.9 Hz, H13), 7.18 (1H, *d*, *J*=3.1 Hz, H14).^{13}C NMR (100 MHz, *CDCl$_3$*, **Fig. S73** and **S74**): *δ* 14.2 (CH$_3$, C3′), 19.4 (CH$_3$, C10), 27.5 (CH$_3$, C9a), 29.5 (CH$_3$, C9b), 32.8 (C, C7), 37.0 (CH, C4), 41.2 (CH$_2$, C8), 50.4 (CH$_2$, C6), 60.2 (CH$_2$, C2′), 106.3 (C, C3), 111.4 (C, C4a), 123.6 (CH, C12), 125.4 (CH, C14), 127.1 (CH, C13), 140.7 (C, C11), 148.4 (C, C8a), 157.4 (C, C2), 166.8 (C, C1′), 195.4 (C, C5). ESI(+)-MS (**Fig. S75**): *m/z* 368 (5%, $[M+K]^+$), *m/z* 352 (90%, $[M+Na]^+$), *m/z* 330 (12%, $[M+H]^+$), *m/z* 262 (100%, $[M+H-C_4H_4O]^+$).

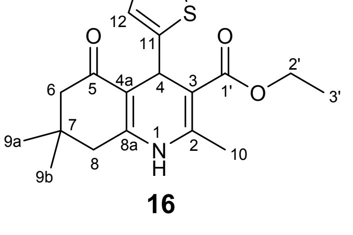

16

(±)-ethyl 2,7,7-trimethyl-5-oxo-4-(thiophen-2-yl)-1,4,5,6,7,8-hexahydroquinoline-3-carboxylate (**16**). Beige powder, 38% yield. Purity (HPLC): > 95% (**Fig. S76**). ^{1}H NMR (400 MHz, *CDCl$_3$*, **Fig. S77**): *δ* 1.03 (3H, *s*, H9b), 1.10 (3H, *s*, H9a), 1.25 (3H, *t*, *J* = 7.1 Hz, H3′), 2.21-2.36 (4H, *m*, H6-H8), 2.38 (3H, *s*, H10), 4.14 (2H, *q*, *J*= 7.1 Hz, H2′), 5.41 (1H, *s*, H4), 6.81-6.84 (2H, *m*, H13 and H12), 7.03 (1H, *d*, *J*=4.9 Hz, H14).^{13}C NMR (100 MHz, *CDCl$_3$*, **Fig. S78** and **S79**): *δ* 14.2 (CH$_3$, C3′),

19.4 (CH_3, C10), 27.2 (CH_3, C9a), 29.4 (CH_3, C9b), 32.7 (C, C7), 36.9 (CH, C4), 41.1 (CH_2, C8), 50.5 (CH_2, C6), 60.2 (CH_2, C2′), 106.3 (C, C3), 111.3 (C, C4a), 123.5 (CH, C12), 125.4 (CH, C14), 127.2 (CH, C13), 140.7 (C, C11), 148.4 (C, C8a), 157.6 (C, C2), 166.7 (C, C1), 195.3 (C, C5). ESI(+)-MS (**Fig. S80**): *m*/*z* 368 (65%, $[M+Na]^+$), *m*/*z* 346 (30%, $[M+H]^+$), *m*/*z* 262 (100%, $[M+H-C_4H_4S]^+$).

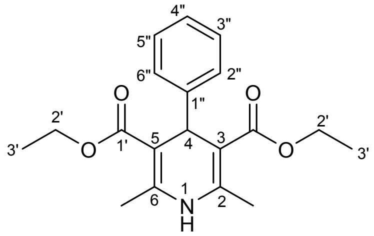

17

Diethyl 2,6-dimethyl-4-phenyl-1,4-dihydropyridine-3,5-dicarboxylate (**17**). Yellow powder, 15% yield. Purity (HPLC): > 95% (**Fig. S81**). ^{1}H NMR (400 MHz, *CDCl$_3$*, **Fig. S82**): 1.24 (6H, *t*, $J_{3',2'}$ = 7.1 Hz, H3′), 2.33 (6H, *s*, 2=6-CH_3), 4.08 (4H, *q*, $J_{2',3'}$ = 7.1 Hz, H2′), 4.98 (1H, *s*, H4), 7.15 – 7.32 (5H, *m*, H2″=H6″, H3″=H5″ and H4″). ^{13}C NMR (100 MHz, *CDCl$_3$*, **Fig. S83** and **S84**): δ 14.1 (CH_3, C3′), 19.4 (CH_3, 2=6-CH_3), 39.4 (CH, C4), 59.5 (CH_2, C2′), 104.0 (C, C3=C5), 125.9 (CH, C4″), 127.6 (CH, C2″=C6″), 127.8 (CH, C3″=C5″), 143.6 (C, C1″), 147.6 (C, C2=C6), 167.7 (C, C1). ESI(+)-MS (**Fig. S85**): *m*/*z* 368 (8%, $[M+K]^+$), *m*/*z* 352 (100%, $[M+Na]^+$), *m*/*z* 330 (25%, $[M+H]^+$), *m*/*z* 284 (100%, $[M+H-C_2H_6O]^+$), *m*/*z* 252 (20%, $[M+H-C_6H_6]^+$).

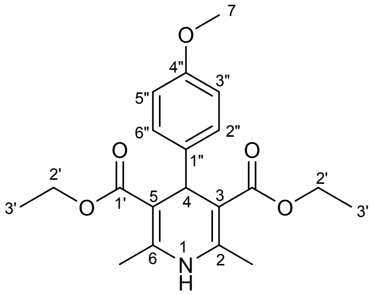

18

D*iethyl 4-(4-methoxyphenyl)-2,6-dimethyl-1,4-dihydropyridine-3,5-dicarboxylate* (**18**). Yellow powder, 17% yield. Purity (HPLC): > 95% (**Fig. S86**). NMR ^{1}H (400 MHz, *CDCl$_3$*, **Fig. S87**): 1.22 (6H, *t*, $J_{3',2'}$ = 7.1 Hz, H3′), 2.32 (6H, *s*, 2=6-CH_3), 3.75 (3H, *s*, H7), 4.08 (4H, *q*, $J_{2',3'}$ = 5.2 Hz, H2′), 4.92 (1H, *s*, H4), 6.75 (2H, *d*, *J* = 7.7 Hz, H3″=H5″), 7.20 (2H, *d*, *J*=7.7 Hz, H2″=H6″).^{13}C NMR (100 MHz, *CDCl$_3$*, **Fig. S88** and **S89**): δ 14.1 (CH_3, C3′), 19.4 (CH_3, 2=6-CH_3), 38.6 (CH, C4), 54.9 (CH_3, C7), 59.5 (CH_2, C2′), 104.2 (C, C3=C5), 113.0 (CH, C3″=C5″), 128.8 (CH, C2″=C6″), 140.1 (C, C1″), 143.3 (C, C2=C6), 157.7 (C, C4″), 167.5 (C, C1′). ESI(+)-MS (**Fig. S90**): *m*/*z* 398 (5%, $[M+K]^+$), *m*/*z* 382 (95%, $[M+Na]^+$), *m*/*z* 360 (12%, $[M+H]^+$), *m*/*z* 252 (100%, $[M+H-C_7H_8O]^+$).

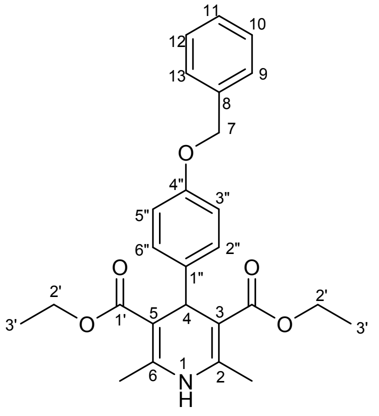

19

Diethyl 4-(4-(benzyloxy)phenyl)-2,6-dimethyl-1,4-dihydropyridine-3,5-dicarboxylate (**19**). Yellow powder, 16% yield. Purity (HPLC): > 95% (**Fig. S91**). NMR ^{1}H (400 MHz, *CDCl$_3$*, **Fig. S92**): 1.22 (6H, *t*, $J_{3',2'}$ = 7.1 Hz, H3′), 2.32 (6H, *s*, 2=6-CH_3), 4.09 (4H, *q*, $J_{2',3'}$ = 7.1 Hz, H2′), 4.93 (1H, *s*, H4), 5.00 (2H, *s* H7), 6.83 (2H, *d*, *J*= 8.7 Hz, H3″=H5″), 7.20 (2H, *d*, *J*= 8.7 Hz, H2″=H6″), 7.31-7.42 (5H, *m*, H8, H9=H13, and H10=H12). ^{13}C NMR (400 MHz, *CDCl$_3$*, **Fig. S93** and **S94**): δ 14.6 (CH_3, C3′), 19.4 (CH_3, 2=6-CH_3), 38.7 (CH, C4), 59.9 (CH_2, C2′), 70.2 (CH_2, C7), 104.6 (C, C3=C5), 114.2 (CH, C3″=C5″), 127.6 (CH, C9=C13), 127.9 (CH, C11), 128.7 (CH, C10=C12), 129.2 (CH, C2″=C6″), 137.6 (C, C8),

140.5 (C, C1″), 143.4 (C, C2=C6), 157.2 (C, C4″), 167.8 (C, C1′). ESI(+)-MS (**Fig. S95**): *m/z* 474 (13%, $[M+K]^+$), *m/z* 458 (100%, $[M+Na]^+$), *m/z* 436 (10%, $[M+H]^+$), *m/z* 252 (72%, $[M+H-C_{13}H_{12}O]^+$).

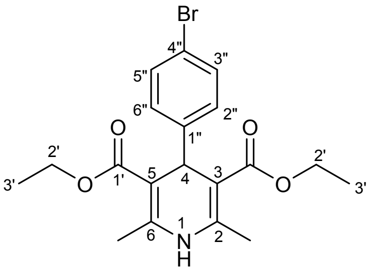

20

Diethyl 4-(4-bromophenyl)-2,6-dimethyl-1,4-dihydropyridine-3,5-dicarboxylate (**20**). Yellow powder, 19% yield. Purity (HPLC): > 95% (**Fig. S96**). ^{1}H NMR (400 MHz, *CDCl$_3$*, **Fig. S97**): 1.21 (6H, *t*, $J_{3',2'}$ = 7.1 Hz, H3′), 1.60 (3H, *J*=7.1 Hz, H3′), 2.32 (6H, *s*, 2=6-CH_3), 4.08 (4H, *q*, $J_{2',3'}$ = 4.3 Hz, H2′), 4.93 (1H, *s*, H4), 7.16 (2H, *d*, *J*=8.4 Hz, H2″=H6″), 7.33 (2H, *d*, *J*=8.4 Hz, H3″=H5″) ^{13}C NMR (100 MHz, *CDCl$_3$*, **Fig. S98** and **S99**): *δ* 14.9 (CH_3, C3′), 20.1 (CH_3, 2=6-CH_3), 39.6 (CH, C4), 60.2 (CH_2, C2′), 104.5 (C, C3=C5), 120.2 (C, C4″), 130.2 (CH, C2″=C6″), 131.2 (CH, C3″=C5″), 144.1 (C, C1″), 146.9 (C, C2=C6), 167.8 (C, C1′). ESI(+)-MS (**Fig. S100**): *m/z* 430/432 (100%, $[M+Na]^+$), *m/z* 408/410 (10%, $[M+H]^+$), *m/z* 362 (60%, $[M+H-C_2H_6O]+$), *m/z* 252(15%, $[M+H-C_6H_5Br]^+$).

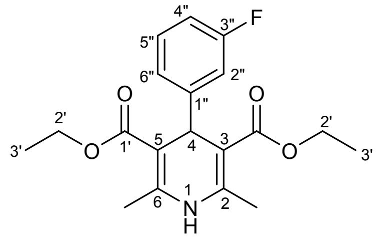

21

Diethyl 4-(3-fluorophenyl)-2,6-dimethyl-1,4-dihydropyridine-3,5-dicarboxylate (**21**). Yellow powder, 20% yield. Purity (HPLC): > 95% (**Fig. S101**). ^{1}H NMR (400 MHz, *CDCl$_3$*, **Fig. S102**): 1.22 (6H, *t*, $J_{3',2'}$ = 7.1 Hz, H3′), 2.34 (6H, *s*, 2=6-CH_3), 4.09 (4H, *q*, $J_{2',3'}$ = 7.1 Hz, H2′), 4.99 (1H, *s*, H4), 6.81 (1H, *t*, *J*=7.2 Hz, H6″), 6.98 (1H, *d*, *J*= 8.2 Hz, H4″), 7.07 (1H, *d*, *J*= 6.9 Hz, H2″), 7.16 (1H, *dd*, *J*=7.4, and 8.2 Hz, H5″). ^{13}C NMR (400 MHz, *CDCl$_3$*, **Fig. S103** and **S104**): *δ* 14.3 (CH_3, C3′), 19.8 (CH_3, 2=6-CH_3), 39.7 (CH, C4), 59.9 (CH_2, C2′), 103.6 (C, C3=C5), 114.0 (CH, C4″), 115.1 (CH, C2″), 123.8 (CH, C6″), 129.3 (CH, C5″), 144.4 (C, C1″), 157.9 (C, C2=C6), 163.9 (C, C3′), 167.5 (C, C1′). ESI(+)-MS (**Fig. S105**): *m/z* 386 (20%, $[M+K]^+$), *m/z* 370 (100%, $[M+Na]^+$), *m/z* 348 (60%, $[M+H]^+$), *m/z* 302 (100%, $[M+H-C_2H_6O]^+$), *m/z* 274 (25%, $[M+H-C_2H_6O-CO]^+$), *m/z* 252 (20%, $[M+H-C_6H_5F)]^+$).

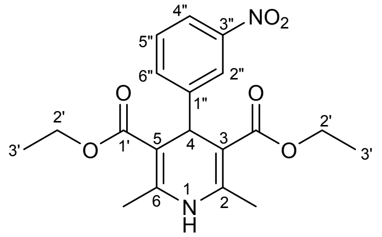

22

(±)-diethyl 2,6-dimethyl-4-(3″-nitrophenyl)-1,4-dihydropyridine-3,5-dicarboxylate (**22**). Yellow powder, 16% yield. Purity (HPLC): > 95% (**Fig. S106**). ^{1}H NMR (400 MHz, *CDCl$_3$*, **Fig. S107**): 1.22 (6H, *t*, $J_{3',2'}$ = 7.1 Hz, H3′), 2.36 (6H, *s*, 2=6-CH_3), 4.08 (4H, *q*, $J_{2',3'}$ = 7.1 Hz, H2′), 5.08 (1H, *s*, H4), 7.37 (1H, *t*, *J* = 7.9 Hz, H5″), 7.62 (1H, *d*, *J* = 7.7 Hz, H6″), 8.00 (1H, *d*, *J*= 8.2 Hz, H4″), 8.12 (1H, *t*, *J* = 1.8 Hz, H2″). ^{13}C NMR (100 MHz, *CDCl$_3$*, **Fig. S108** and **S109**): *δ* 14.5 (CH_3, C3′), 19.7 (CH_3, 2=6-CH_3), 40.2 (CH, C4), 60.0 (CH_2, C2′), 103.4 (C, C3=C5), 121.3 (CH, C5″), 123.1 (CH, C6″), 128.7 (CH, C4″), 134.54 (CH, C2″), 144.6 (C, C3″), 148.3 (C, C1″), 149.8 (C, C2=C6), 167.1 (C, C1′). ESI(+)-MS (**Fig. S110**): *m/z* 413 (3%, $[M+K]^+$), *m/z* 397 (100%, $[M+Na]^+$), *m/z* 375 (10%, $[M+H]^+$), *m/z* 252 (50%, $[M+H-C_6H_5NO_2]^+$).

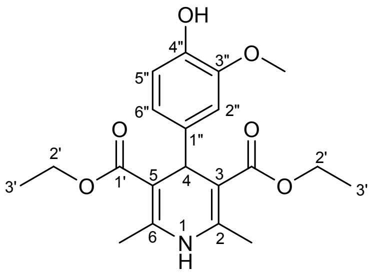
23

Diethyl 4-(4″-hydroxy-3″-methoxyphenyl)-2,6-dimethyl-1,4-dihydropyridine-3,5-dicarboxylate (**23**). Yellow powder, 16% yield. Purity (HPLC): > 95% (**Fig. S111**). NMR ^{1}H (400 MHz, *CDCl$_3$*, **Fig. S112**): 1.22 (6H, *t*, $J_{3',2'}$ = 7.1 Hz, H3′), 2.33 (6H, *s*, 2=6-CH_3), 3.84 (3H, *s*, 3-OCH_3), 4.11 (4H, *q*, $J_{2',3'}$ = 7.1 Hz, H2′), 4.92 (1H, *s*, H4), 6.75 (2H, *m*, H2= H6), 6.85 (1H, s, H2″).^{13}C (100 MHz, *CDCl$_3$*, **Fig. S113**and **S114**): *δ* 14.4 (CH_3, C3′), 19.8 (CH_3, 2=6-CH_3), 39.2 (CH, C4), 55.9 (CH_3, 3″-OCH_3), 59.7 (CH_2, C2′), 104.6 (C, C3=C5), 111.1 (CH, C5″), 113.9 (CH, C2″), 120.6 (CH, C6″), 140.2 (C, C1″), 143.6 (C, C4″), 144.0 (C3″), 145.9 (C, C2=C6), 167.8 (C1′). ESI(+)-MS (**Fig. S115**): *m/z* 414 (8%, $[M+K]^+$), *m/z* 398 (80%, $[M+Na]^+$), *m/z* 376 (15%, $[M+H]^+$), *m/z* 252 (100%, $[M+H-C_7H_8O_2]^+$)

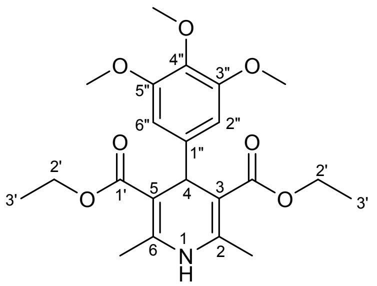
24

Diethyl 2,6-dimethyl-4-(3″,4″,5″-trimethoxyphenyl)-1,4-dihydropyridine-3,5-dicarboxylate (**24**). Yellowish powder, 20% yield. Purity (HPLC): > 95% (**Fig. S116**). NMR ^{1}H (400 MHz, *CDCl$_3$*, **Fig. S117**): 1.25 (6H, *t*, $J_{3',2'}$ = 7.1 Hz, H3′), 2.34 (6H, s, 2=6-CH_3), 3.79 (9H, s, 3=5 and 4-OMe), 4.12 (4H, *q*, $J_{2',3'}$ = 7.1 Hz, H2′), 4.96 (1H, *s*, H7), 6.51 (2H, *s*, H2″=H6″).^{13}C NMR (100 MHz, *CDCl$_3$*, **Fig. S118** and **S119**): *δ* 14.2 (CH_3, C3′), 19.5 (CH_3, 2=6-CH_3), 39.4 (CH, C4), 55.7 (CH_3, 3″=5″-OCH_3), 59.6 (CH_3, 4″-OCH_3), 60.5 (CH_2, C2′), 103.9 (C, C3=C5), 104.8 (CH, C2″=C6″), 136.3 (C, C1″), 143.2 (C, C4″), 143.4 (C, C2=C6), 152.4 (C, C3″=C5″), 167.5 (C, C1′). ESI(+)-MS (**Fig. S120**): *m/z* 442 (100%, $[M+Na]^+$), *m/z* 420 (22%, $[M+H]^+$), *m/z* 252 (85%, $[M+H-C_9H_{12}O_3]^+$).

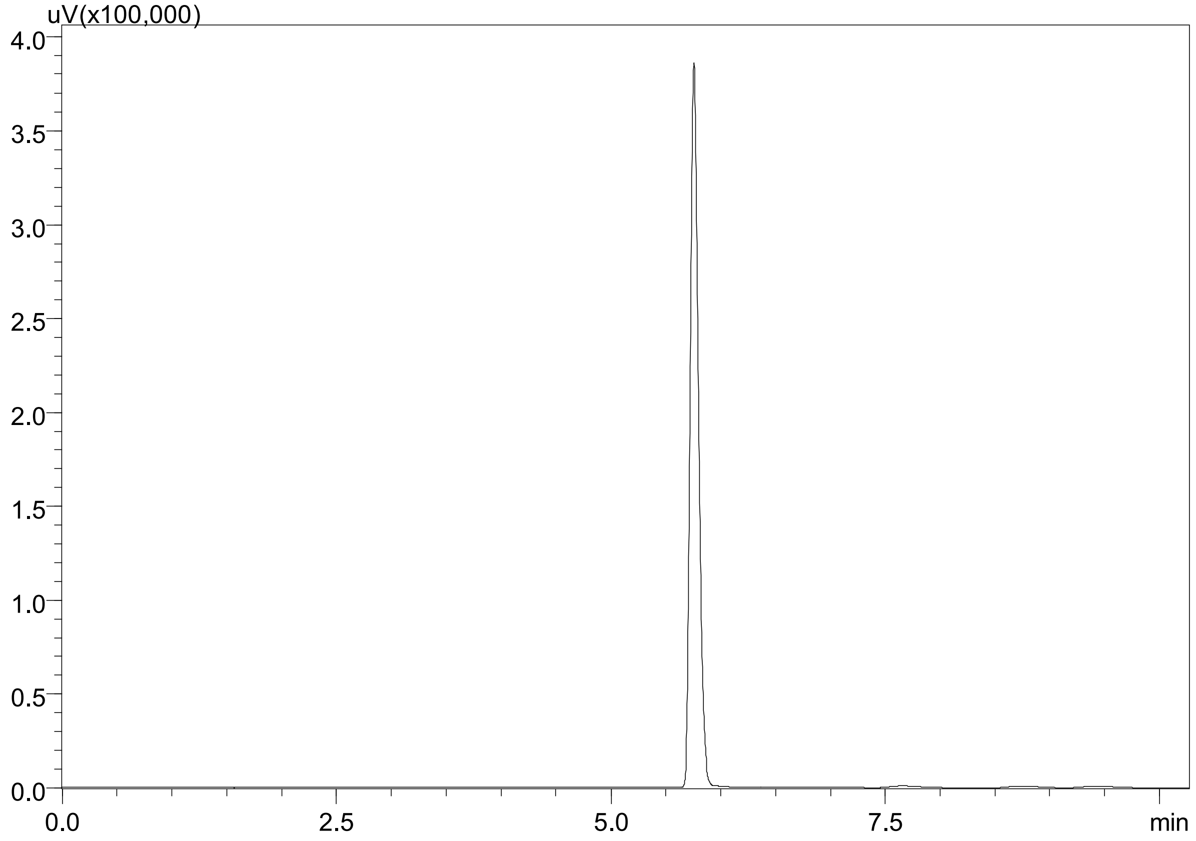

Figure S1: HPLC chromatogram of compound **1**.

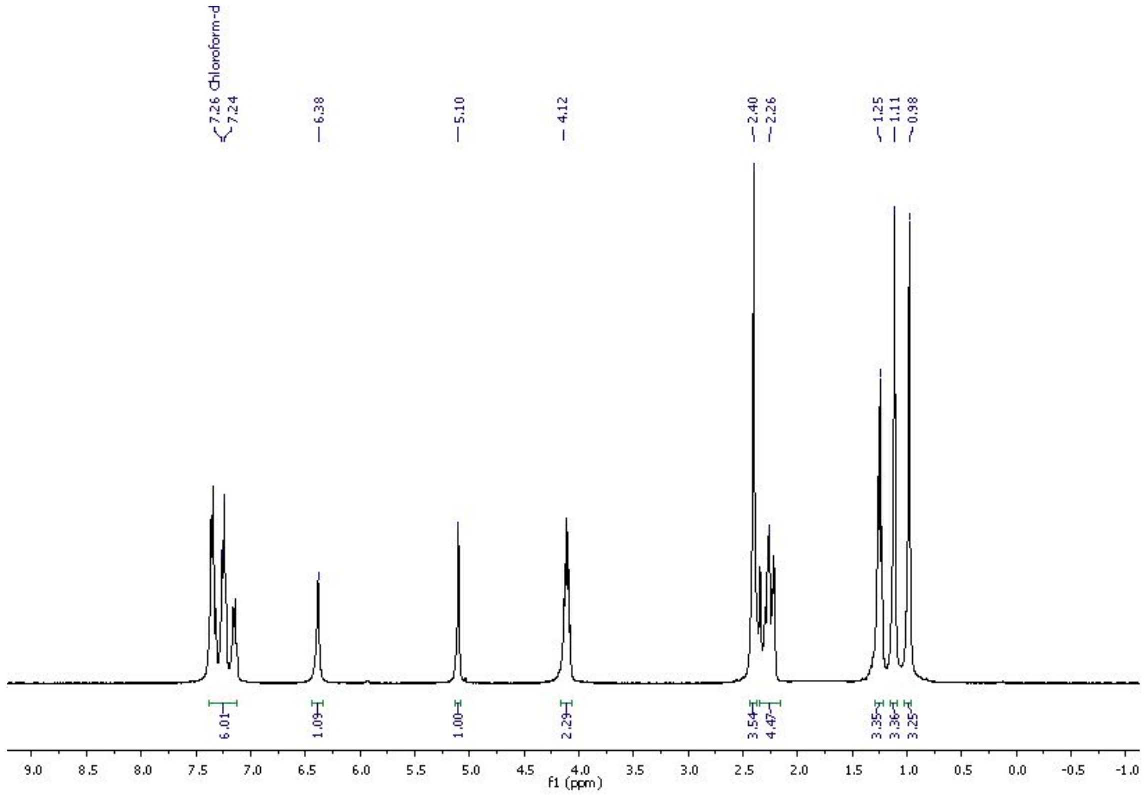

Figure S2.^{1}H NMR spectrum of compound **1** ($CDCl_3$, 400 MHz, TMS).

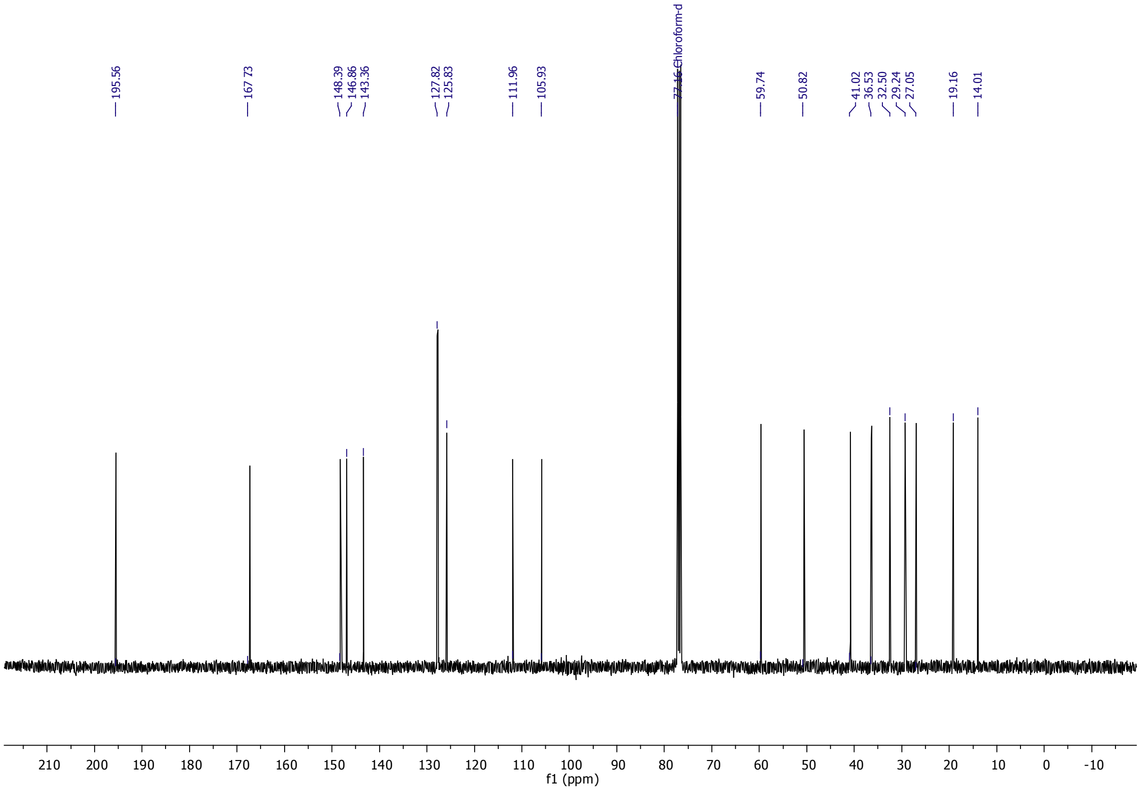

Figure S3.^{13}C NMR spectrum of compound **1** ($CDCl_3$, 100 MHz, TMS).

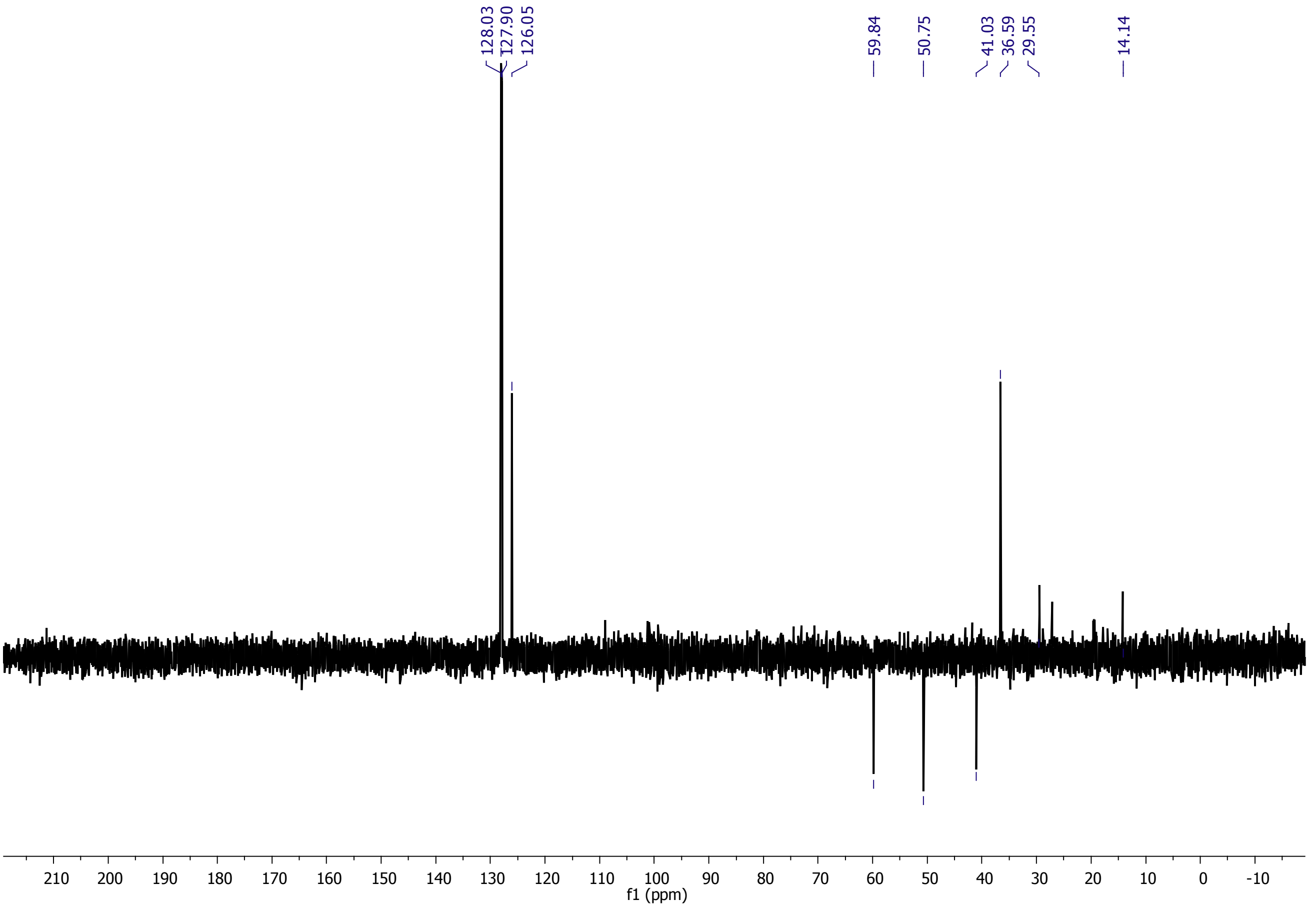

Figure S4. DEPT 135 spectrum of compound **1** ($CDCl_3$, 100 MHz, TMS).

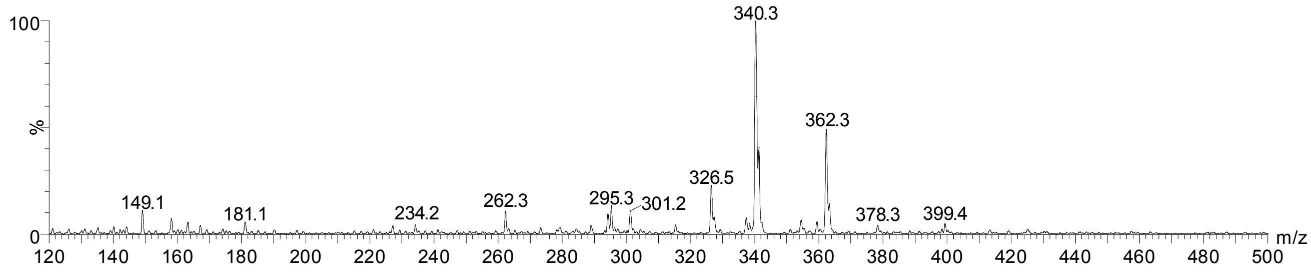

Figure S5. ESI (+) mass spectrum of compound **1**.

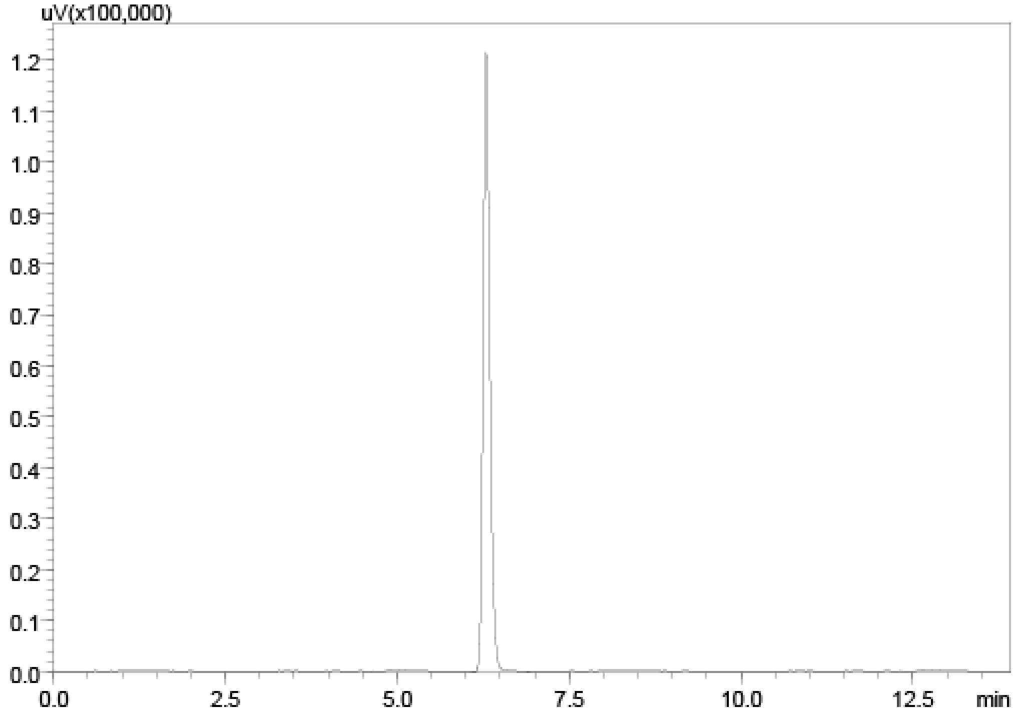

Figure S6: HPLC chromatogram of compound **2**.

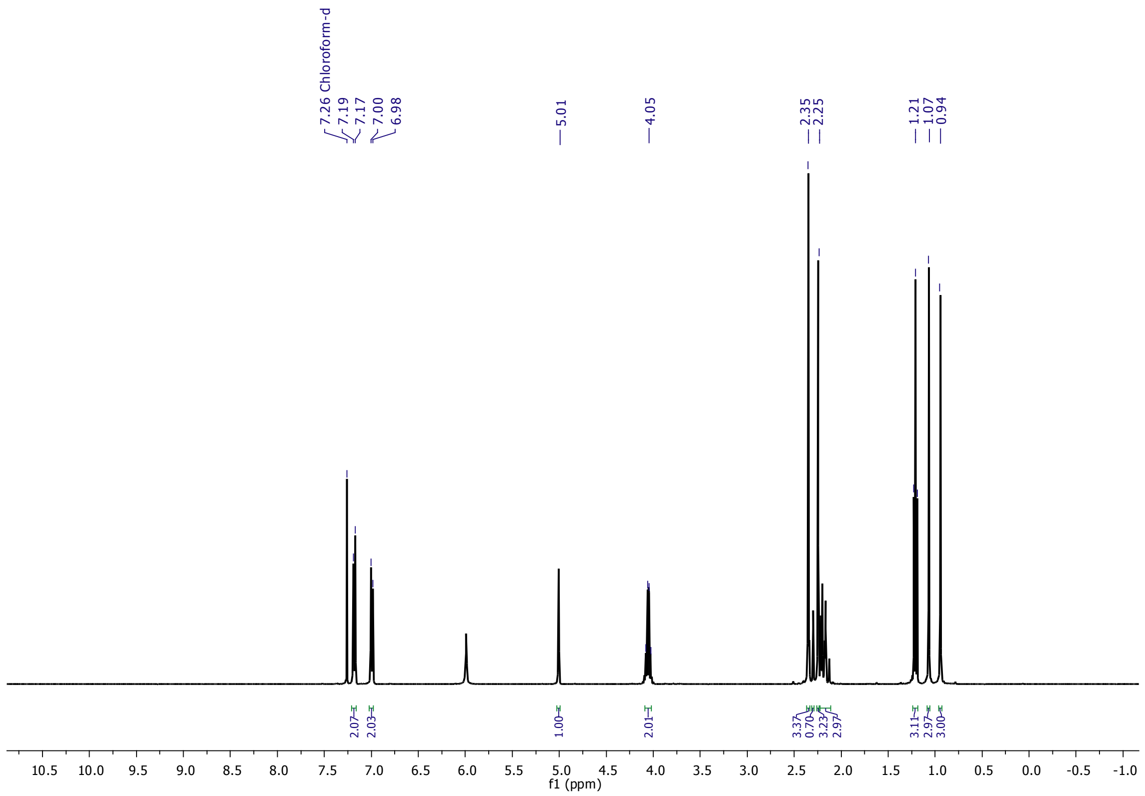

Figure S7.^{1}H NMR spectrum of compound **2** ($CDCl_3$, 400 MHz, TMS).

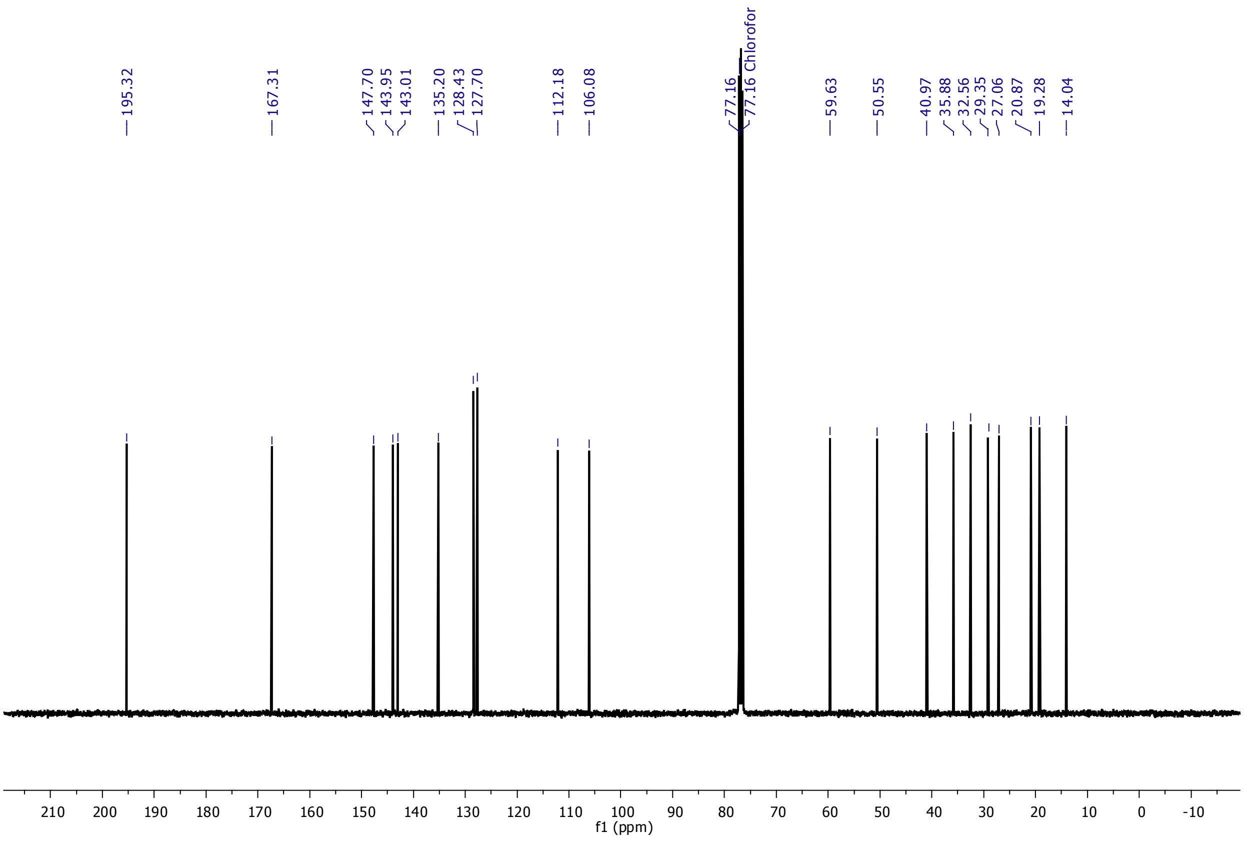

Figure S8.^{13}C NMR spectrum of compound **2** ($CDCl_3$, 100 MHz, TMS).

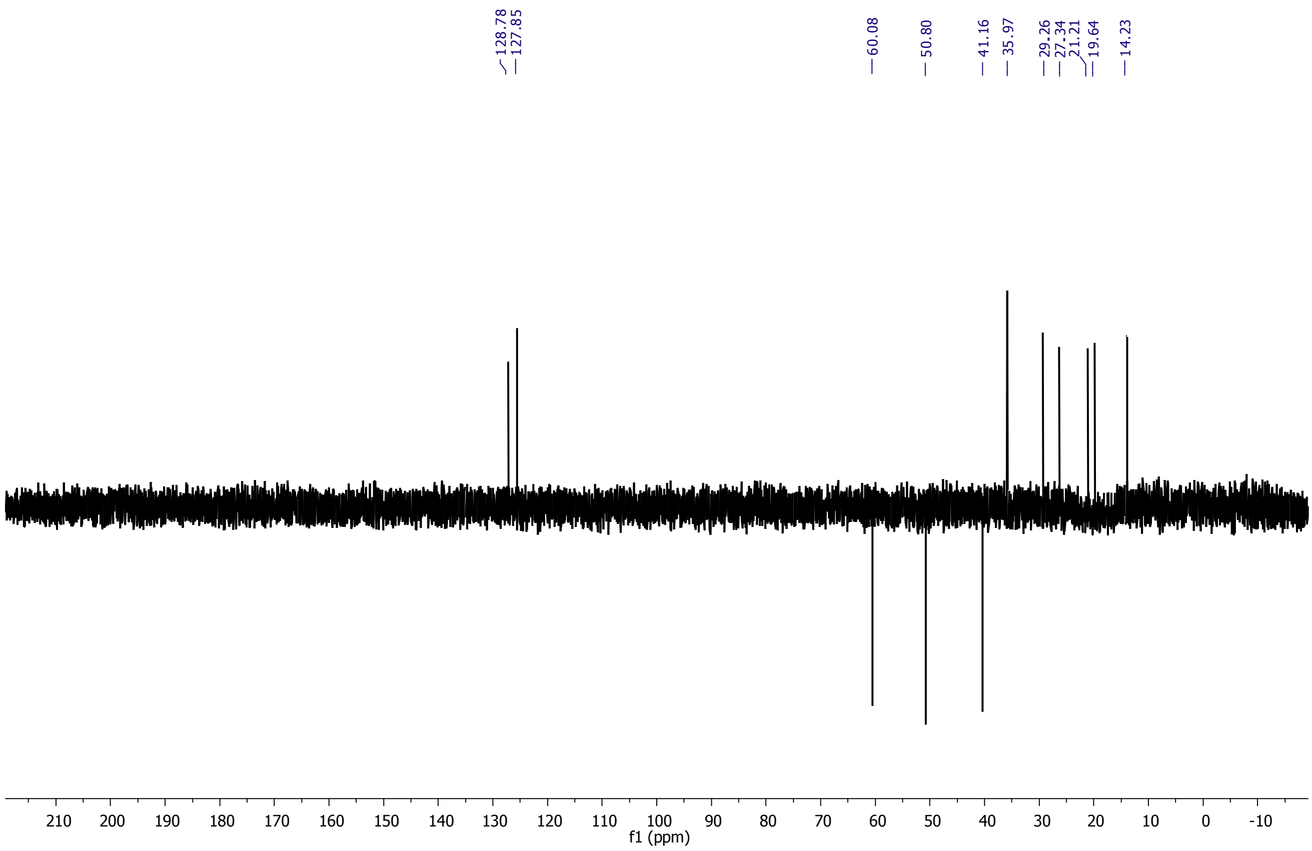

Figure S9.^{13}C NMR spectrum of compound **2** ($CDCl_3$, 100 MHz, TMS).

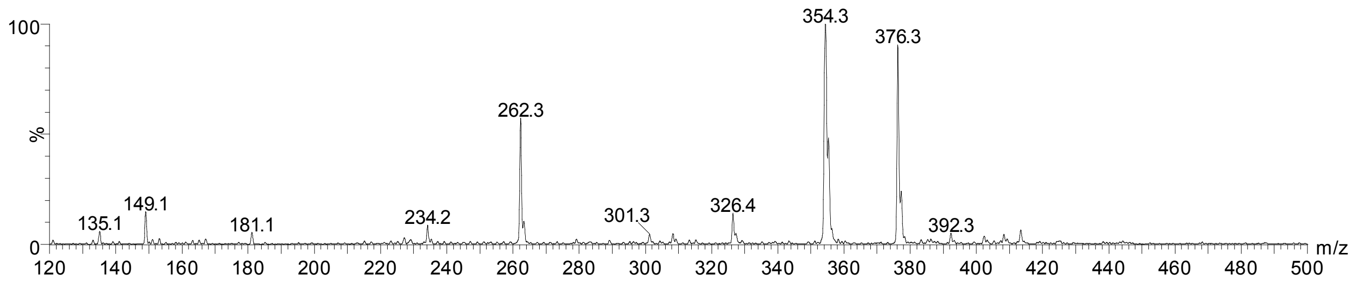

Figure S10. ESI (+) mass spectrum of compound **2**.

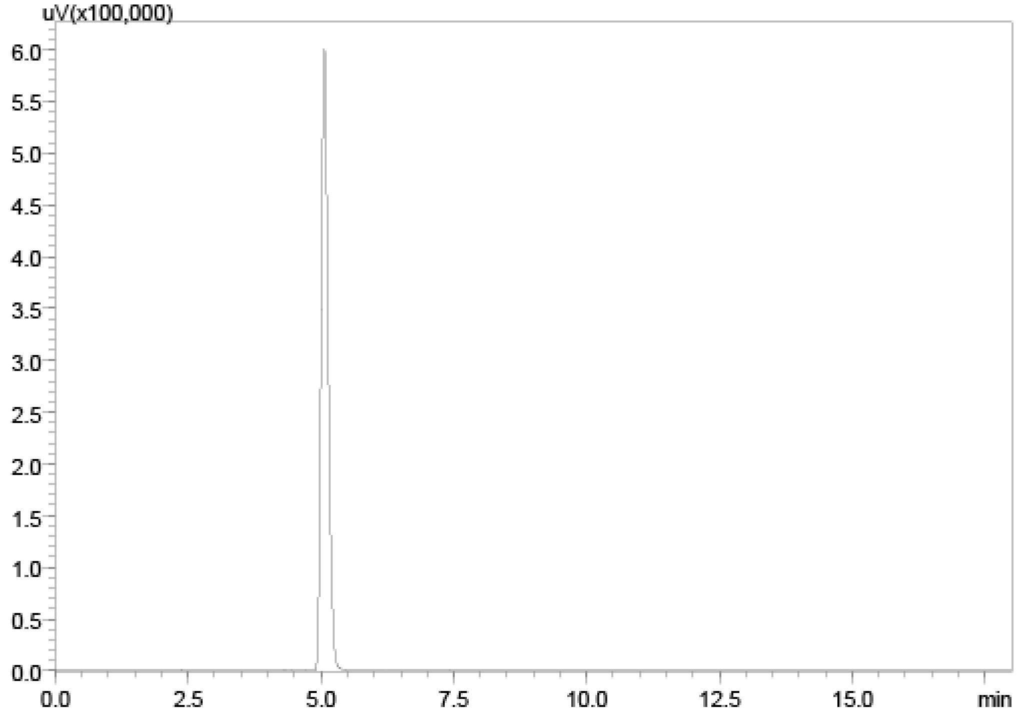

Figure S11: HPLC chromatogram of compound **3**.

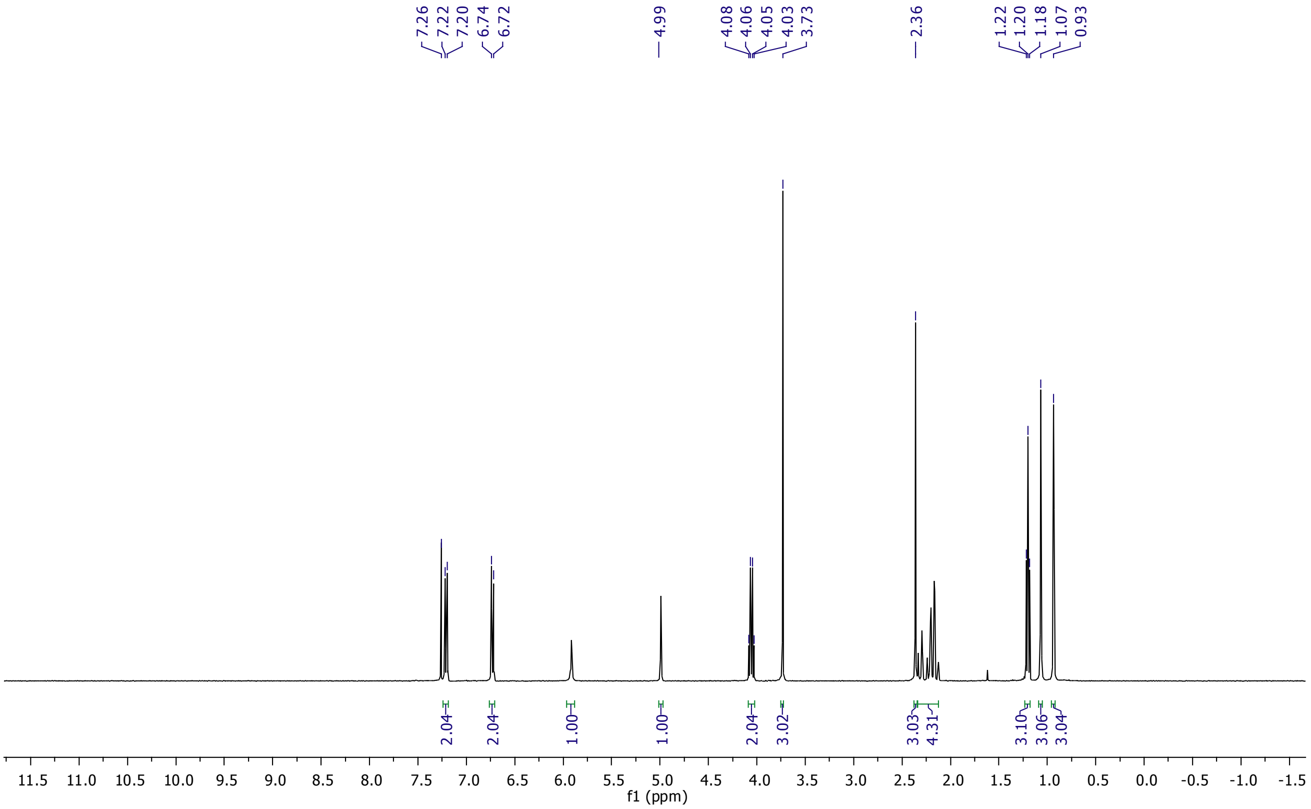

Figure S12.^{1}H NMR spectrum of compound **3** ($CDCl_3$, 400 MHz, TMS).

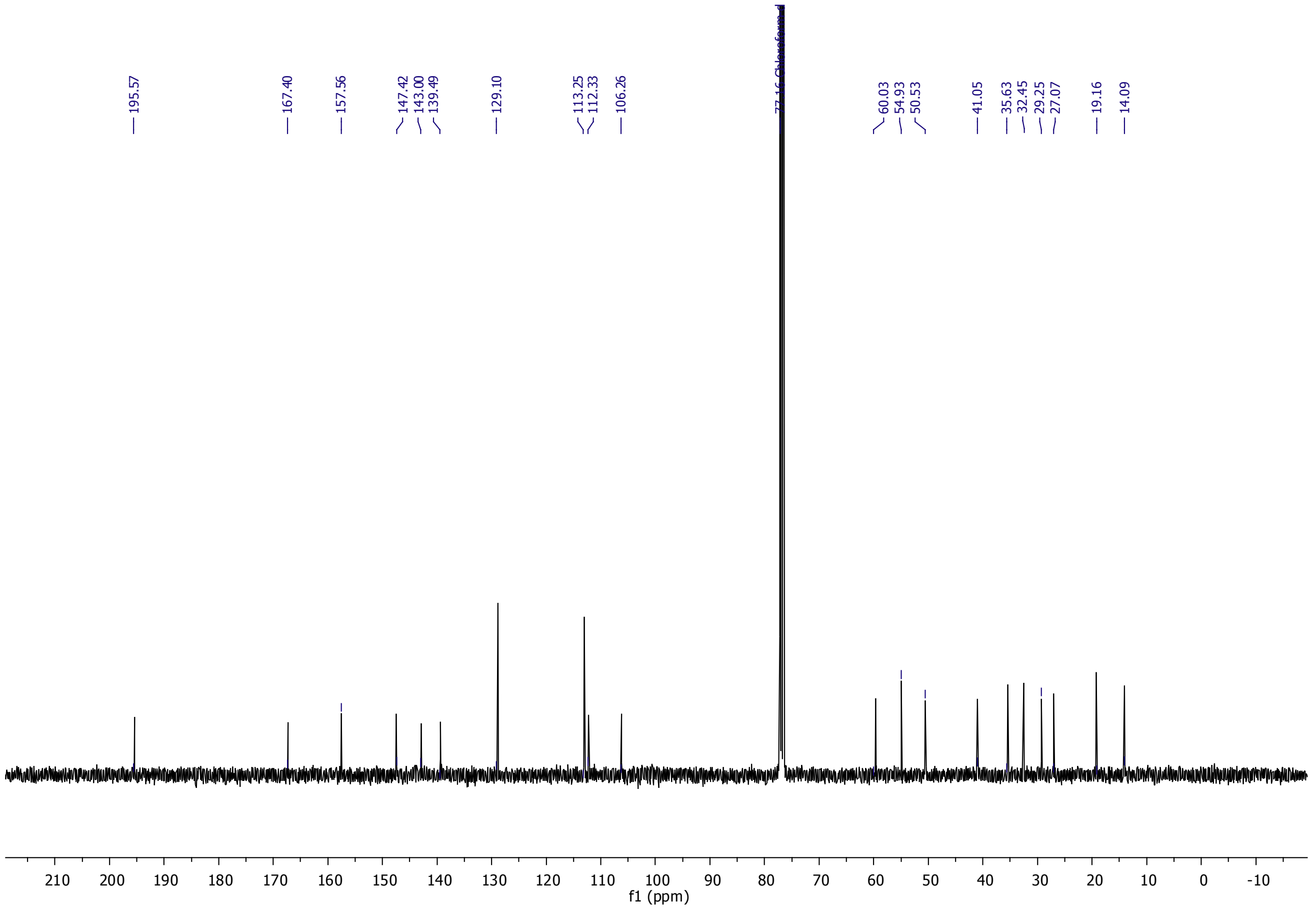

Figure S13.^{13}C NMR spectrum of compound **3** ($CDCl_3$, 100 MHz, TMS).

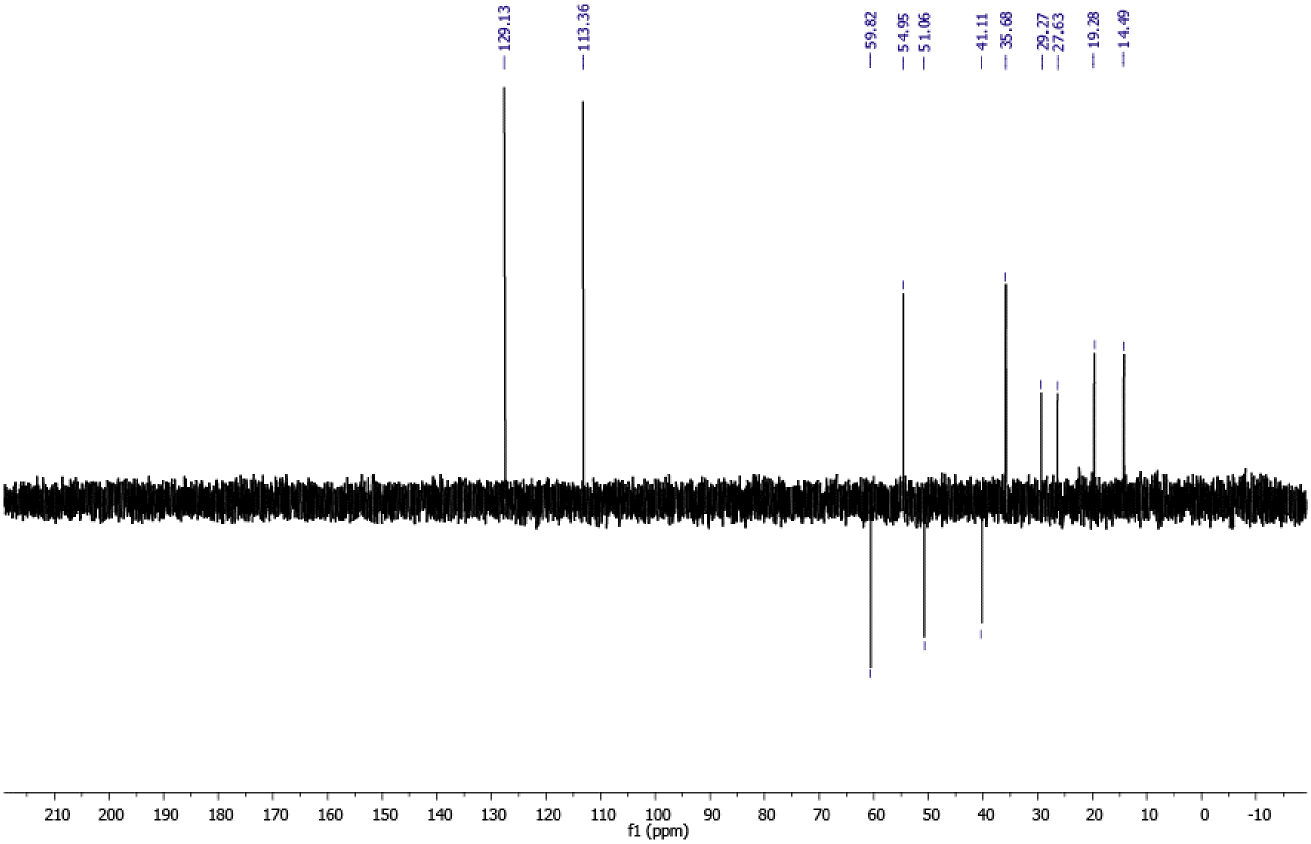

Figure S14. DEPT 135 spectrum of compound **3** ($CDCl_3$, 100 MHz, TMS).

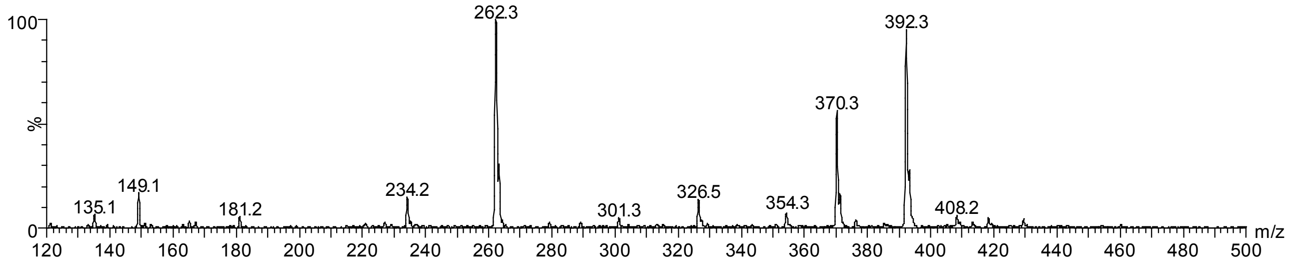

Figure S15. ESI (+) mass spectrum of compound **3**.

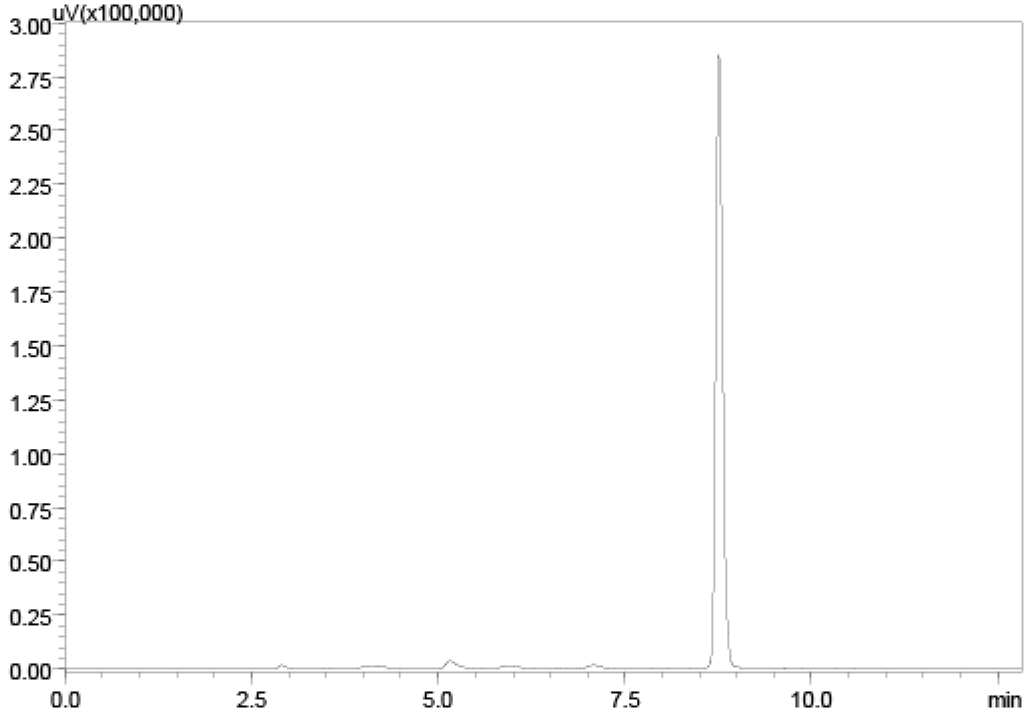

Figure S16: HPLC chromatogram of compound **4**.

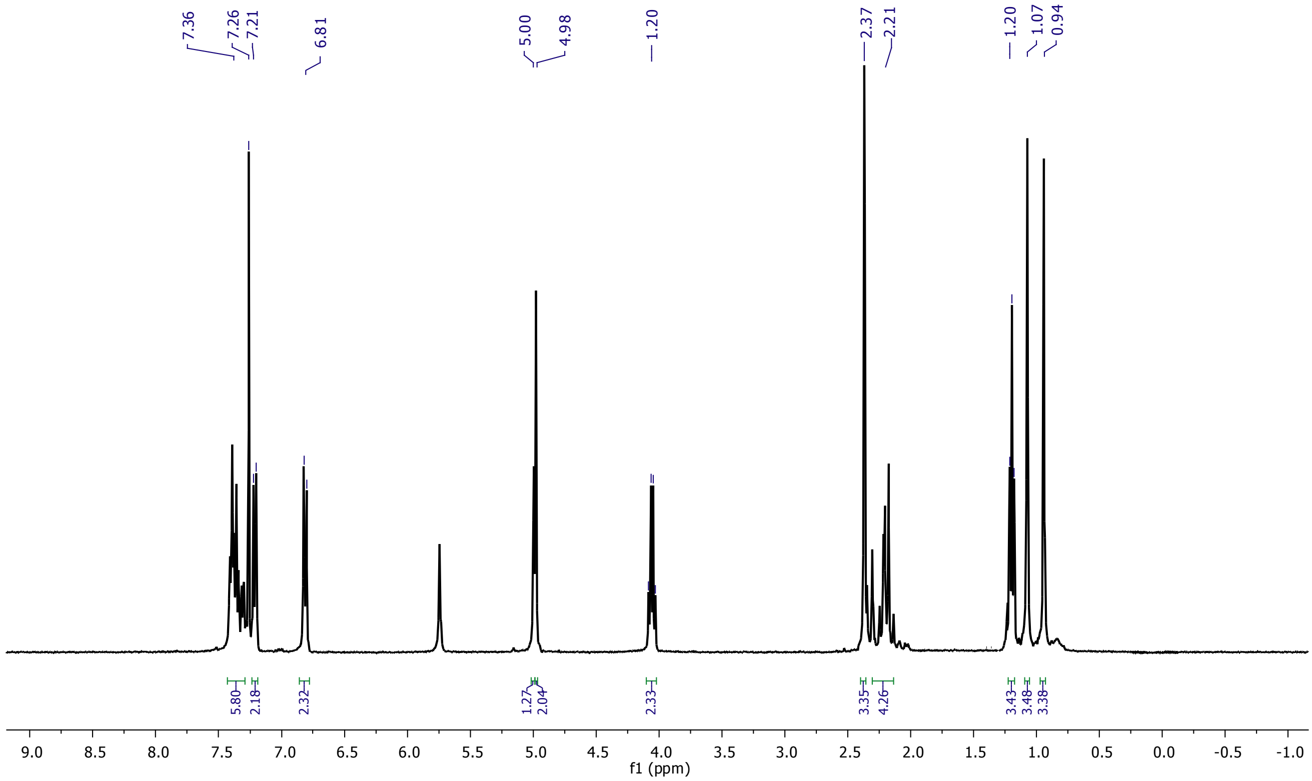

Figure S17.^{1}H NMR spectrum of compound **4** ($CDCl_3$, 400 MHz, TMS).

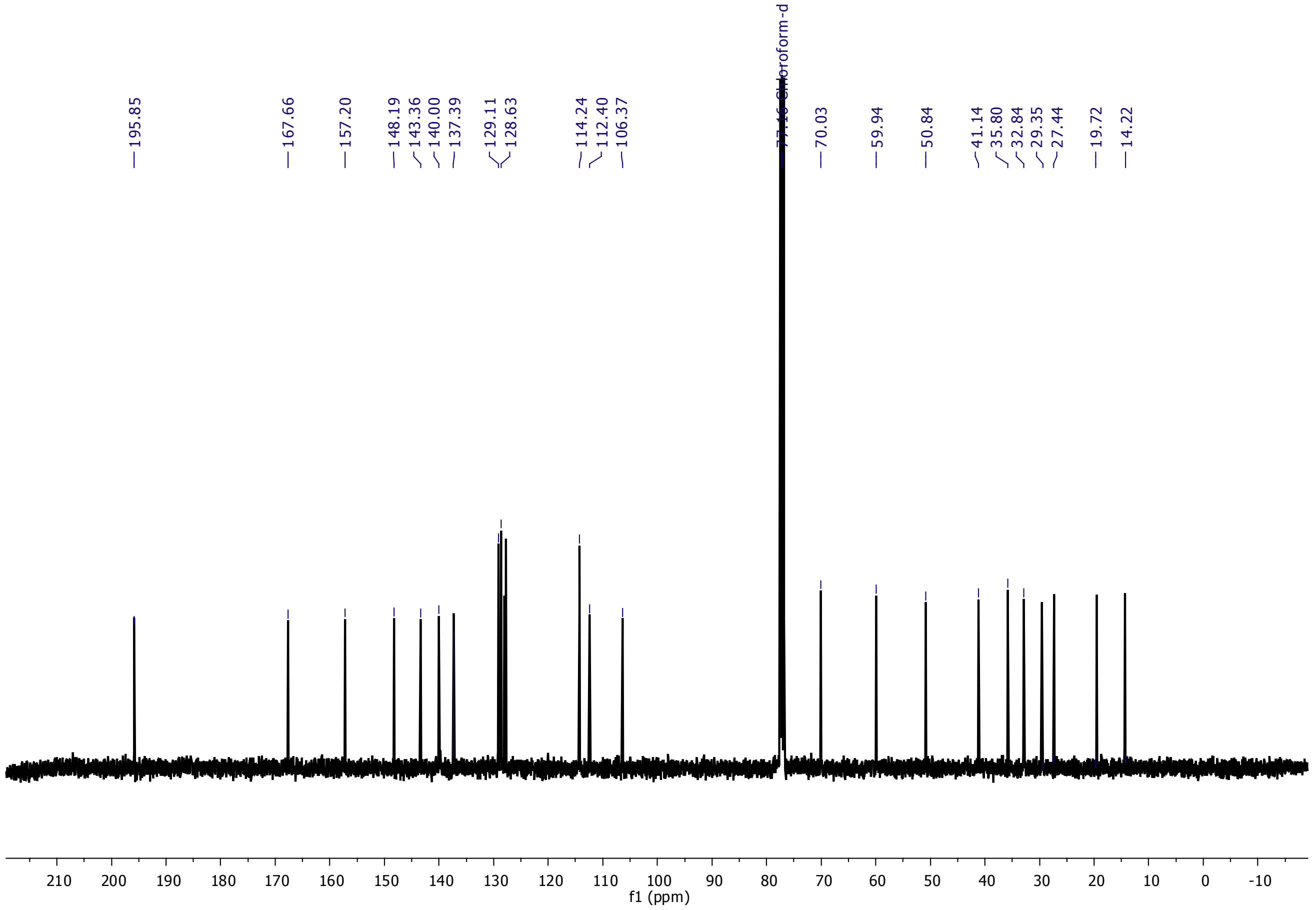

Figure S18.^{13}C NMR spectrum of compound **4** ($CDCl_3$, 100 MHz, TMS).

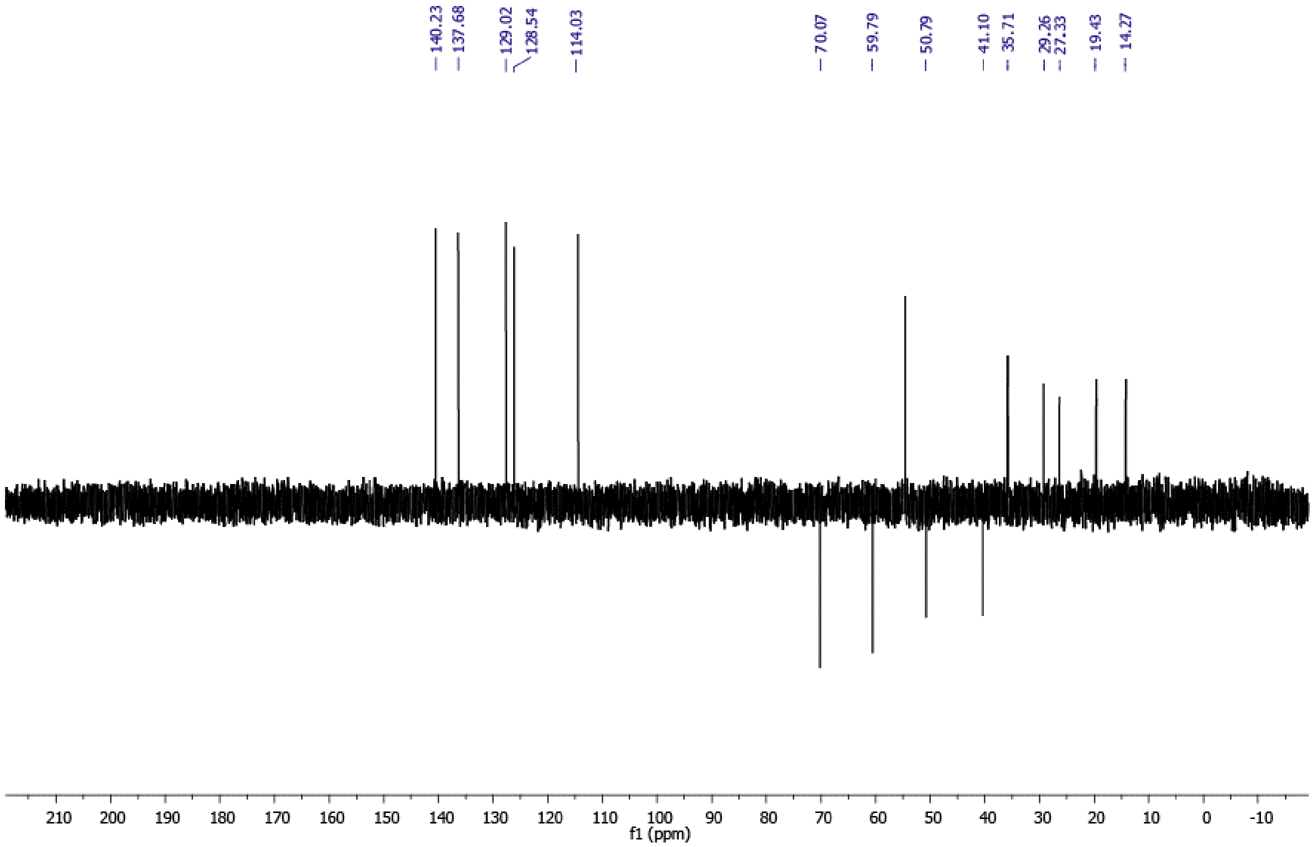

Figure S19. DEPT 135 spectrum of compound **4** ($CDCl_3$, 100 MHz, TMS).

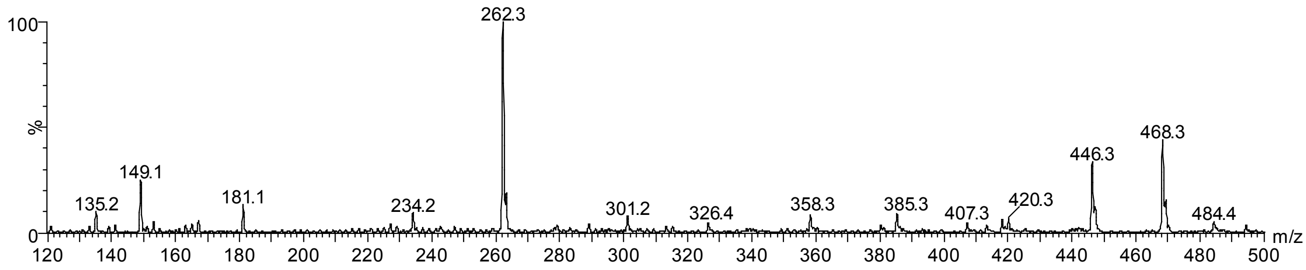

Figure S20. ESI (+) mass spectrum of compound **4**.

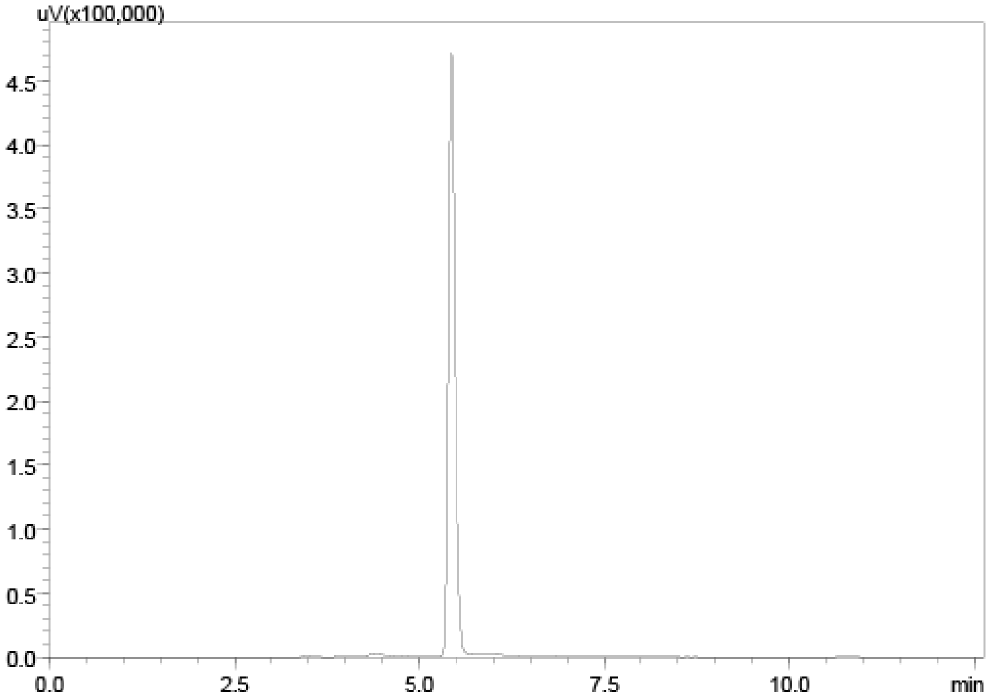

Figure S21: HPLC chromatogram of compound **5**.

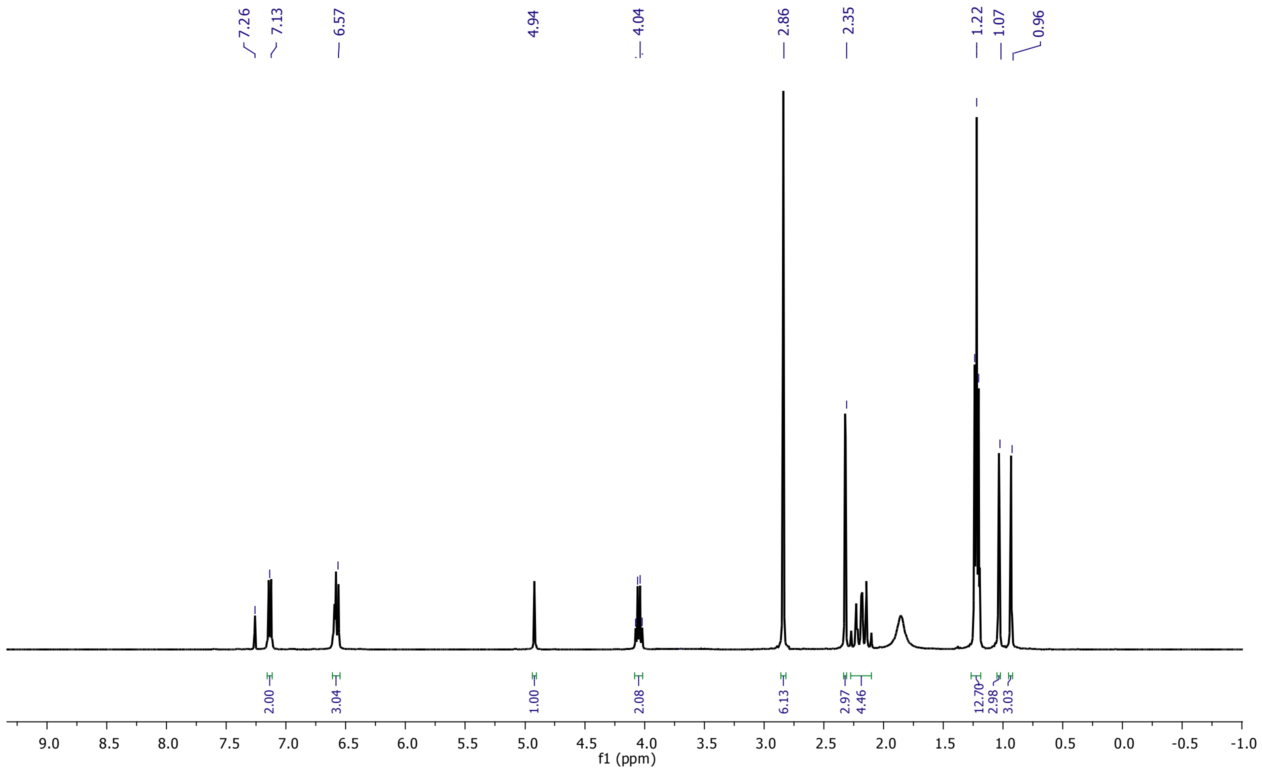

Figure S22. ^{1}H NMR spectrum of compound **5** ($CDCl_3$, 400 MHz, TMS).

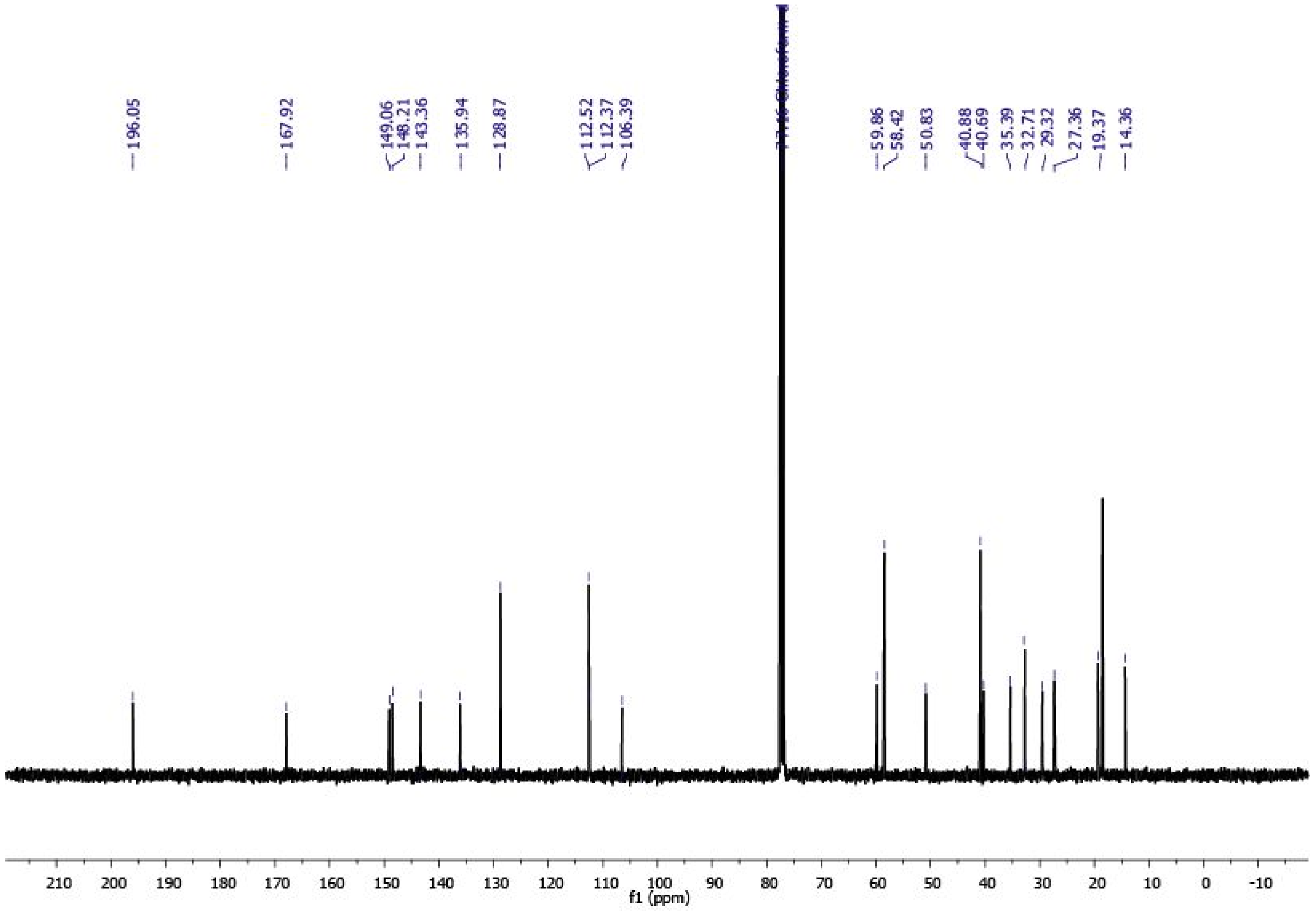

Figure S23.^{13}C NMR spectrum of compound **5** ($CDCl_3$, 100 MHz, TMS).

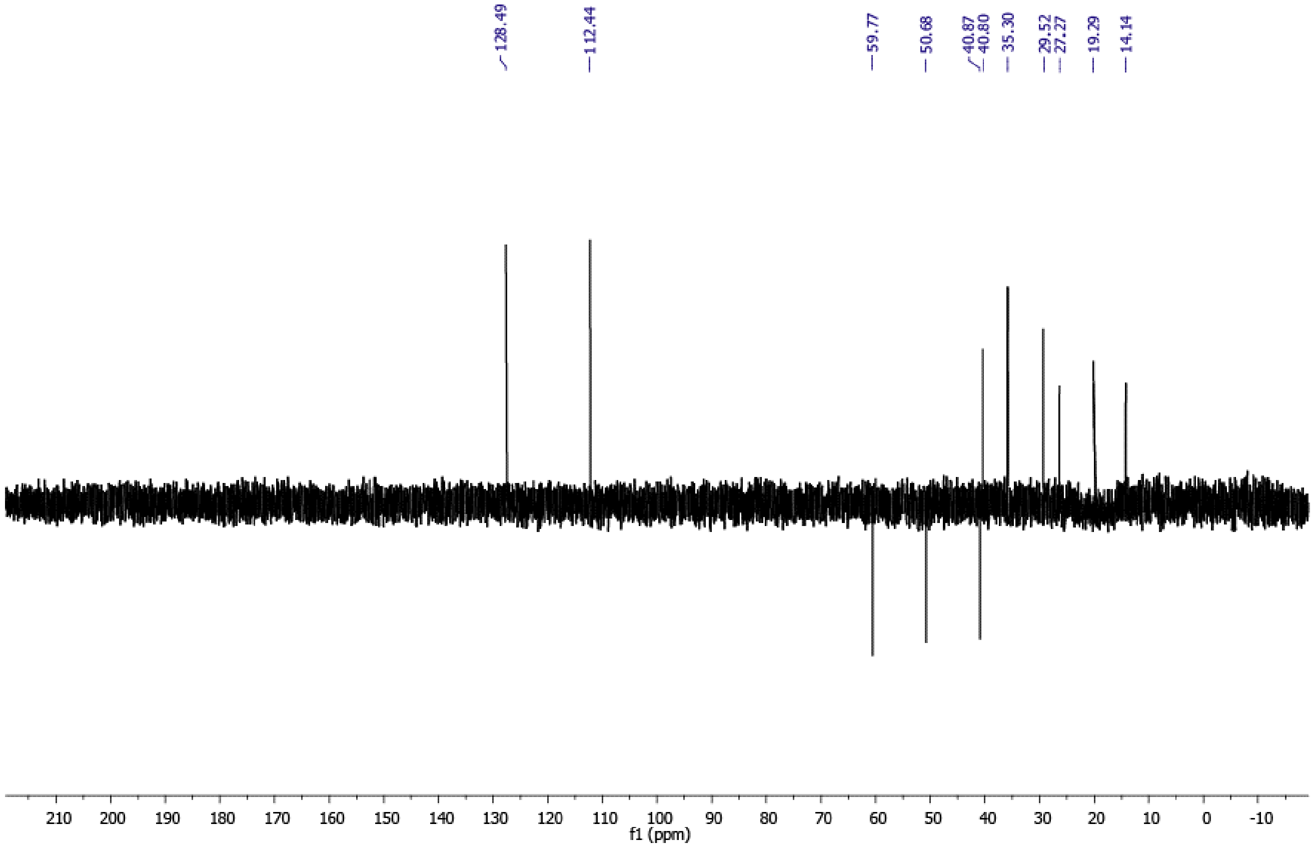

Figure S24. DEPT 135 spectrum of compound **5** ($CDCl_3$, 100 MHz, TMS).

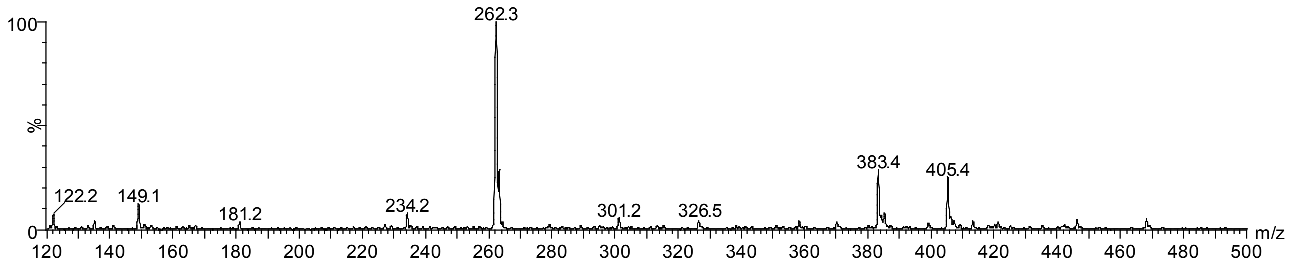

Figure S25. ESI (+) mass spectrum of compound **5**.

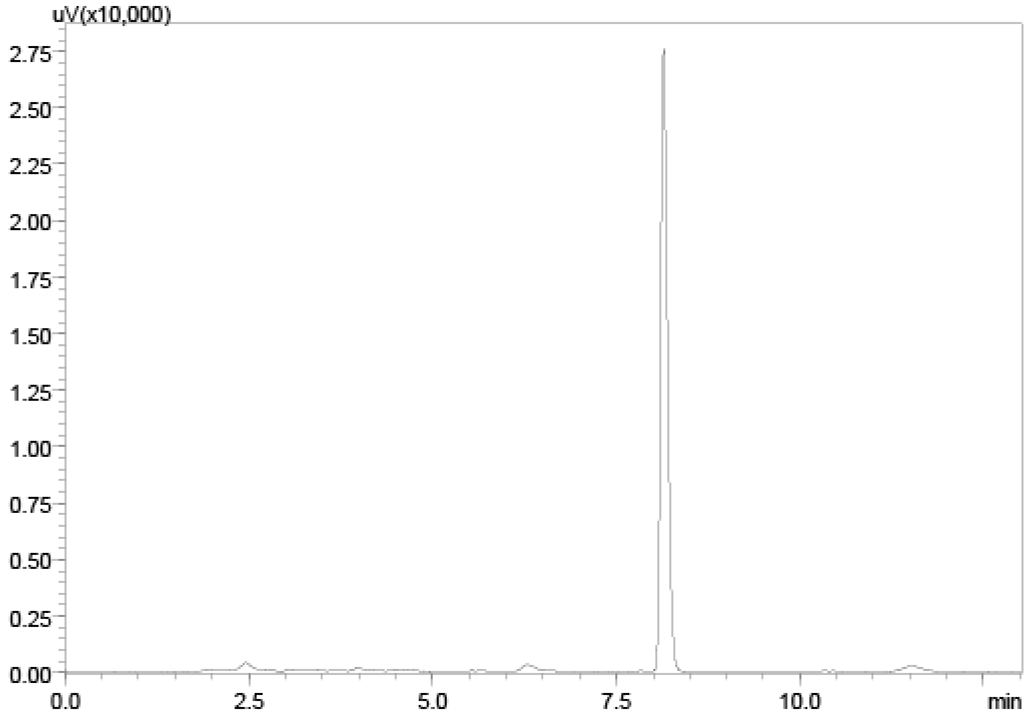

Figure S26: HPLC chromatogram of compound **6**.

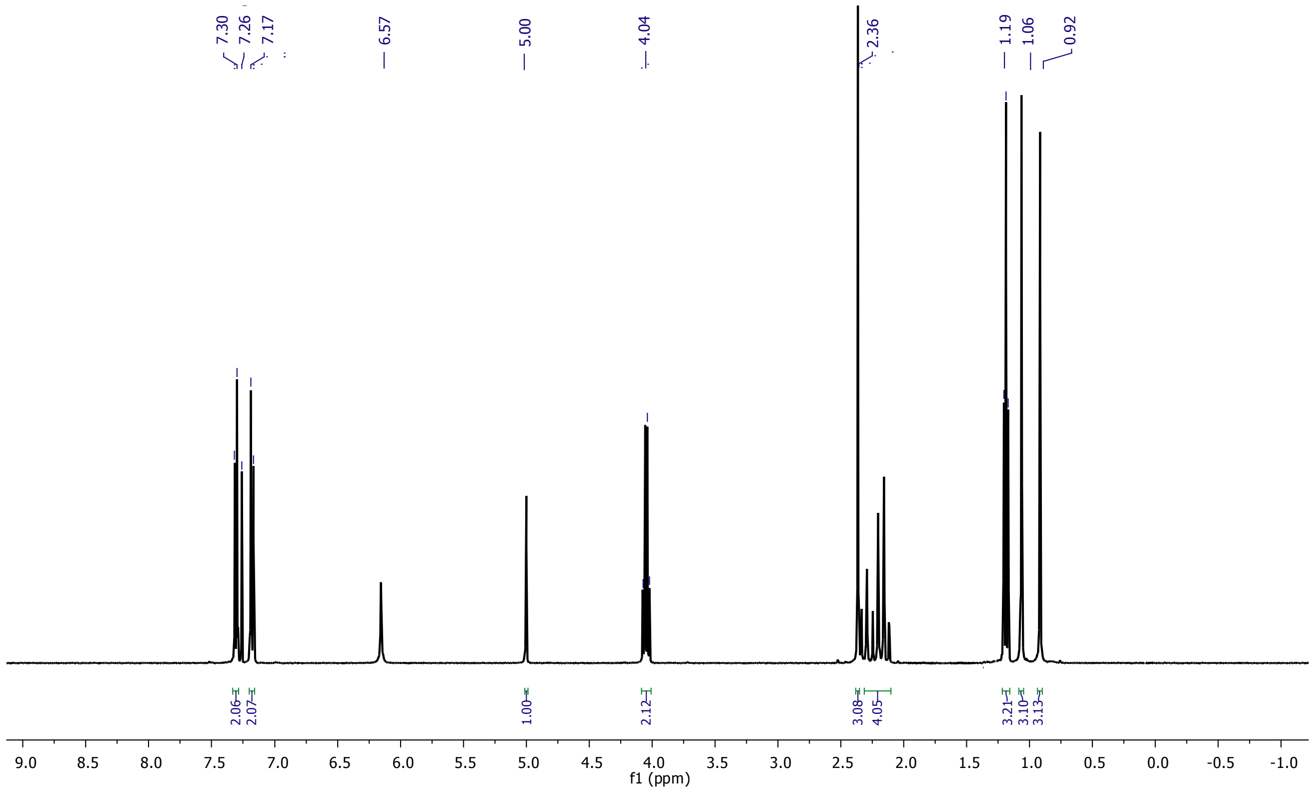

Figure S27.^{1}H NMR spectrum of compound **6** ($CDCl_3$, 400 MHz, TMS).

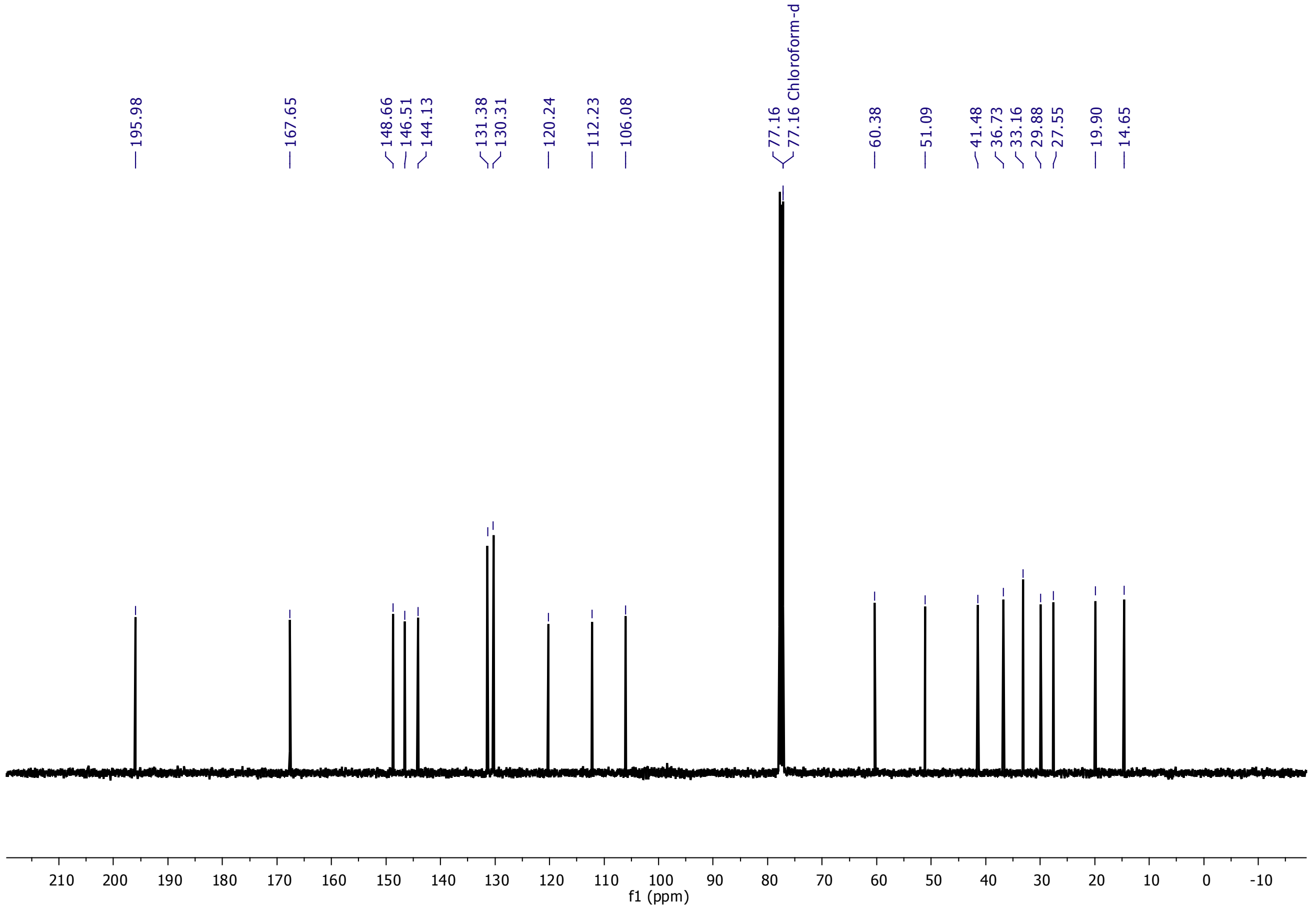

Figure S28.^{13}C NMR spectrum of compound **6** ($CDCl_3$, 100 MHz, TMS).

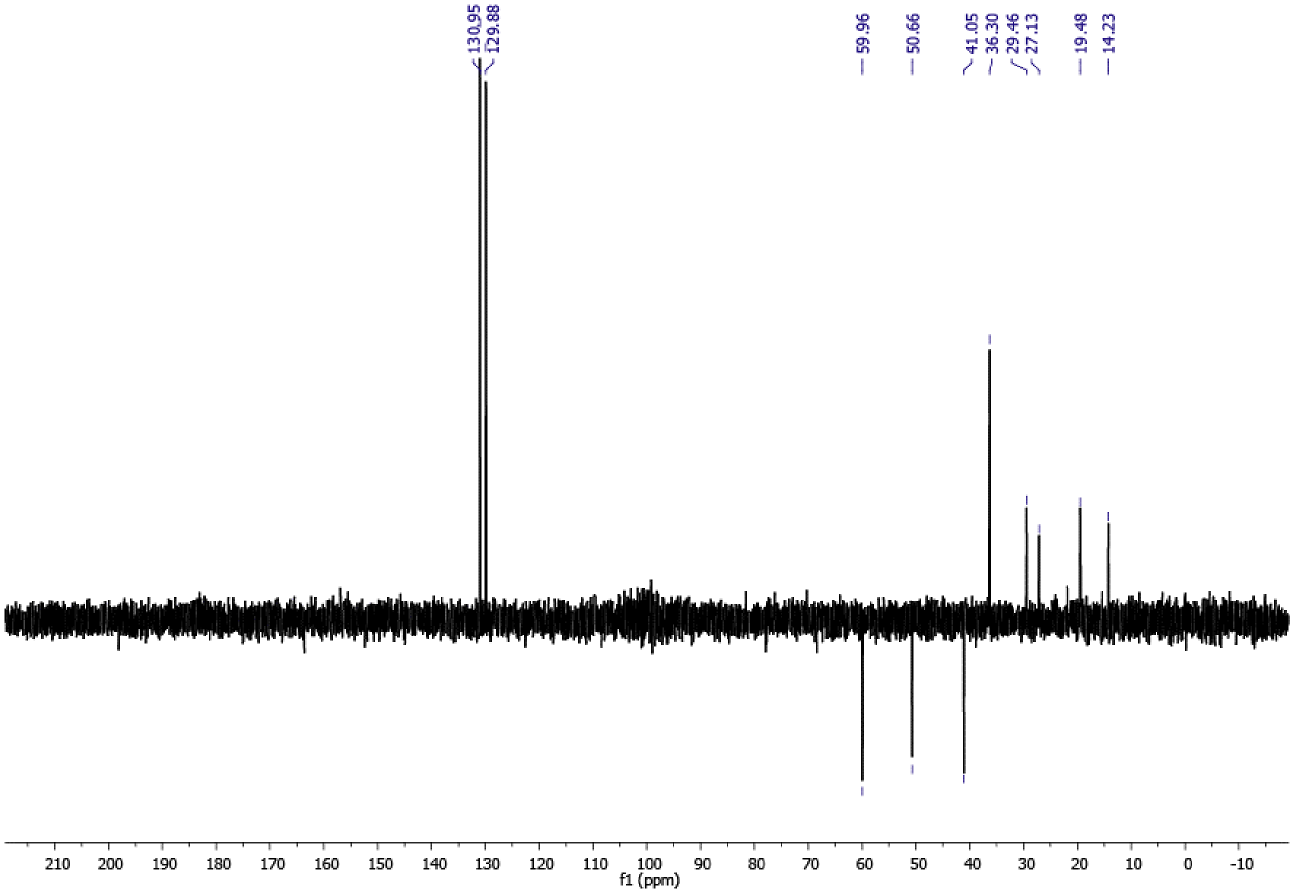

Figure S29. DEPT 135 spectrum of compound **6** ($CDCl_3$, 100 MHz, TMS).

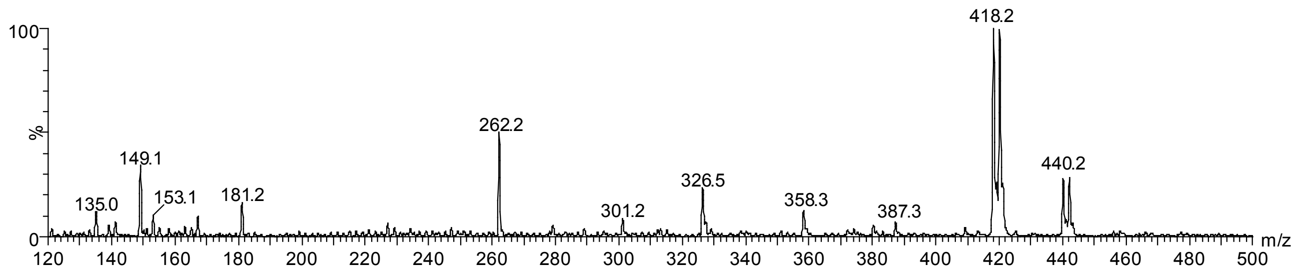

Figure S30. ESI (+) mass spectrum of compound **6**.

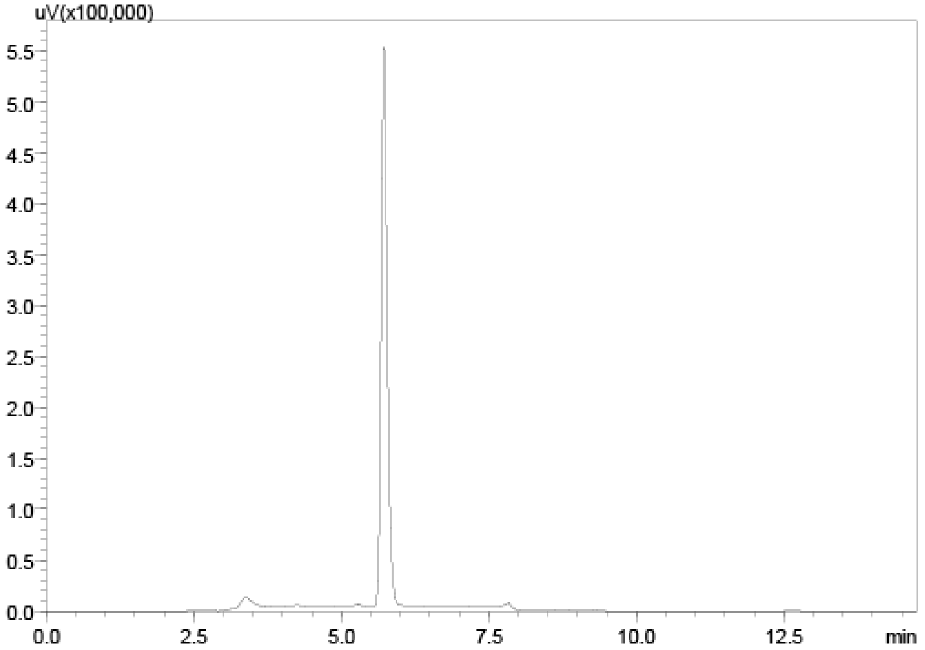

Figure S31: HPLC chromatogram of compound **7**.

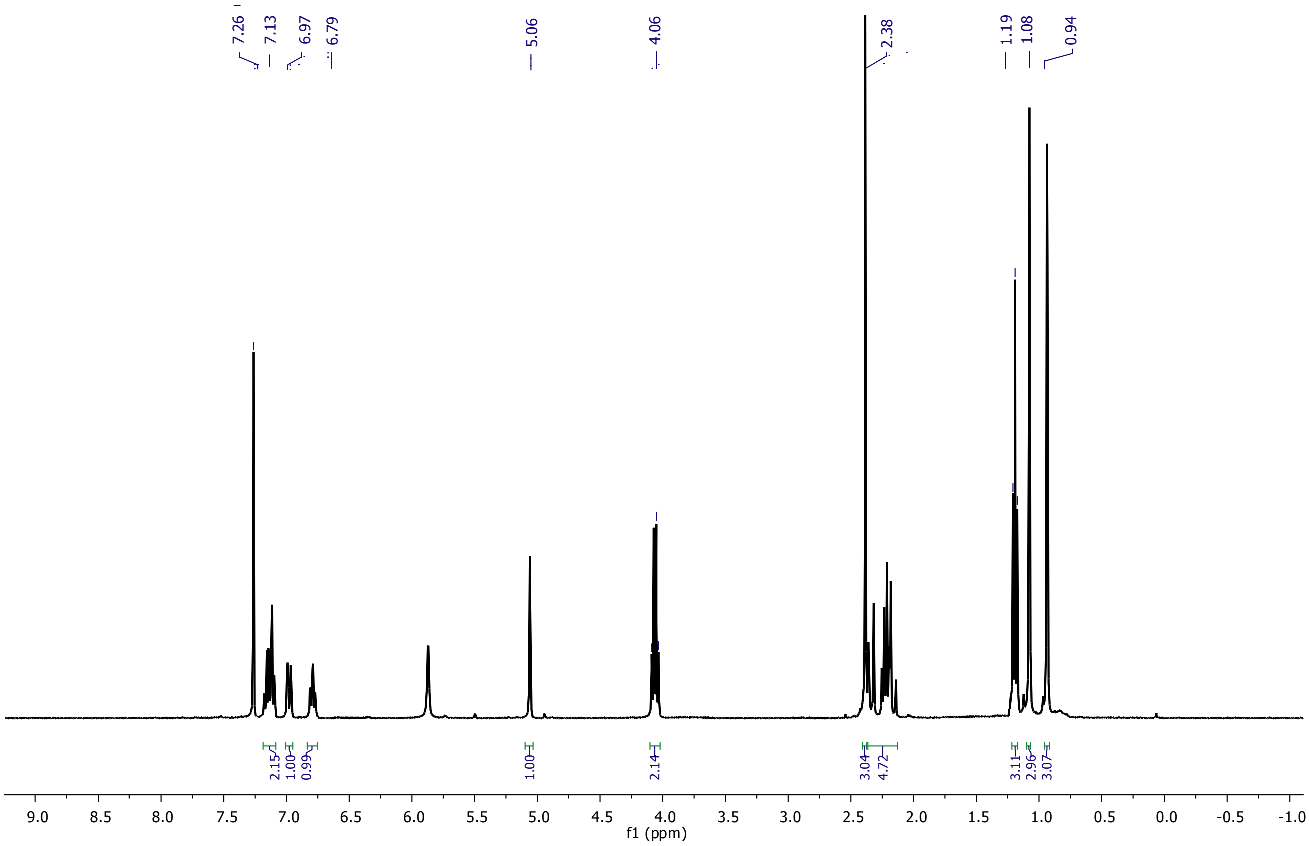

Figure S32.^{1}H NMR spectrum of compound **7** ($CDCl_3$, 400 MHz, TMS).

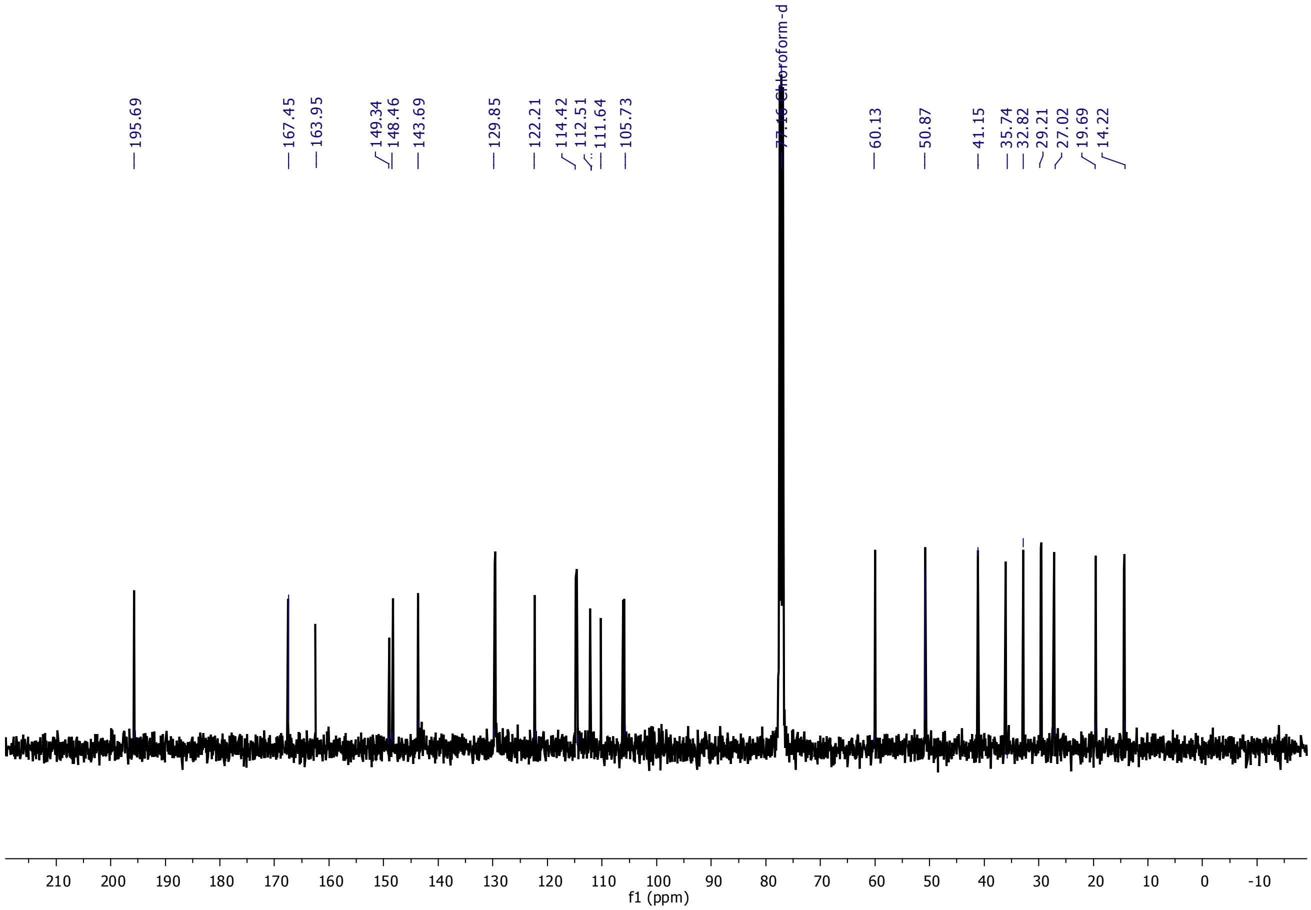

Figure S33.^{13}C NMR spectrum of compound **7** ($CDCl_3$, 100 MHz, TMS).

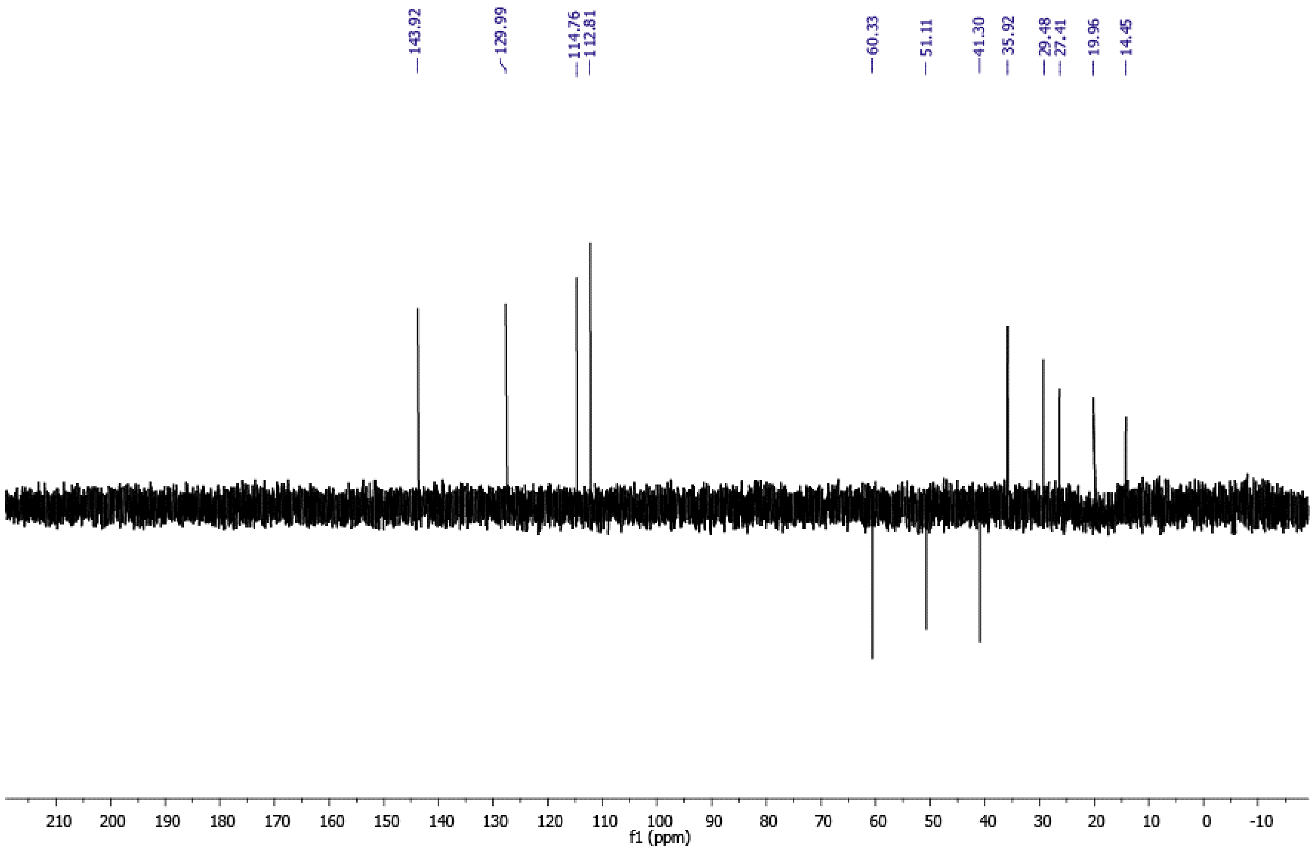

Figure S34. DEPT 135 spectrum of compound **7** ($CDCl_3$, 100 MHz, TMS).

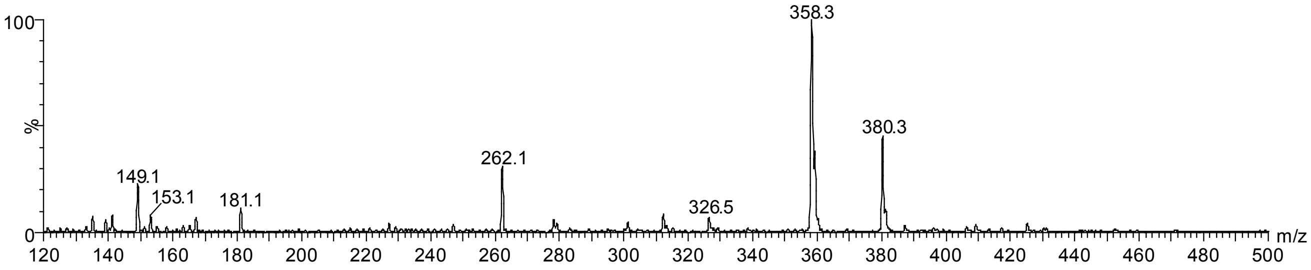

Figure S35. ESI (+) mass spectrum of compound **7**.

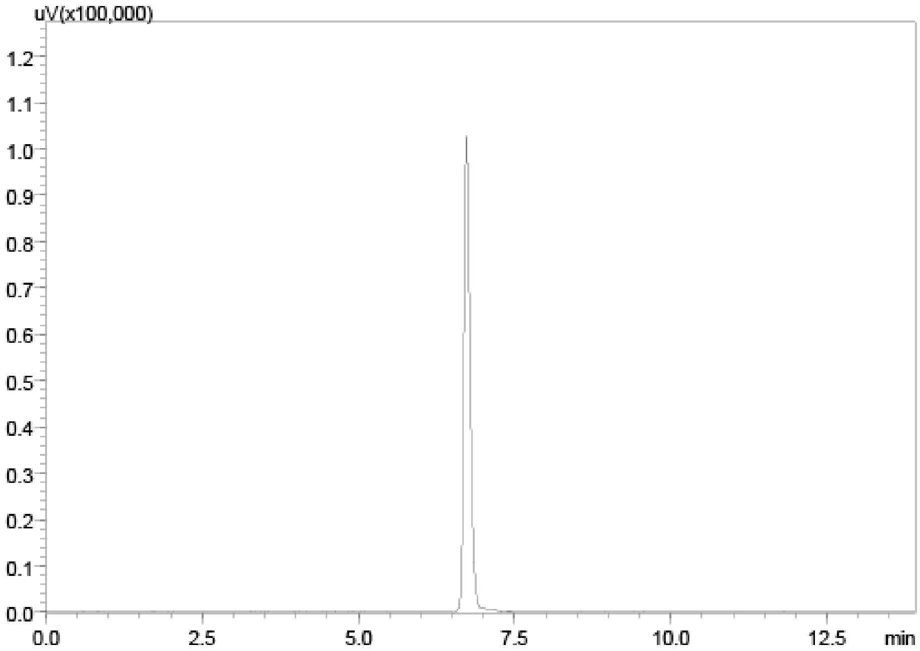

Figure S36: HPLC chromatogram of compound **8**.

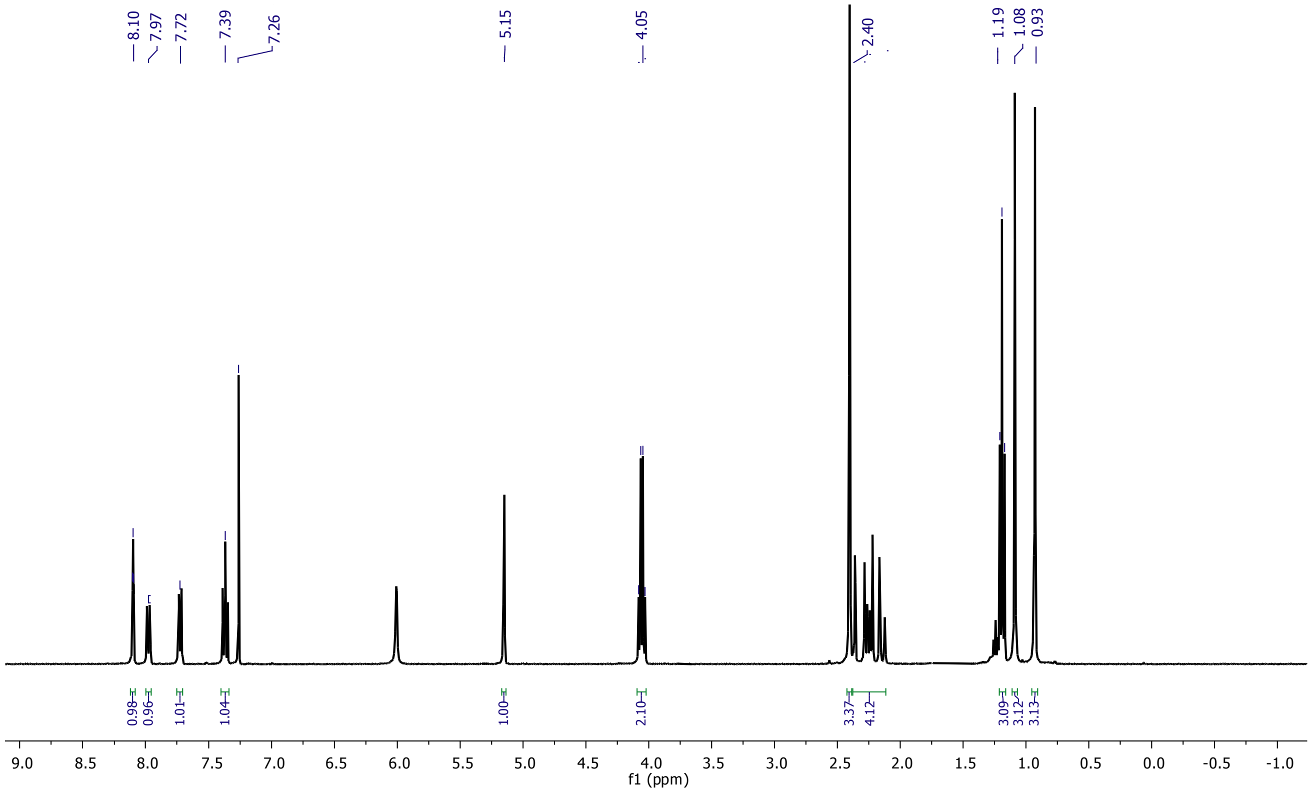

Figure S37.^{1}H NMR spectrum of compound **8** ($CDCl_3$, 400 MHz, TMS).

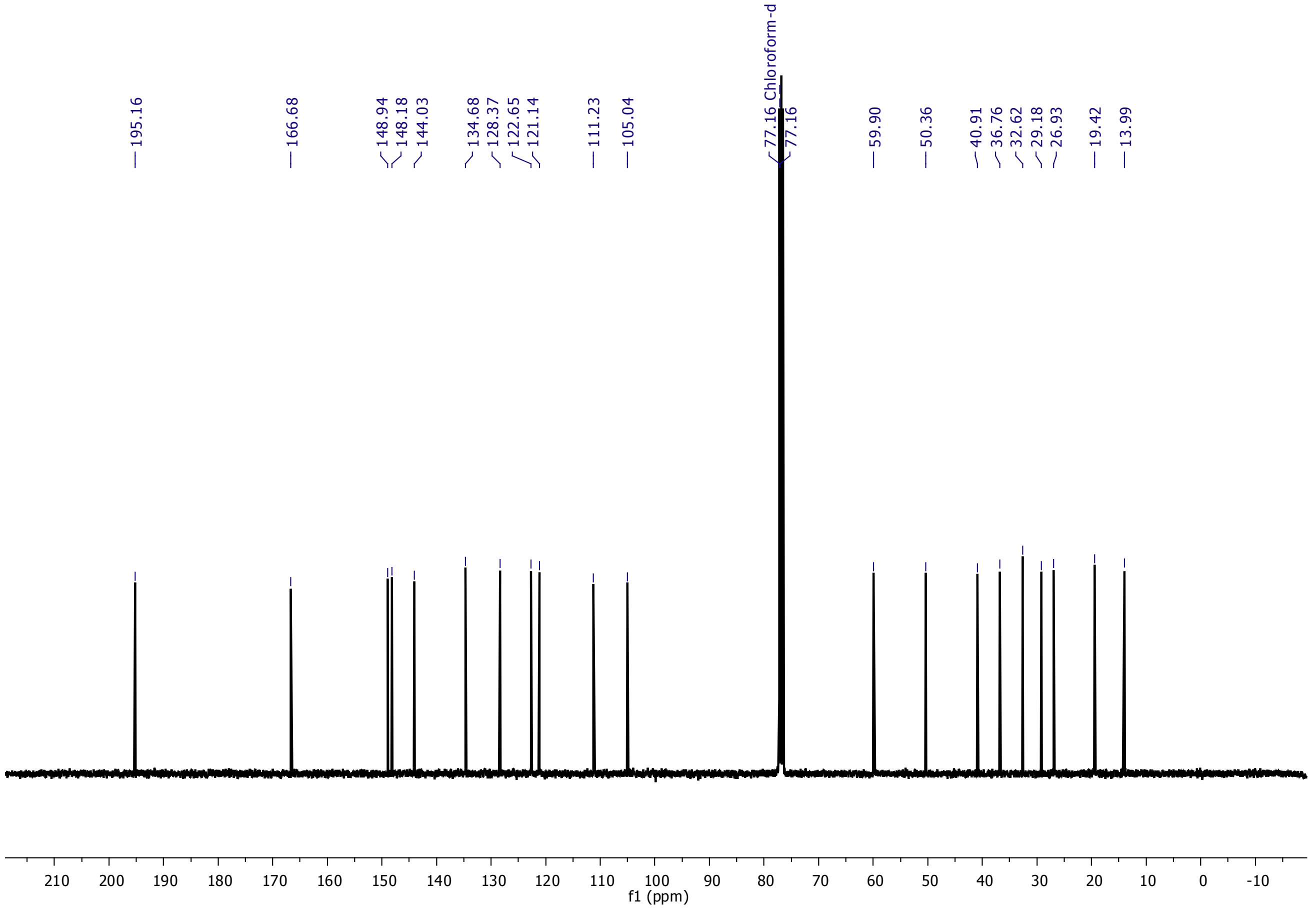

Figure S38.^{13}C NMR spectrum of compound **8** ($CDCl_3$, 100 MHz, TMS).

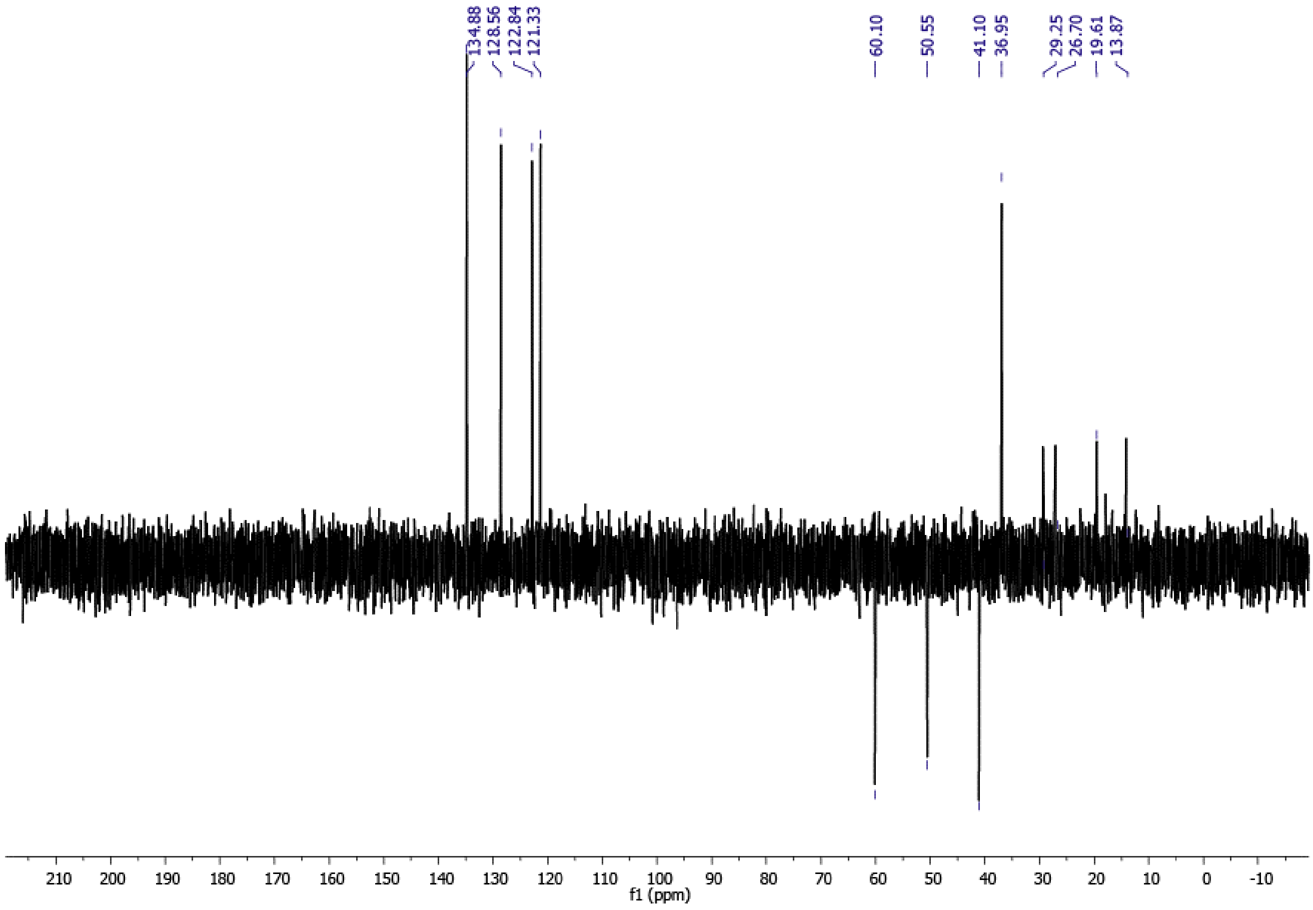

Figure S39. DEPT 135 spectrum of compound **8**($CDCl_3$, 100 MHz, TMS).

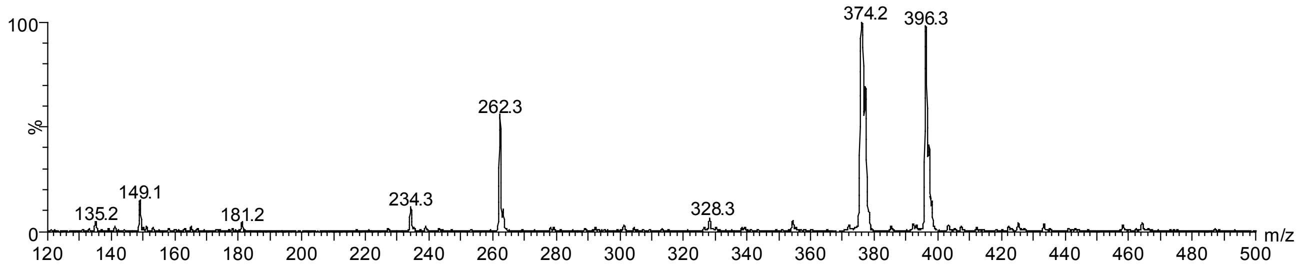

Figure S40. ESI (+) mass spectrum of compound **8**.

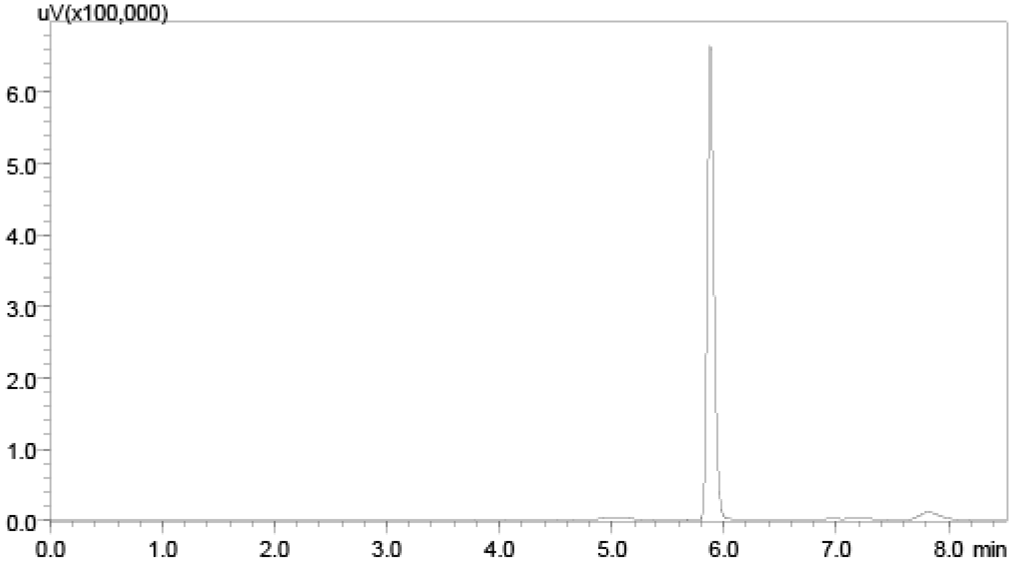

Figure S41: HPLC chromatogram of compound **9**.

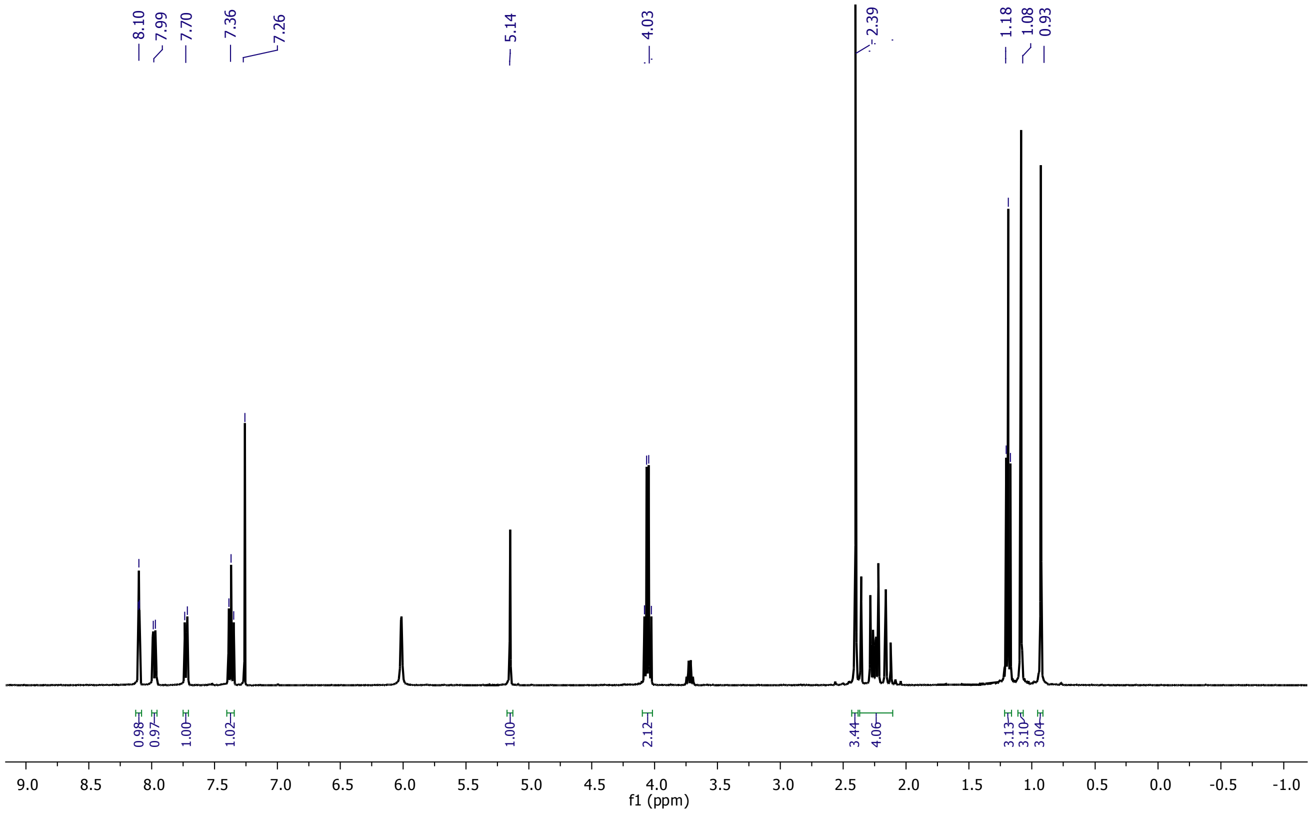

Figure S42.^{1}H NMR spectrum of compound **9** ($CDCl_3$, 400 MHz, TMS).

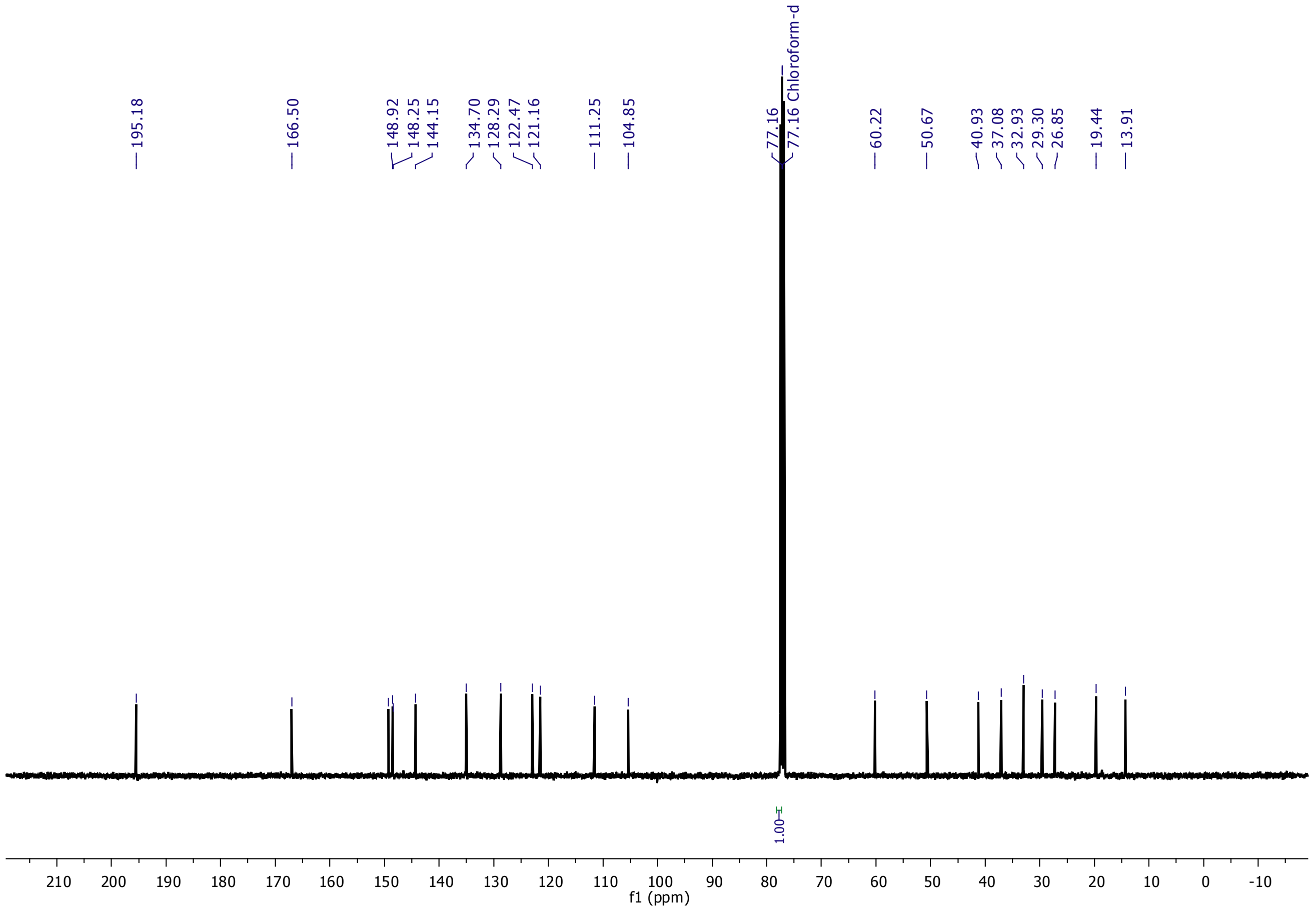

Figure S43.^{13}C NMR spectrum of compound **9** ($CDCl_3$, 100 MHz, TMS).

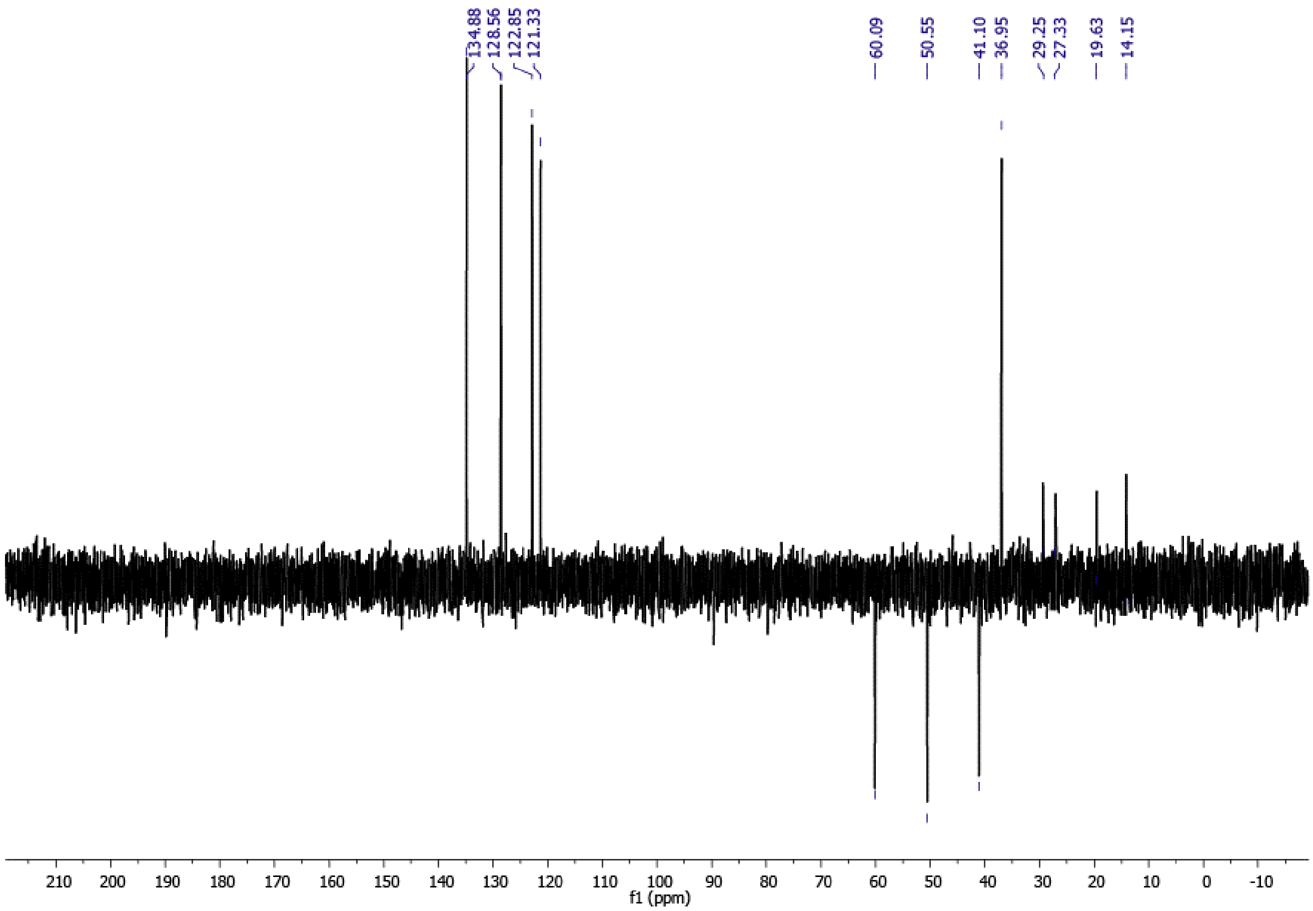

Figure S44. DEPT 135 spectrum of compound **9** ($CDCl_3$, 100 MHz, TMS).

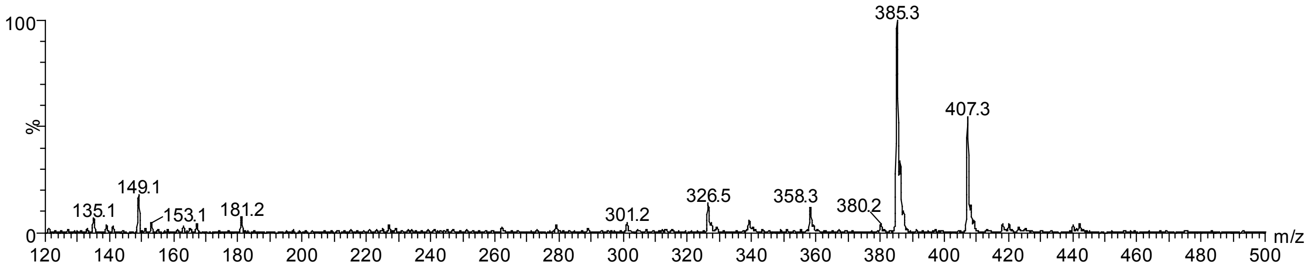

Figure S45. ESI (+) mass spectrum of compound **9**.

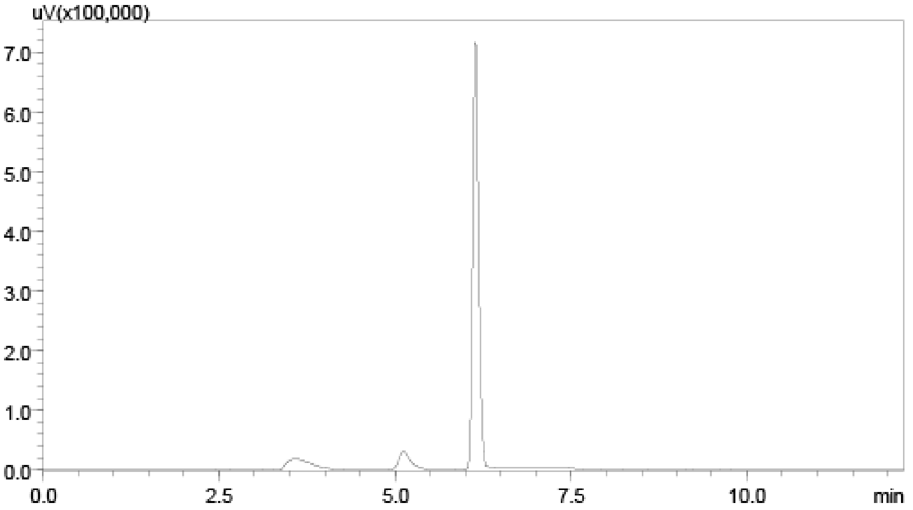

Figure S46: HPLC chromatogram of compound **10**.

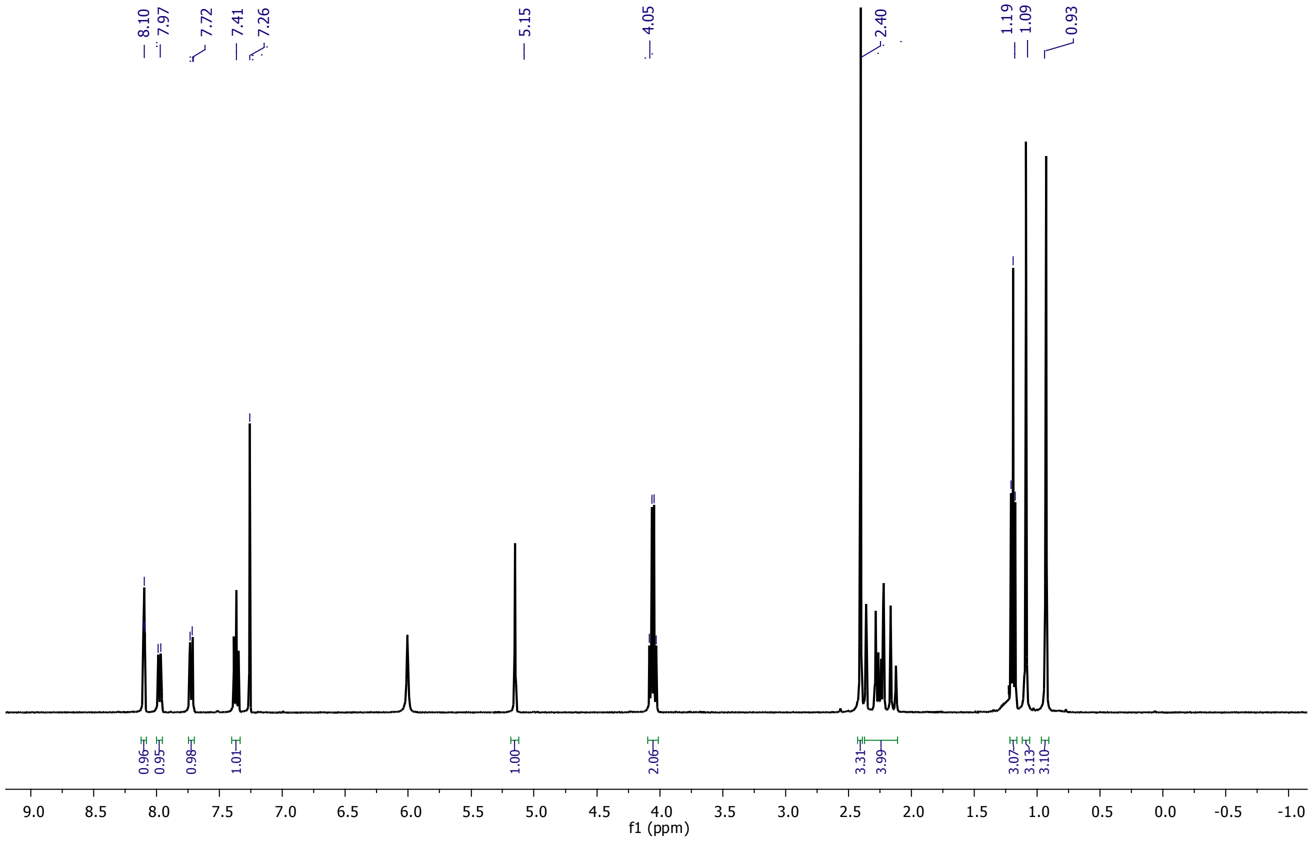

Figure S47.^{1}H NMR spectrum of compound **10** ($CDCl_3$, 400 MHz, TMS).

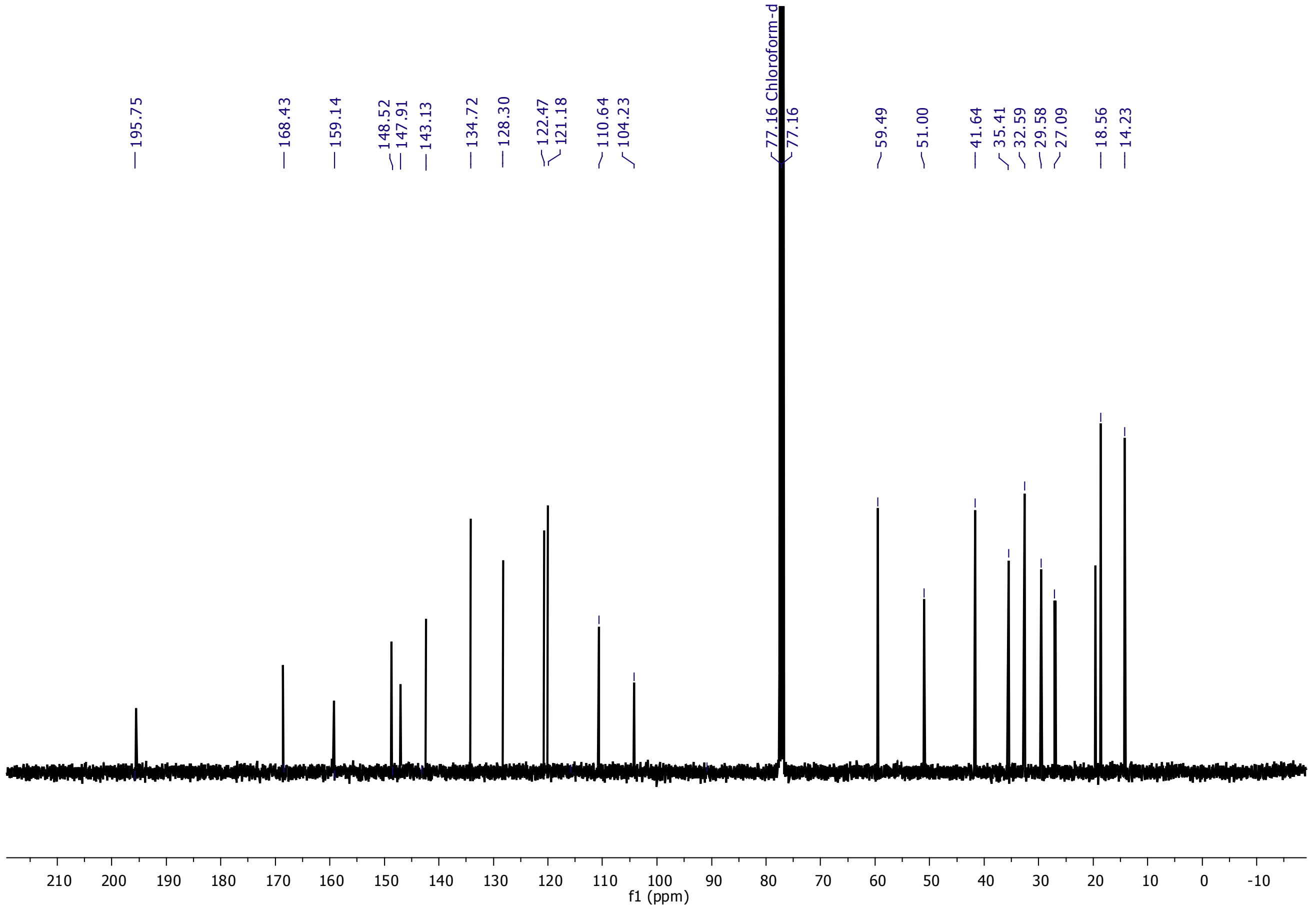

Figure S48.^{13}C NMR spectrum of compound **10** ($CDCl_3$, 100 MHz, TMS).

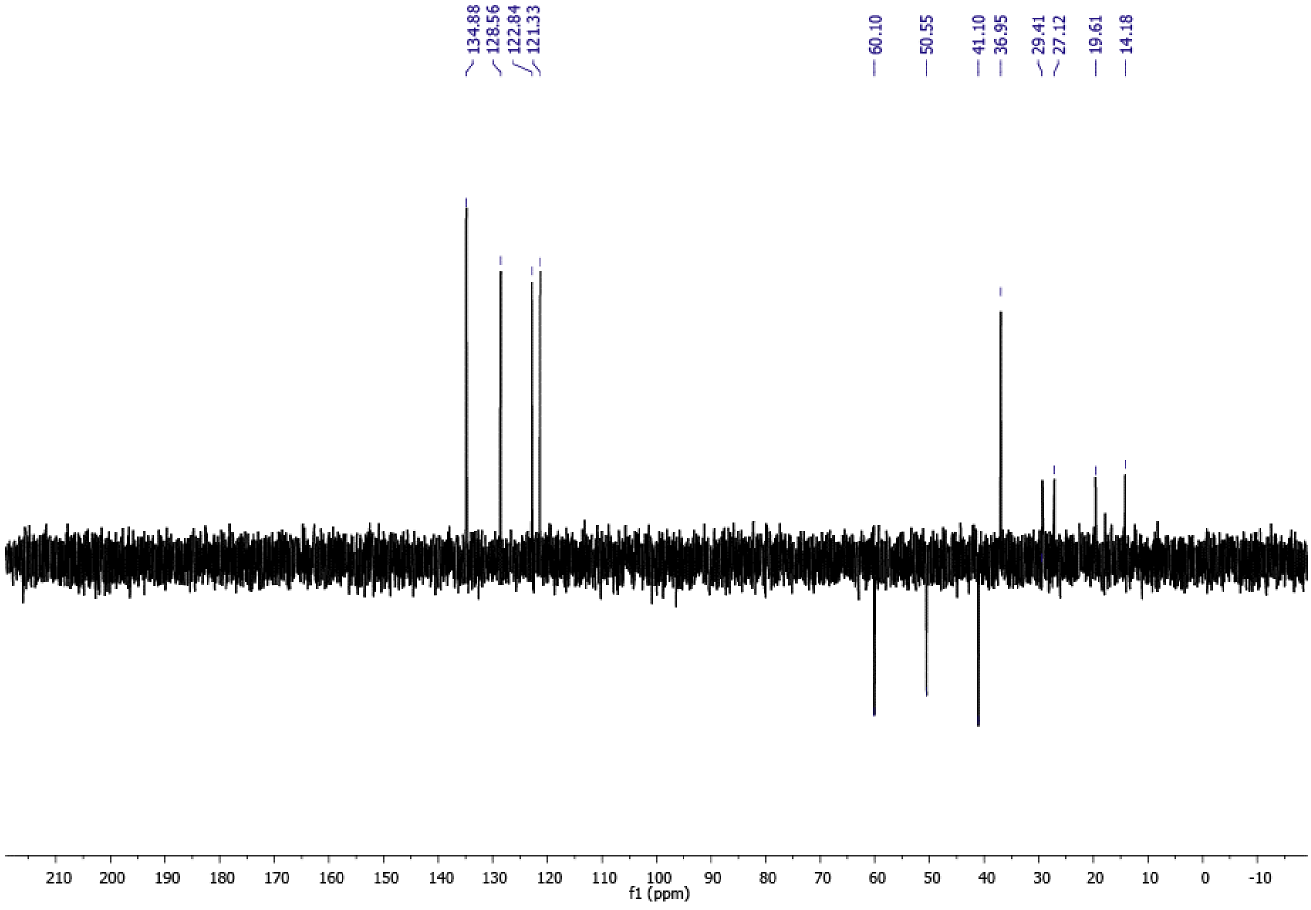

Figure S49. DEPT 135 spectrum of compound **10** ($CDCl_3$, 100 MHz, TMS).

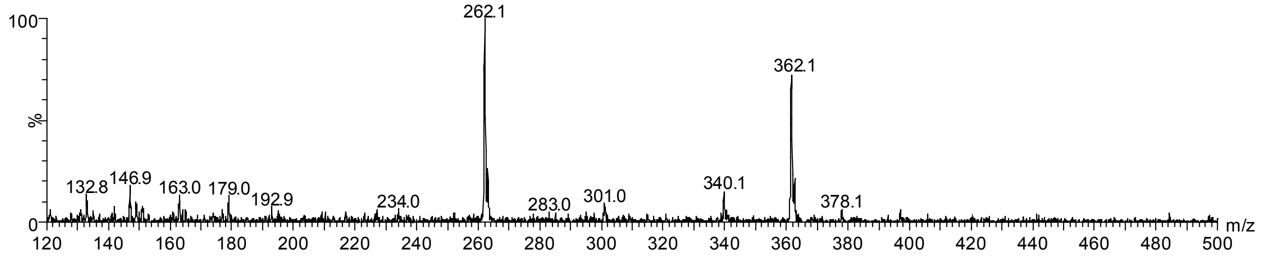

Figure S50. ESI (+) mass spectrum of compound **10.**

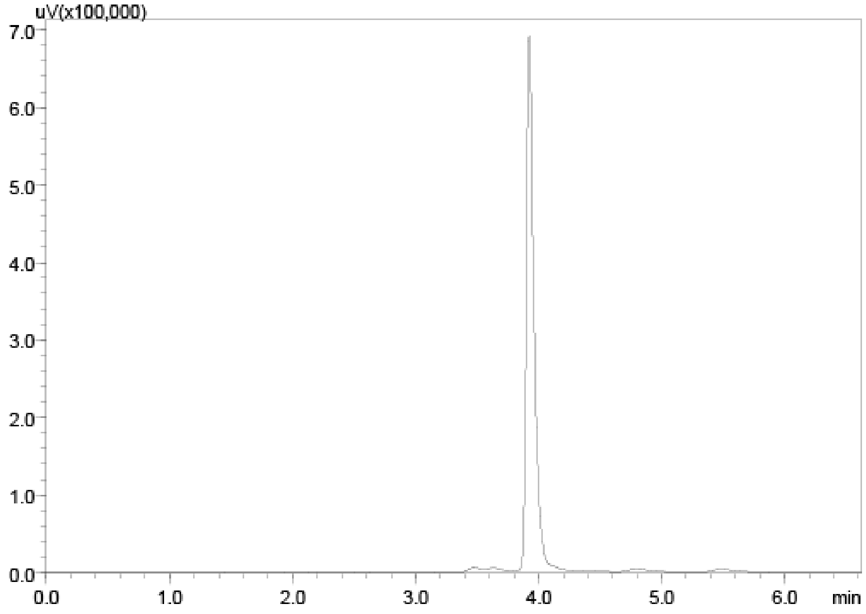

Figure S51: HPLC chromatogram of compound **11**.

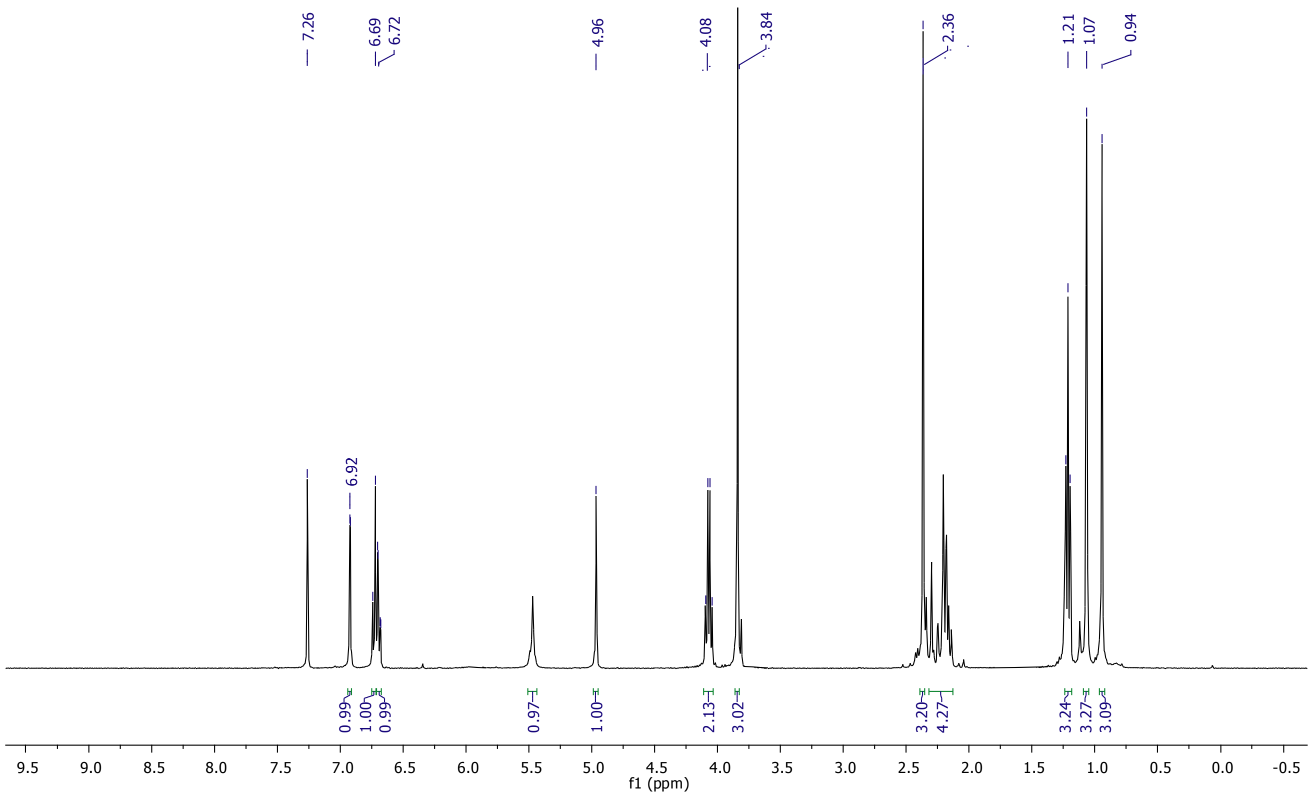

Figure S52.^{1}H NMR spectrum of compound **11** ($CDCl_3$, 400 MHz, TMS).

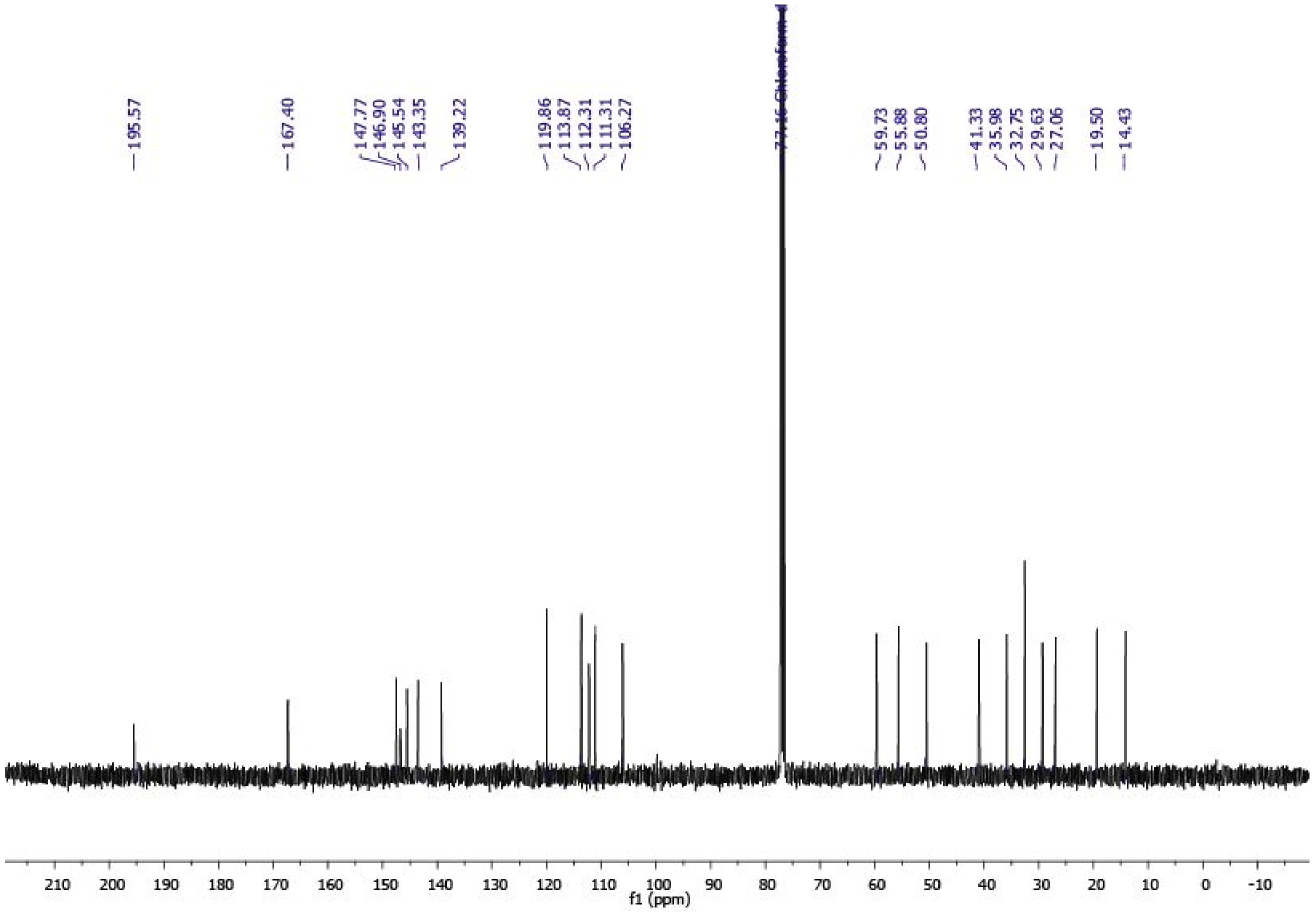

Figure S53.^{13}C NMR spectrum of compound **11** ($CDCl_3$, 100 MHz, TMS).

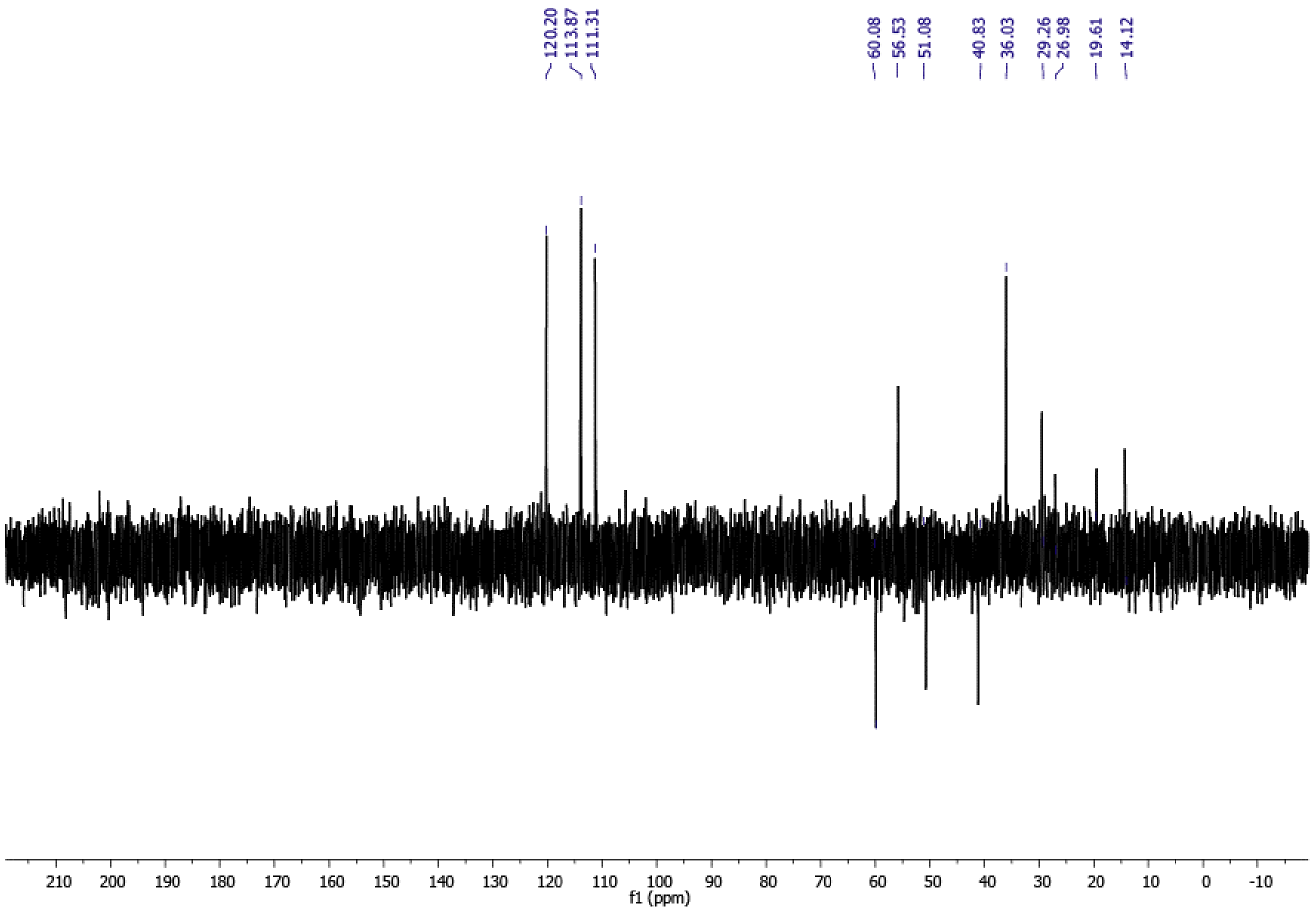

Figure S54. DEPT 135 spectrum of compound **11** ($CDCl_3$, 100 MHz, TMS).

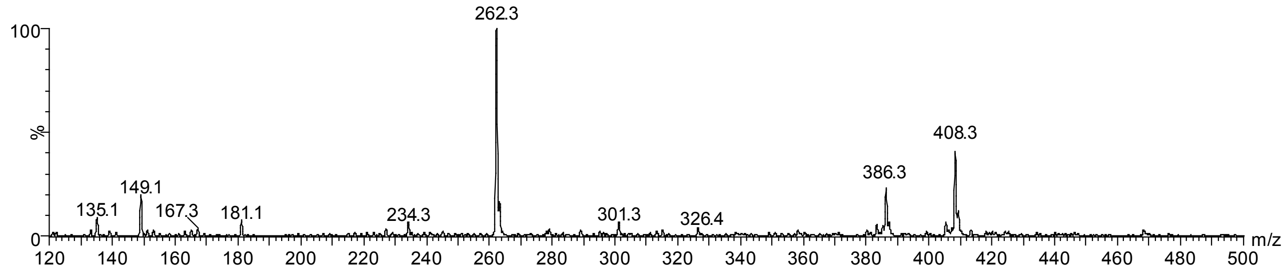

Figure S55. ESI (+) mass spectrum of compound **11.**

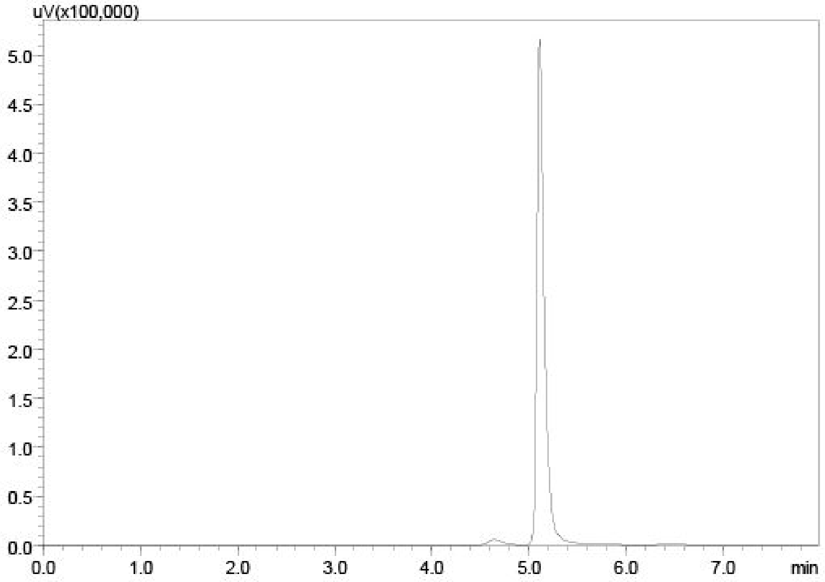

Figure S56: HPLC chromatogram of compound **12**.

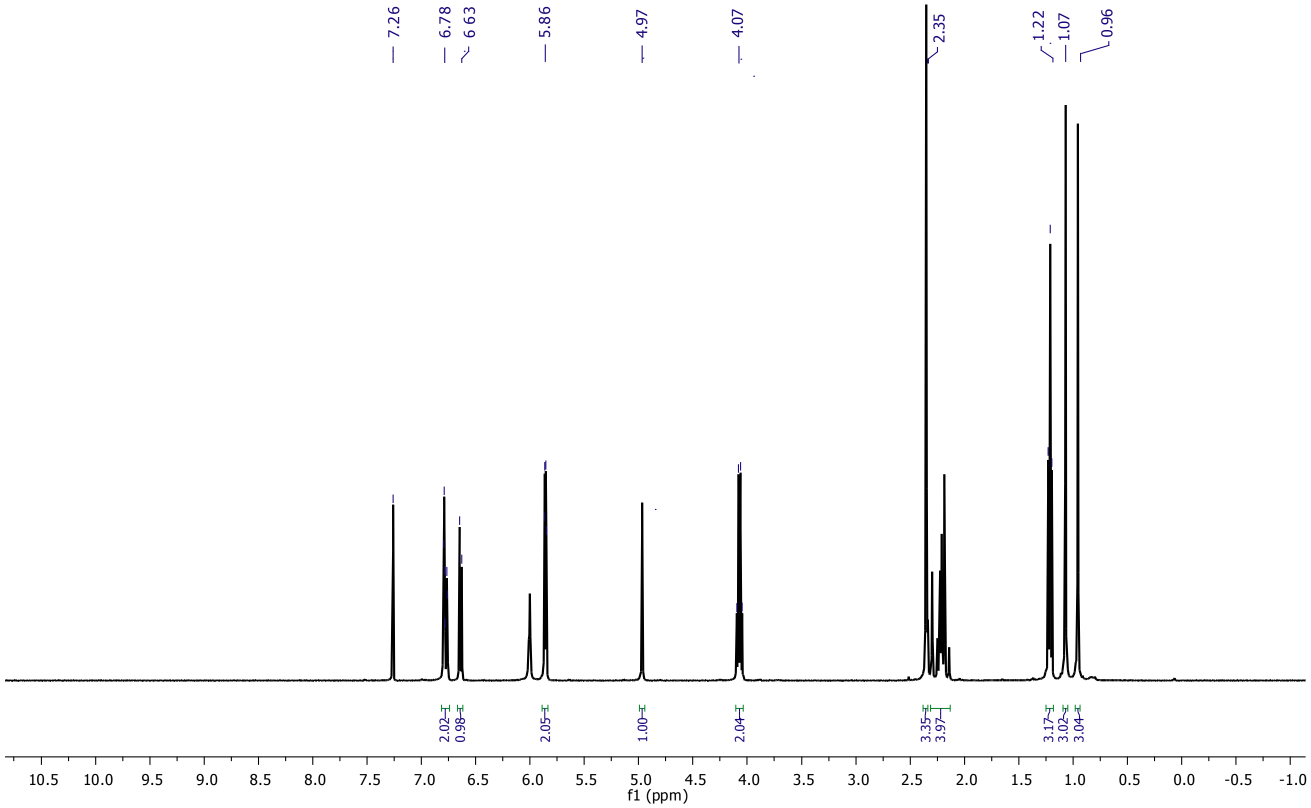

Figure S57.^{1}H NMR spectrum of compound **12** ($CDCl_3$, 400 MHz, TMS).

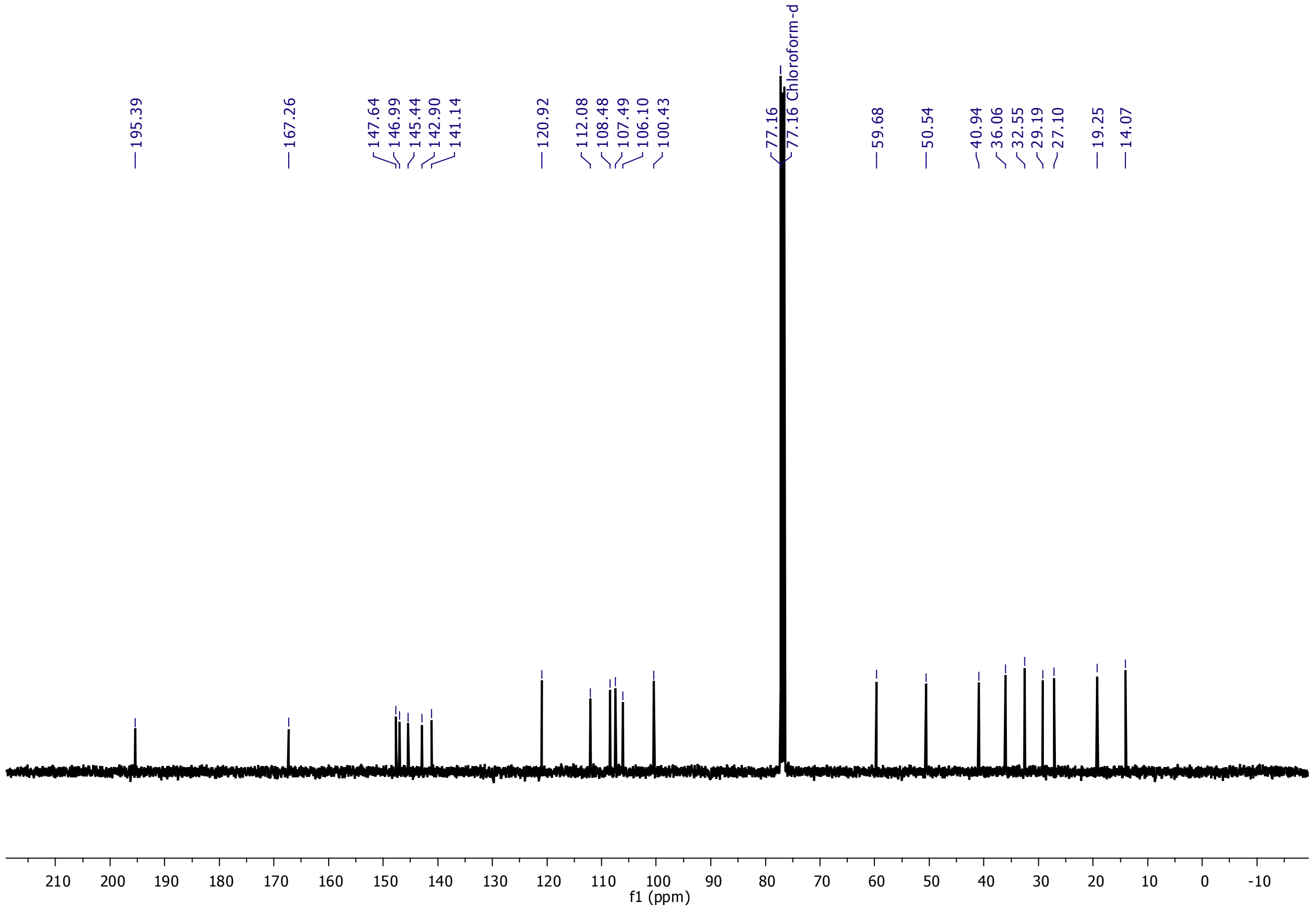

Figure S58.^{13}C NMR spectrum of compound **12** ($CDCl_3$, 100 MHz, TMS).

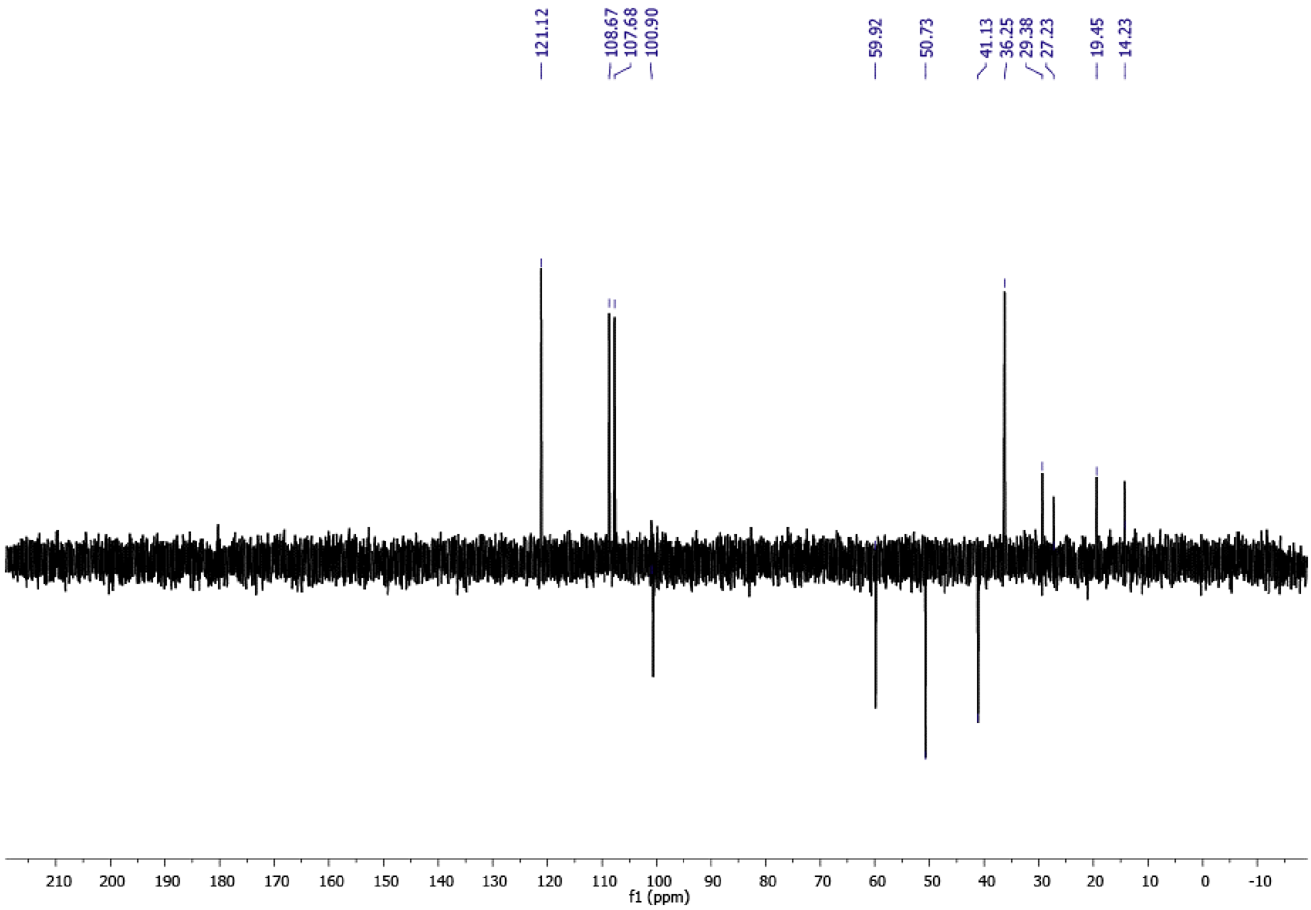

Figure S59. DEPT 135 spectrum of compound **12** ($CDCl_3$, 100 MHz, TMS).

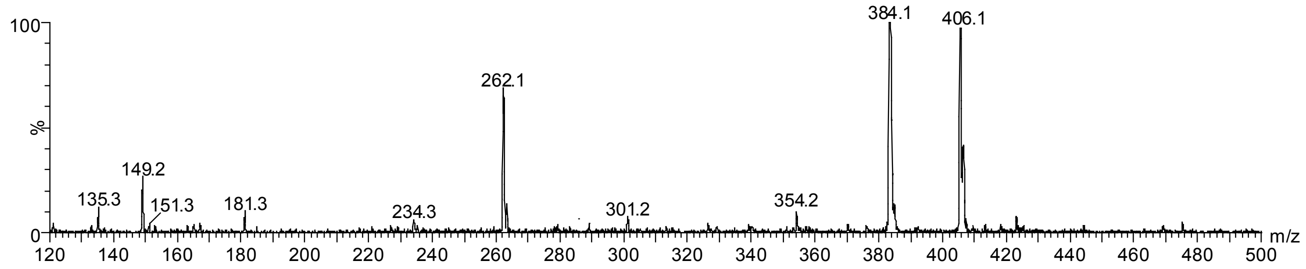

Figure S60. ESI (+) mass spectrum of compound **12.**

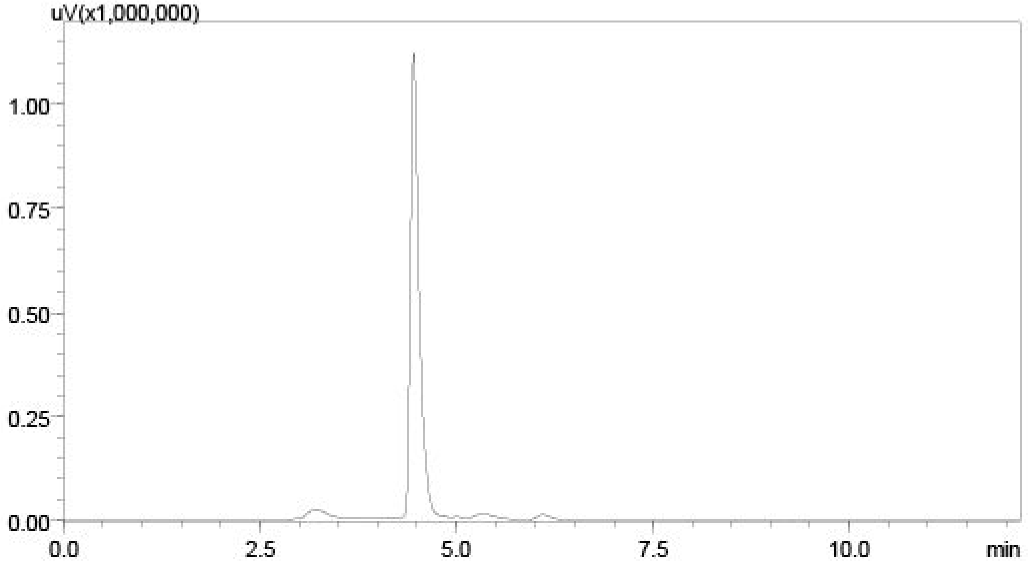

Figure S61: HPLC chromatogram of compound **13**.

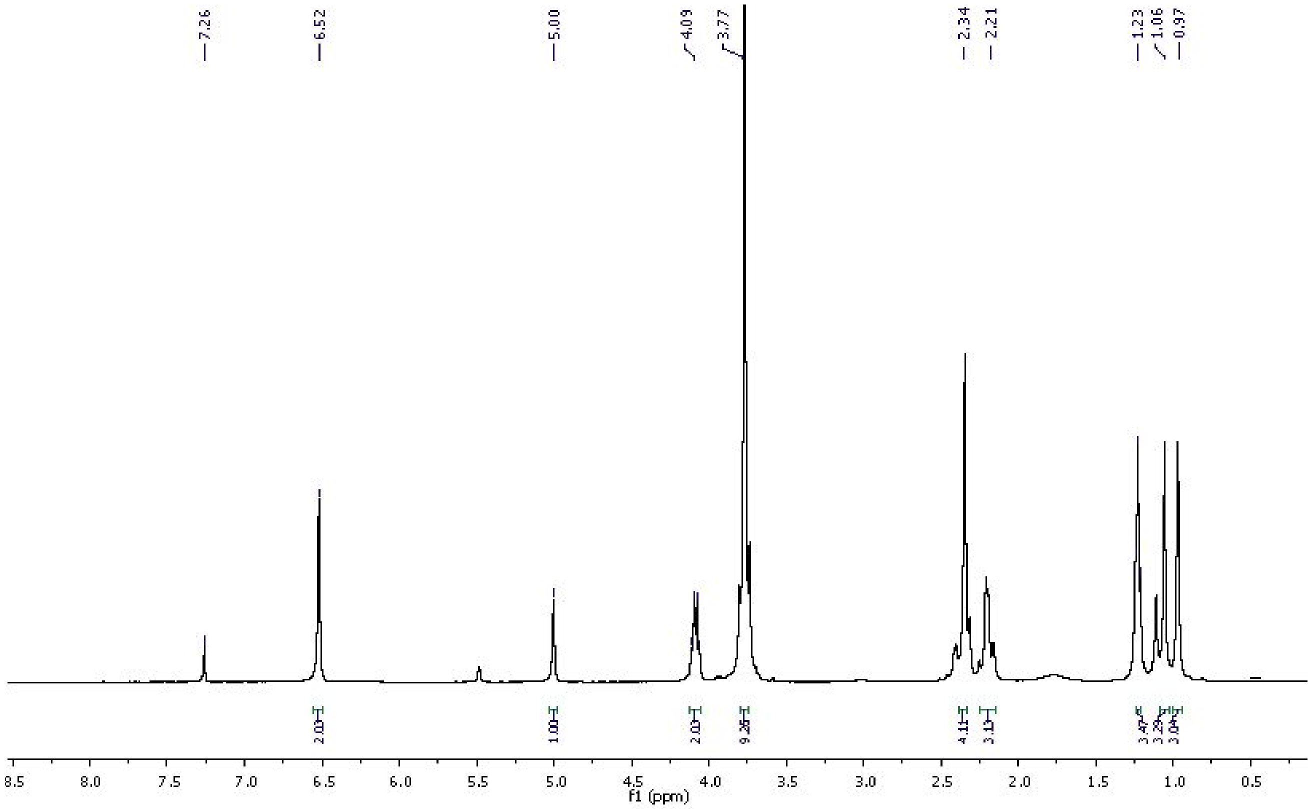

Figure S62.^{1}H NMR spectrum of compound **13** ($CDCl_3$, 400 MHz, TMS).

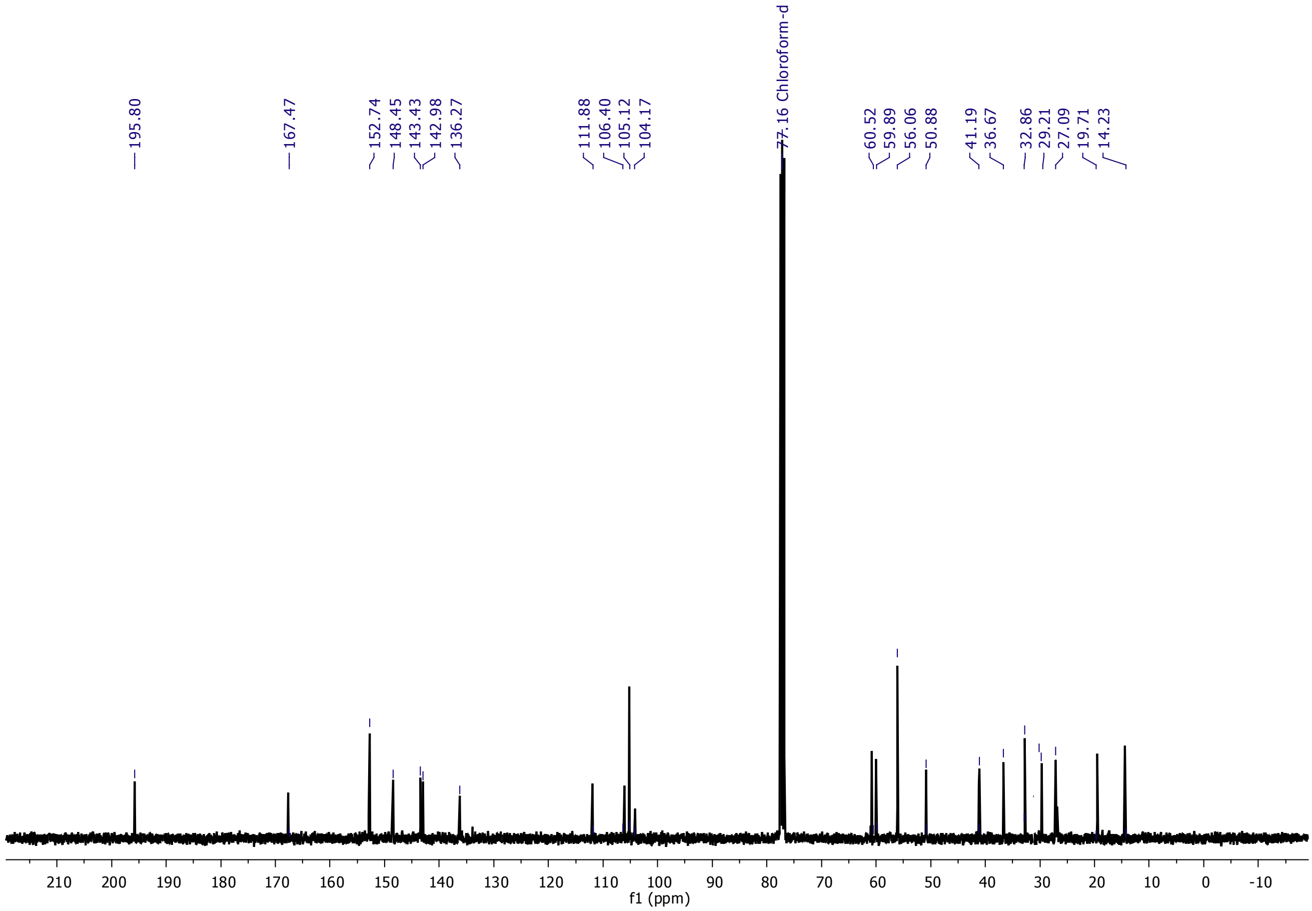

Figure S63.^{13}C NMR spectrum of compound **13** ($CDCl_3$, 100 MHz, TMS).

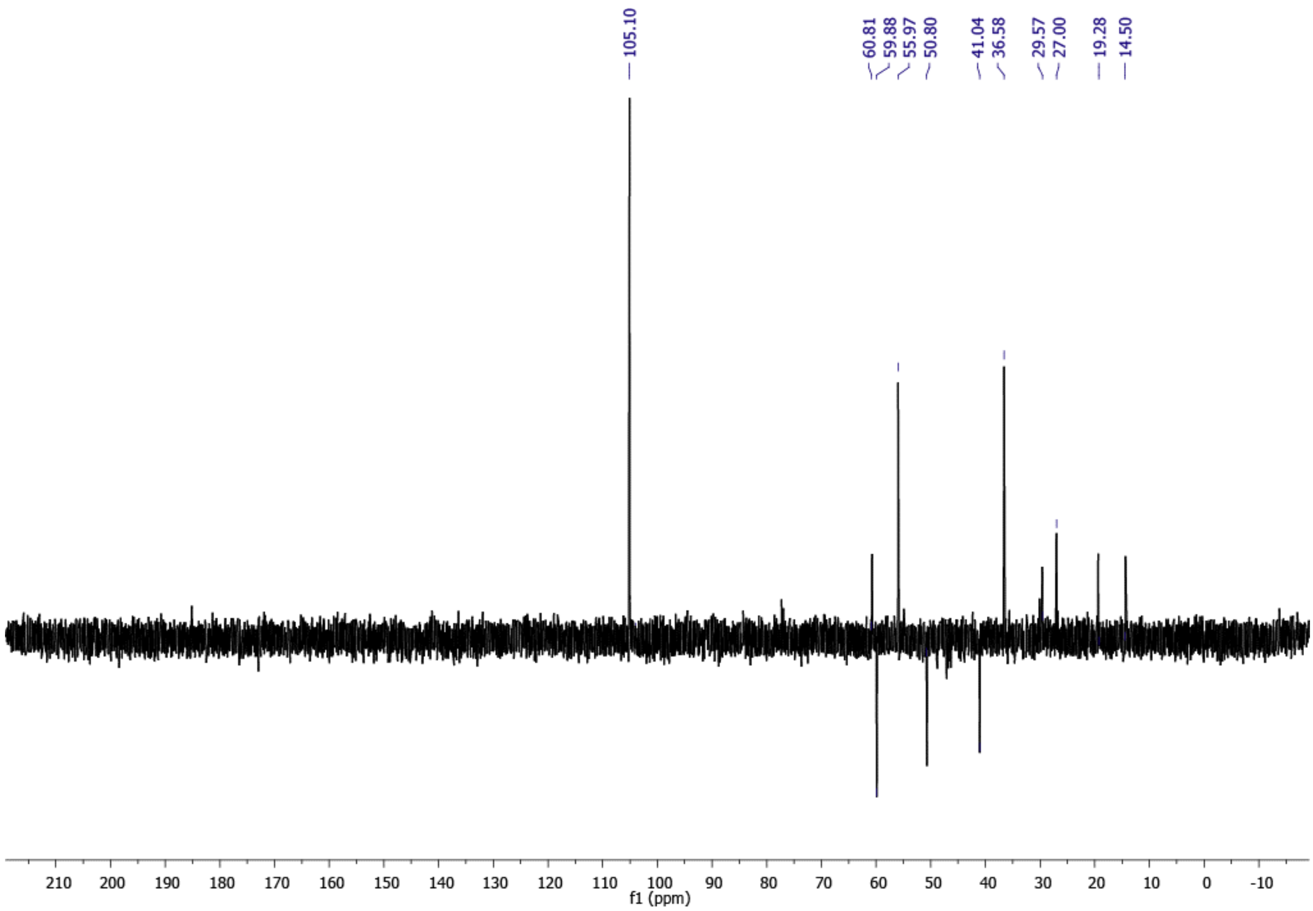

Figure S64. DEPT 135 spectrum of compound **13** ($CDCl_3$, 100 MHz, TMS).

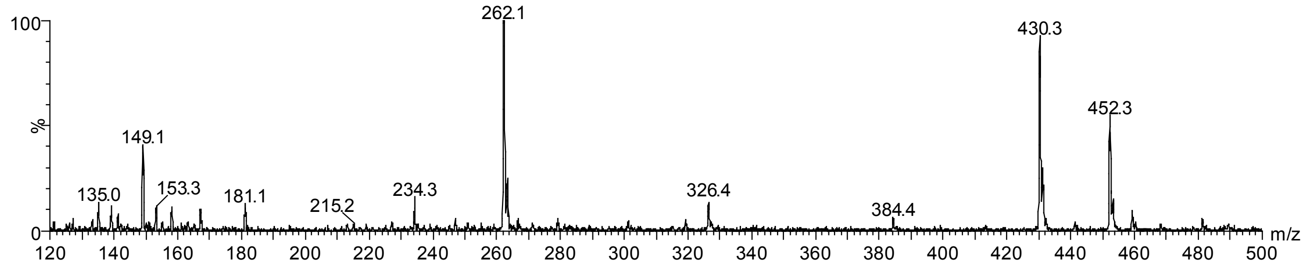

Figure S65. ESI (+) mass spectrum of compound **13.**

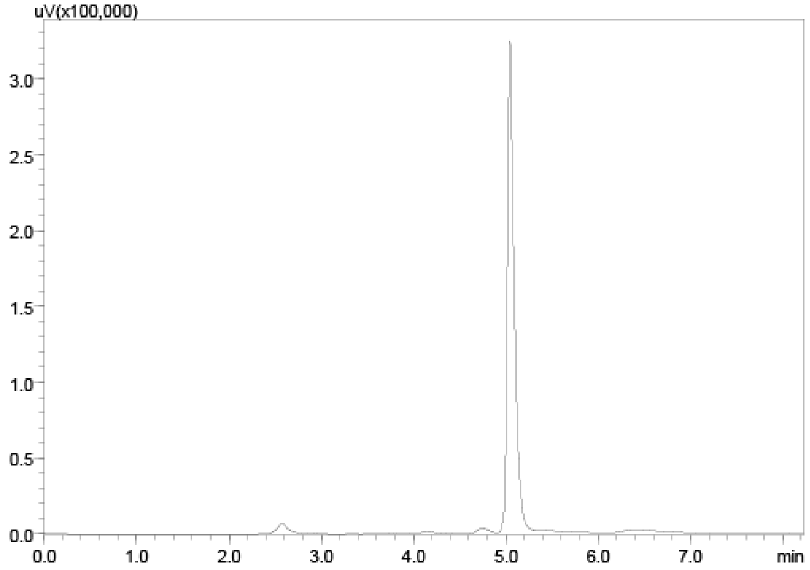

Figure S66: HPLC chromatogram of compound **14**.

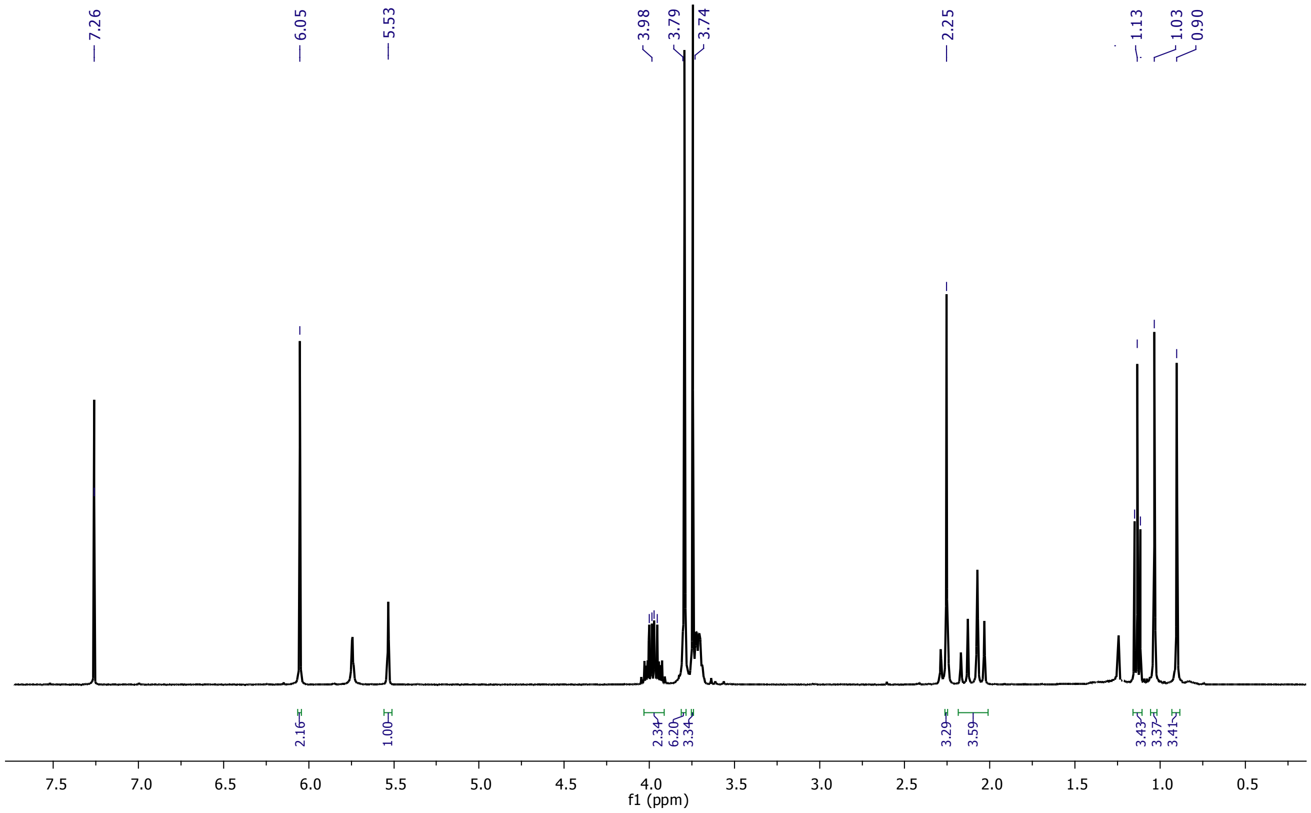

Figure S67.^{1}H NMR spectrum of compound **14** ($CDCl_3$, 400 MHz, TMS).

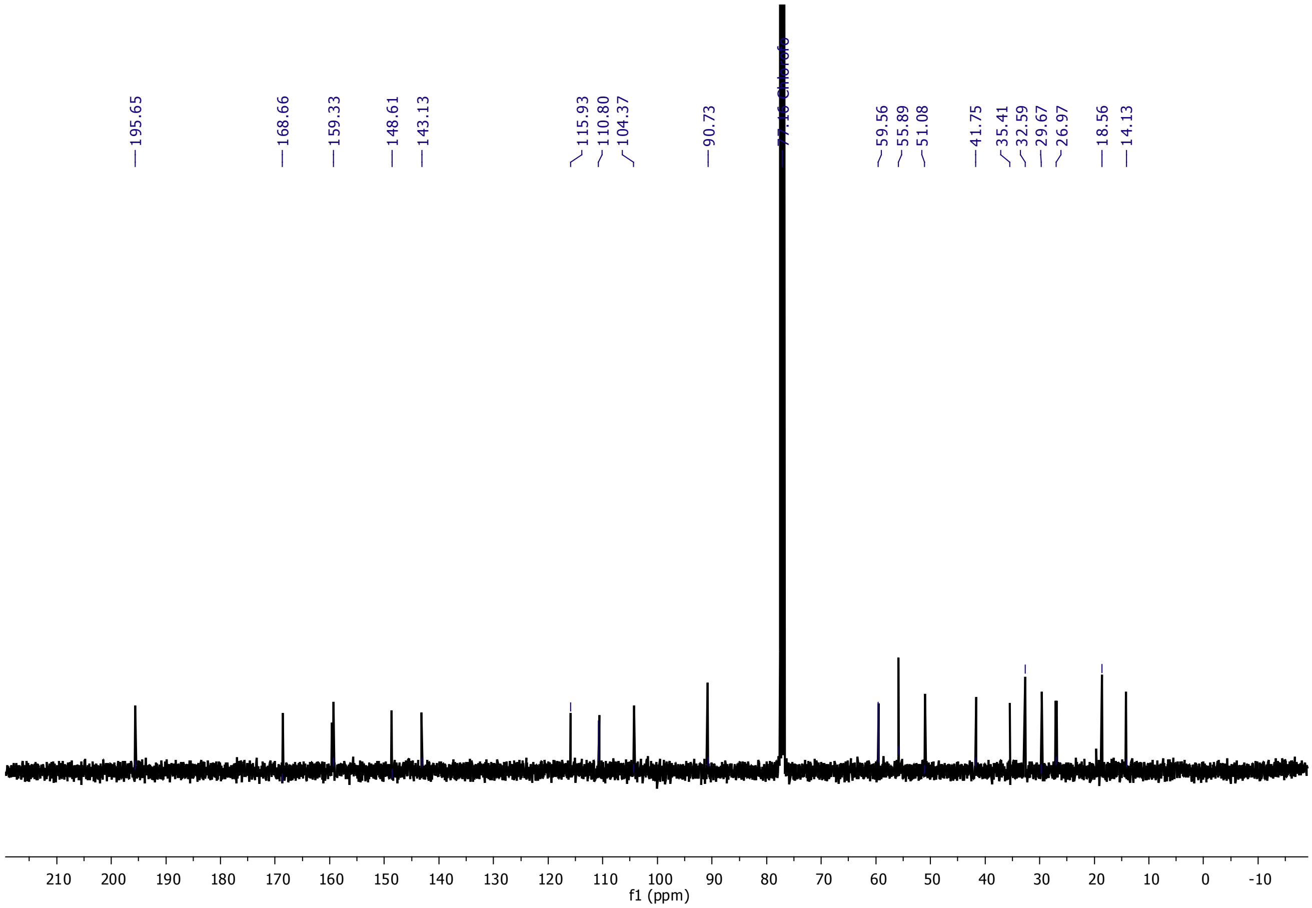

Figure S68.^{13}C NMR spectrum of compound **14** ($CDCl_3$, 100 MHz, TMS).

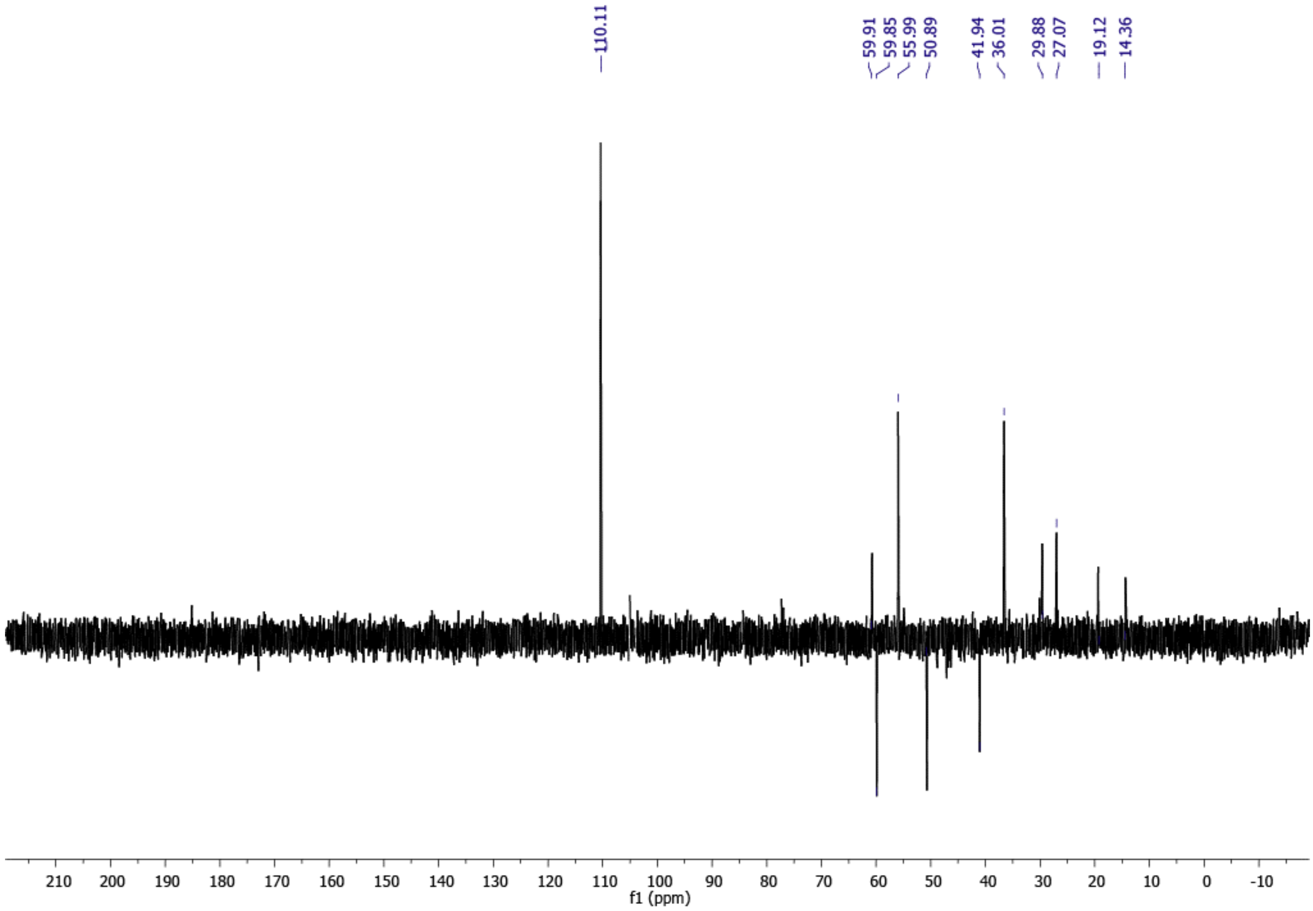

Figure S69. DEPT 135 spectrum of compound **14** ($CDCl_3$, 100 MHz, TMS).

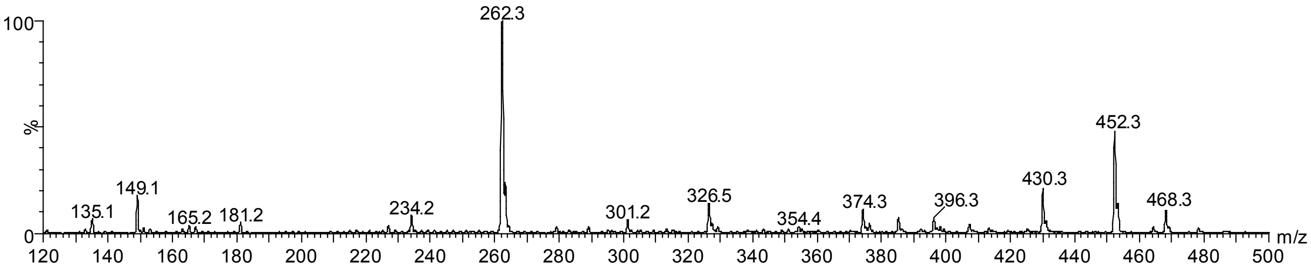

Figure S70. ESI (+) mass spectrum of compound **14.**

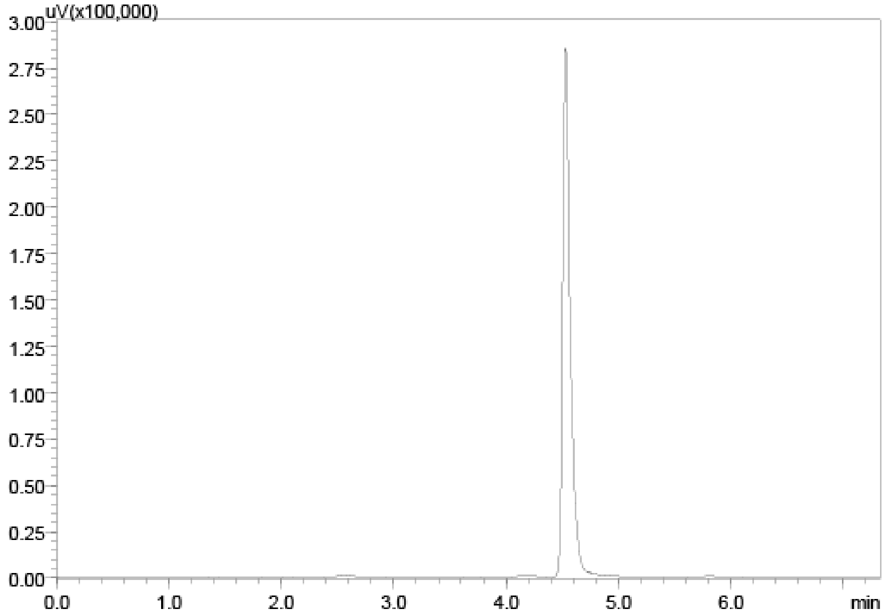

Figure S71: HPLC chromatogram of compound **15**.

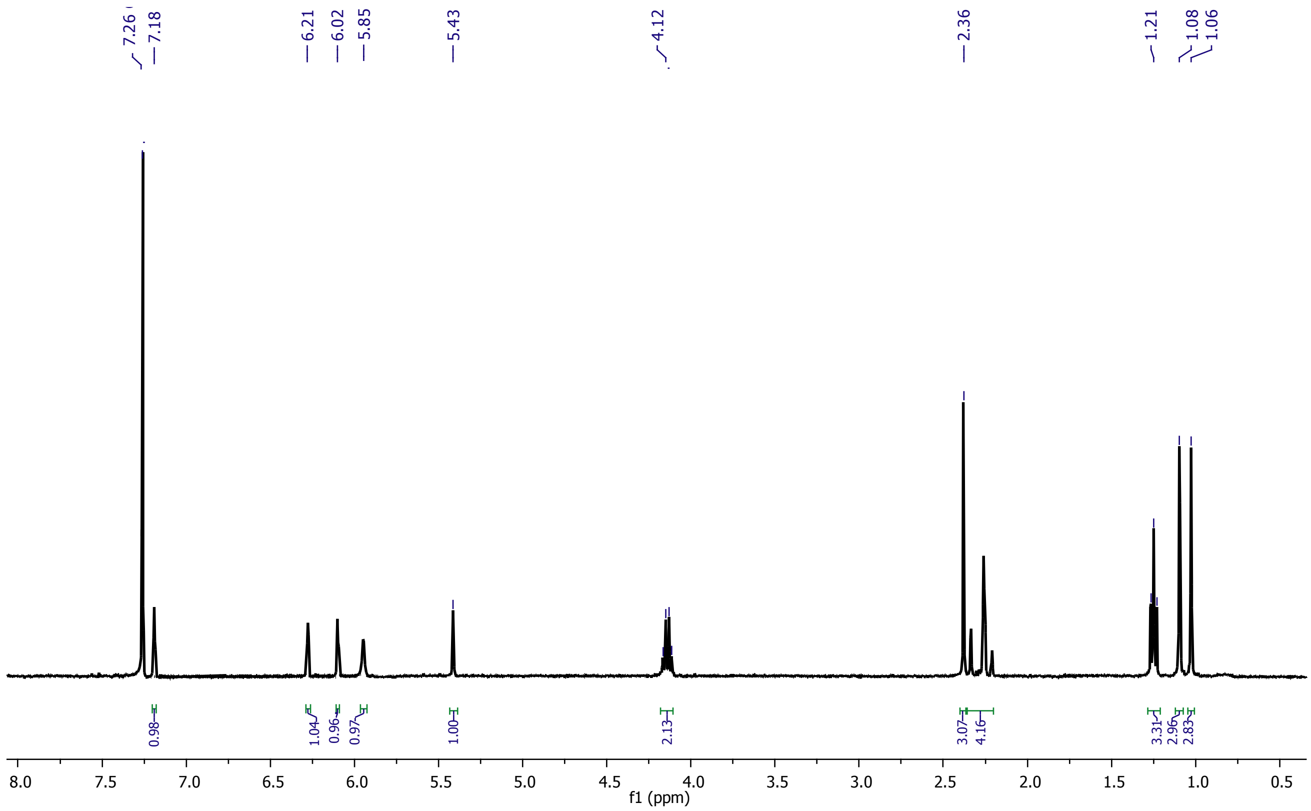

Figure S72.^{1}H NMR spectrum of compound **15** ($CDCl_3$, 400 MHz, TMS).

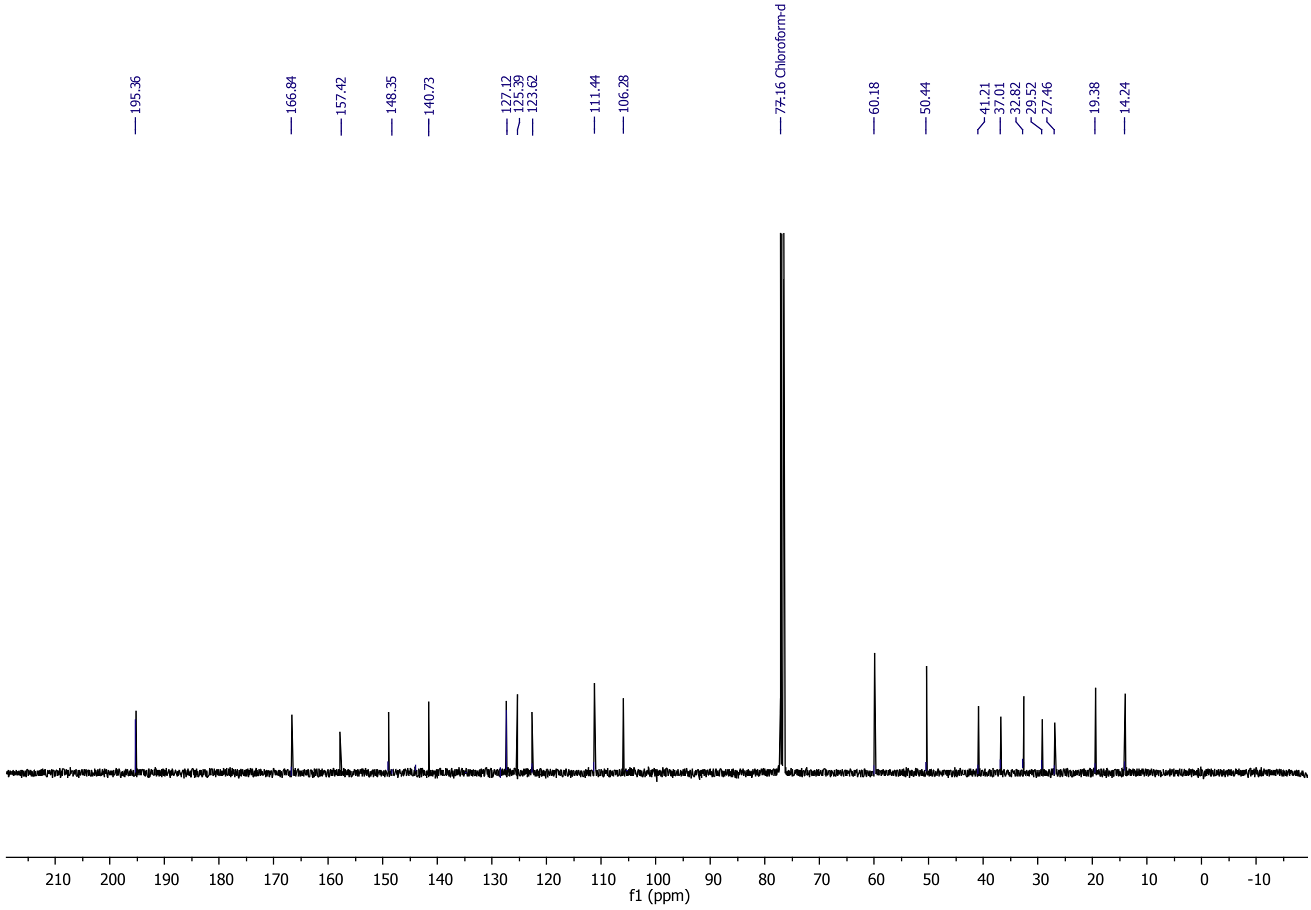

Figure S73.^{13}C NMR spectrum of compound **15**($CDCl_3$, 100 MHz, TMS).

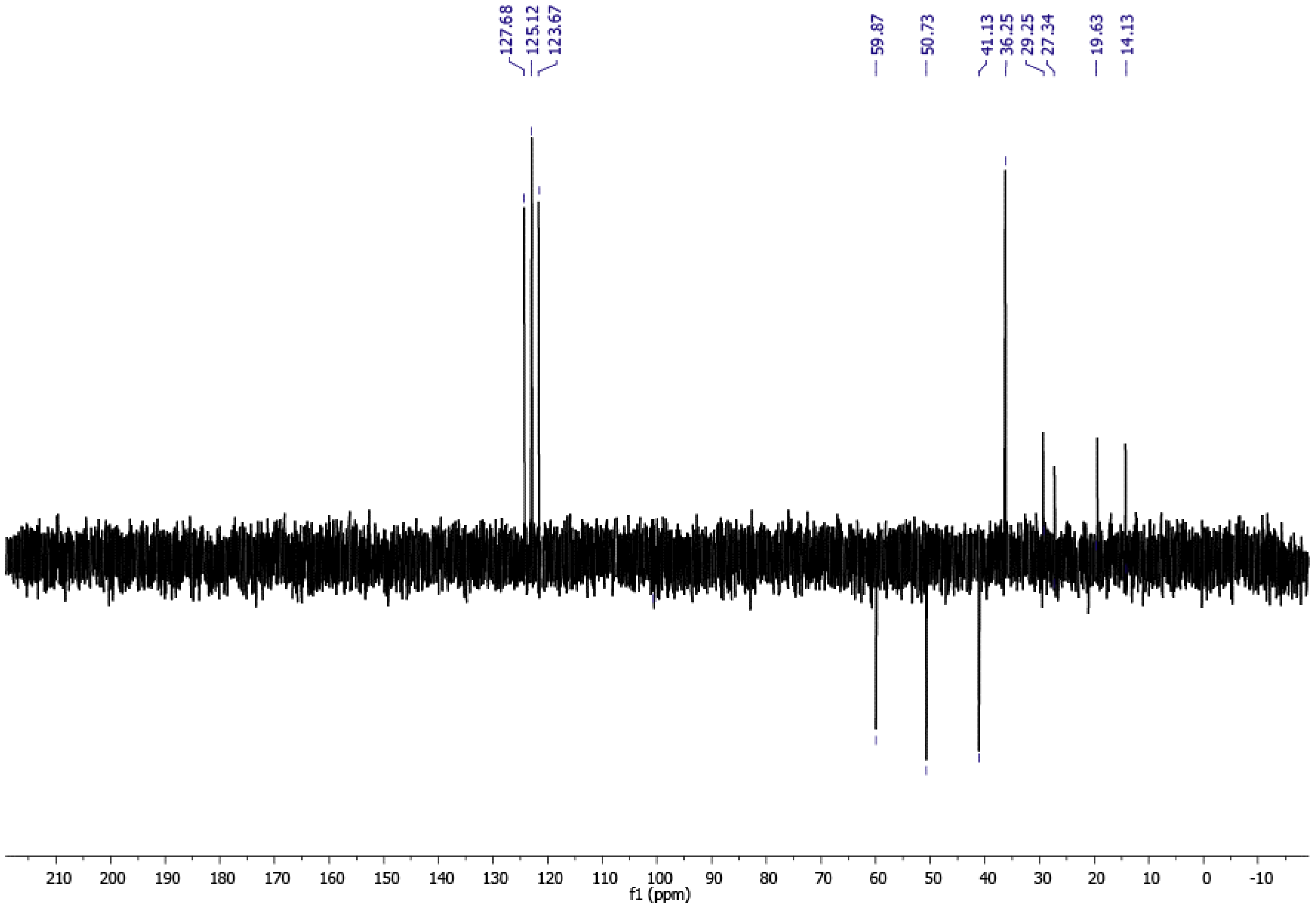

Figure S74. DEPT 135 spectrum of compound **15** ($CDCl_3$, 100 MHz, TMS).

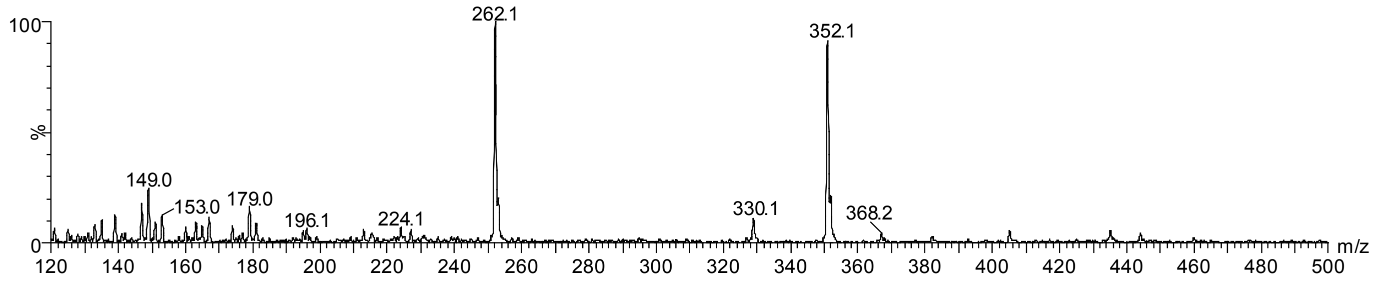

Figure S75. ESI (+) mass spectrum of compound **15.**

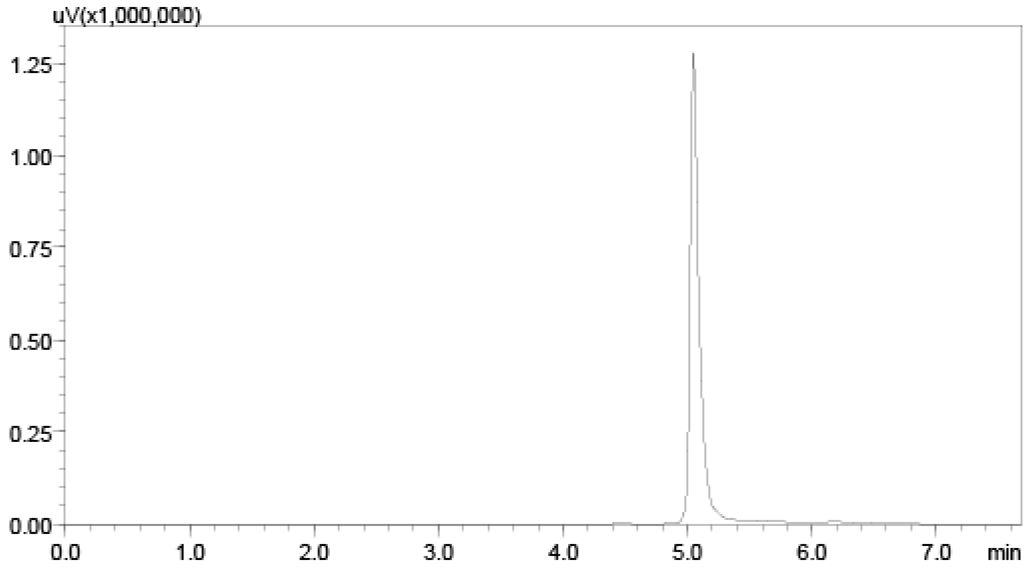

Figure S76: HPLC chromatogram of compound **16**.

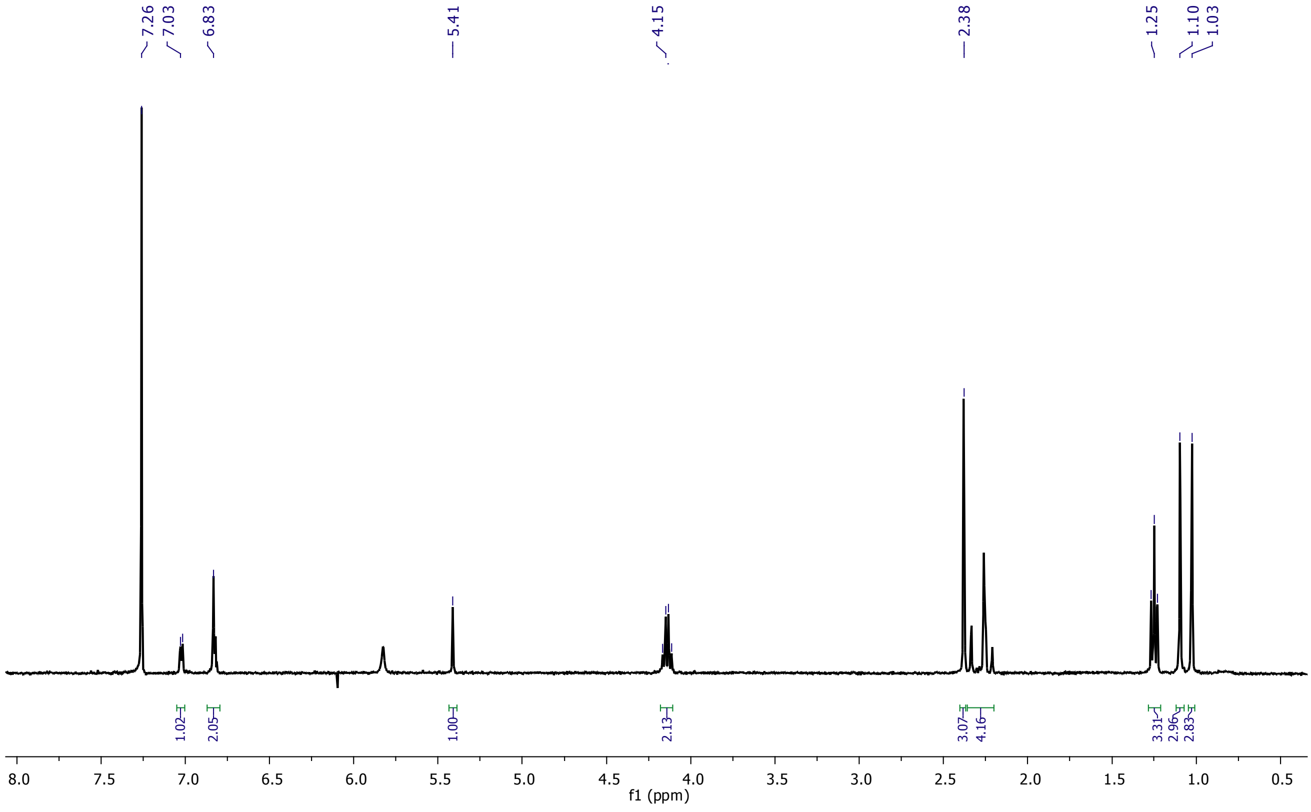

Figure S77.^{1}H NMR spectrum of compound **16** ($CDCl_3$, 400 MHz, TMS).

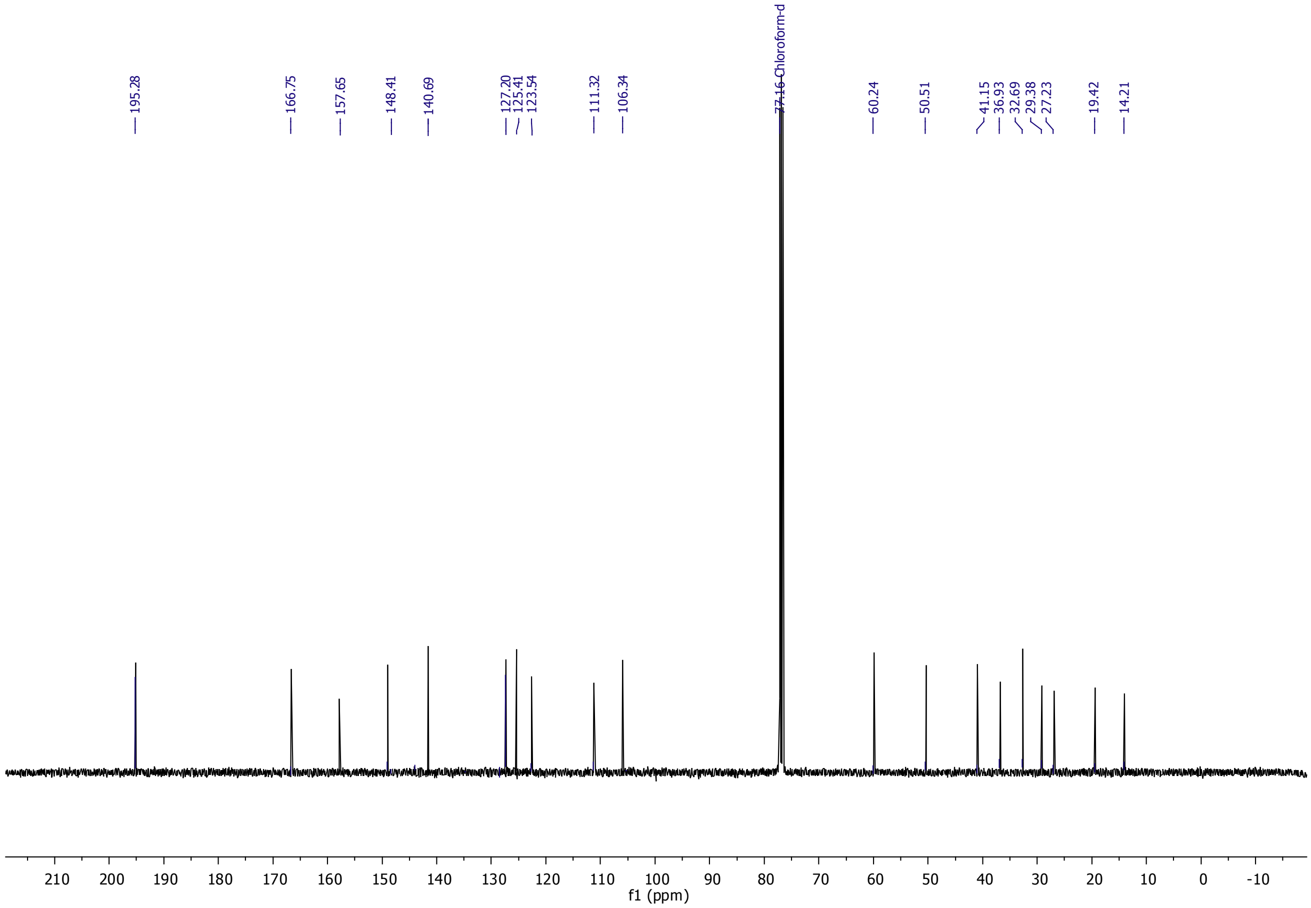

Figure S78.^{13}C NMR spectrum of compound **16** ($CDCl_3$, 100 MHz, TMS).

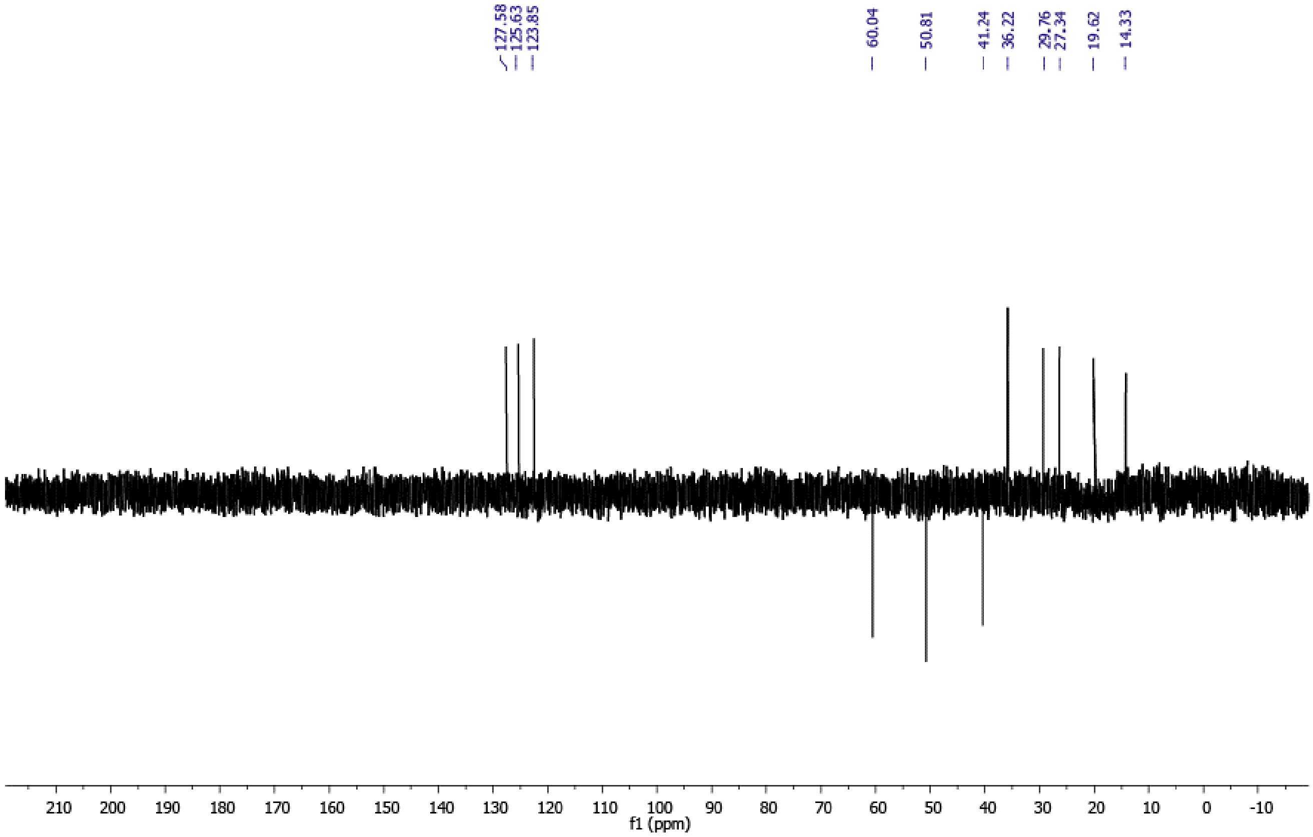

Figure S79. DEPT 135 spectrum of compound **16** ($CDCl_3$, 100 MHz, TMS).

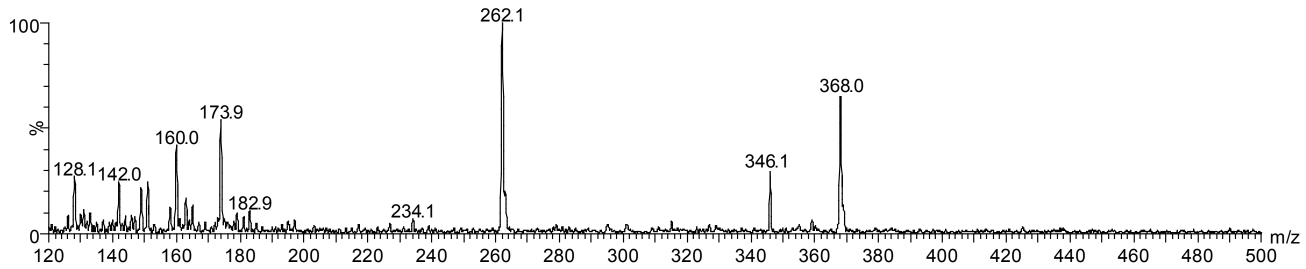

Figure S80. ESI (+) mass spectrum of compound **16.**

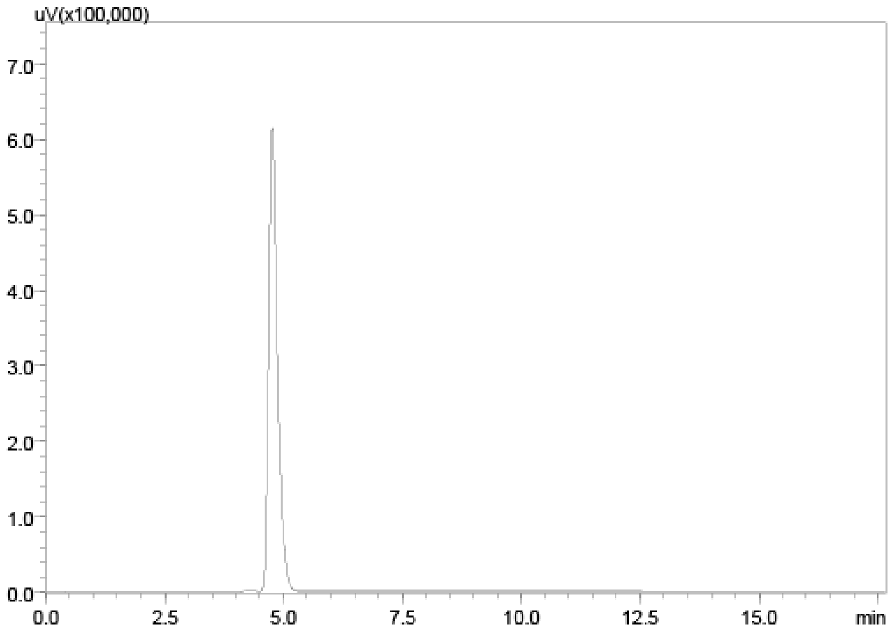

Figure S81: HPLC chromatogram of compound **17**

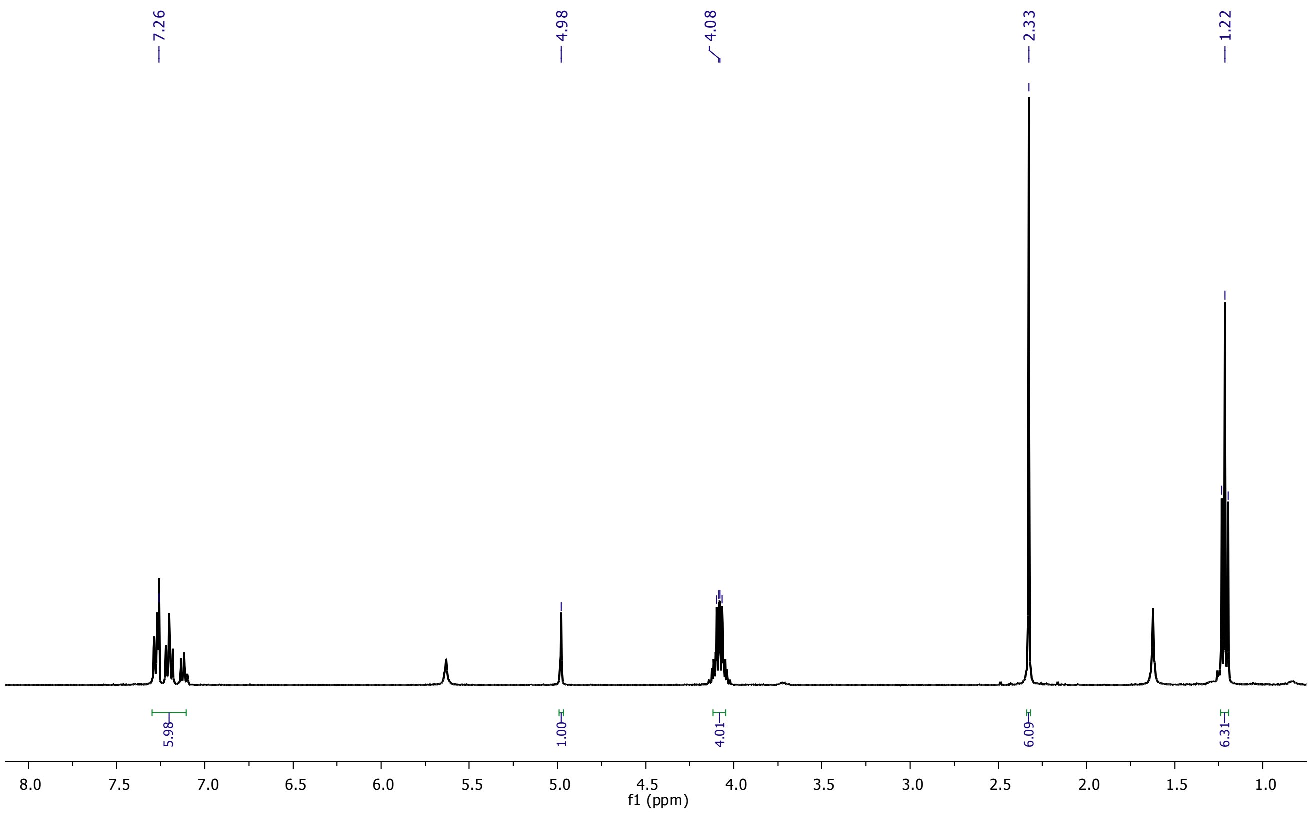

Figure S82.^{1}H NMR spectrum of compound **17** ($CDCl_3$, 400 MHz, TMS).

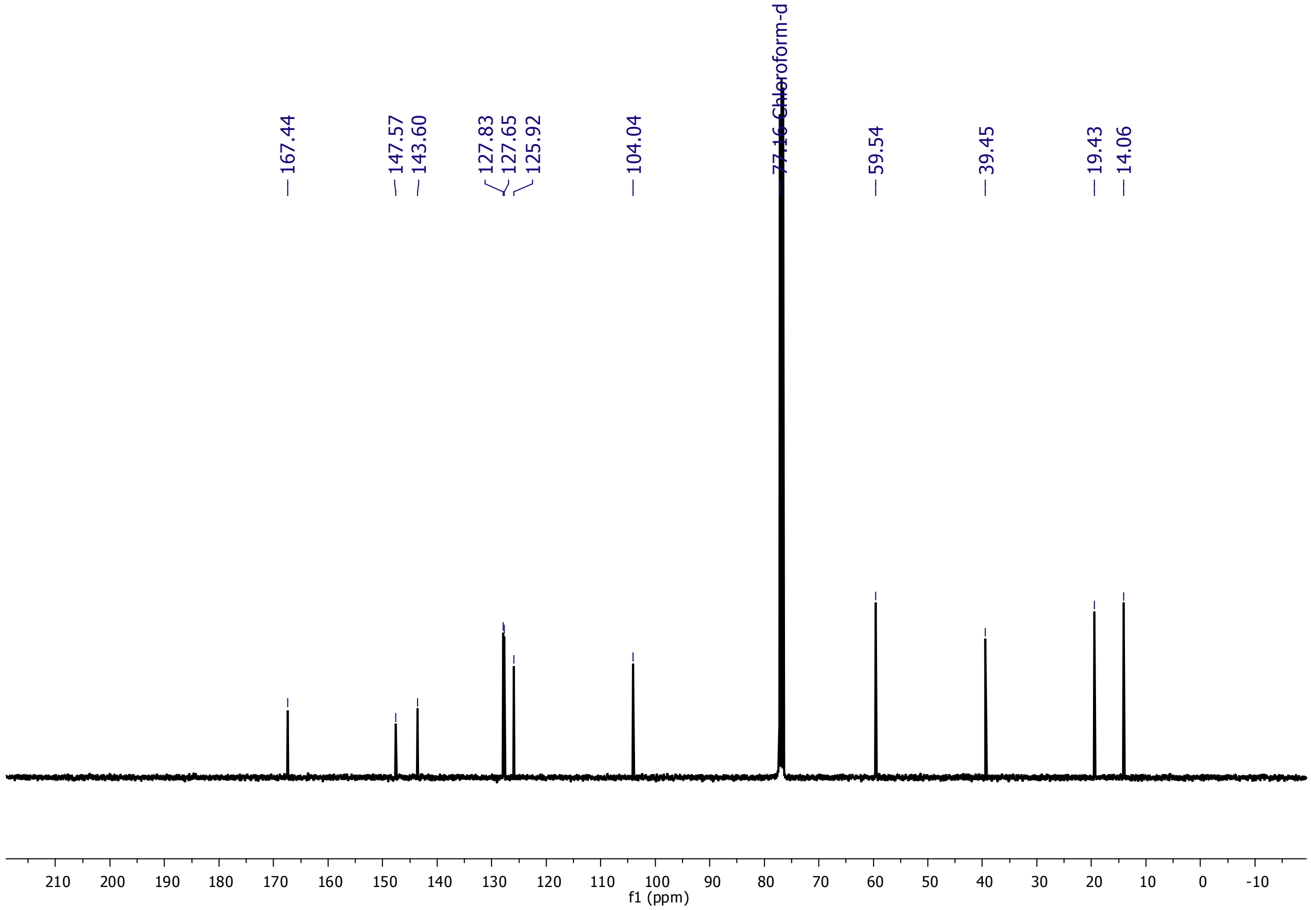

Figure S83.^{13}C NMR spectrum of compound **17** ($CDCl_3$, 100 MHz, TMS).

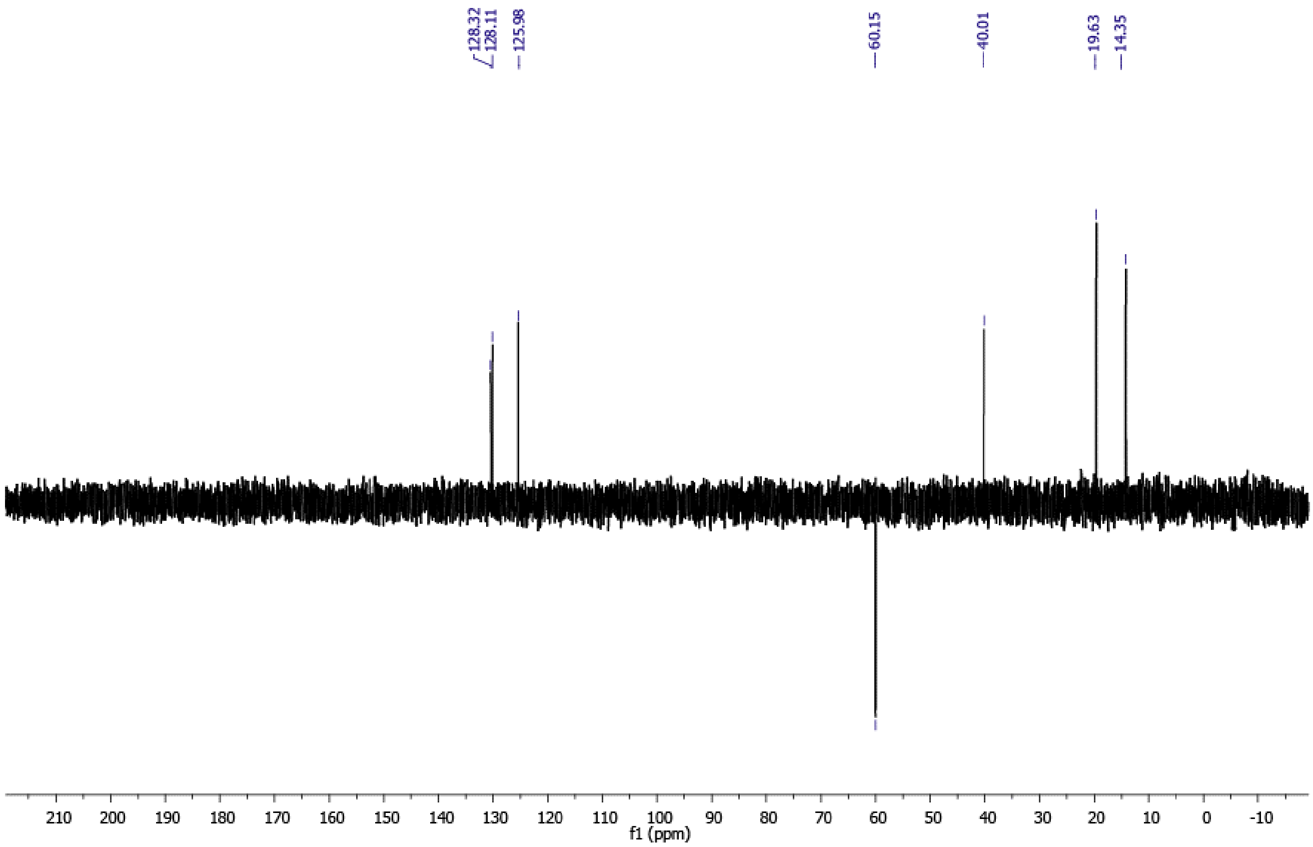

Figure S84. DEPT 135 spectrum of compound **17** ($CDCl_3$, 100 MHz, TMS).

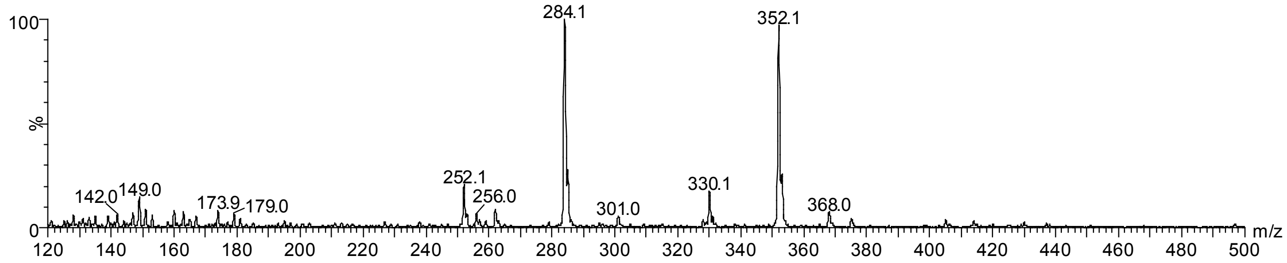

Figure S85. ESI (+) mass spectrum of compound **17.**

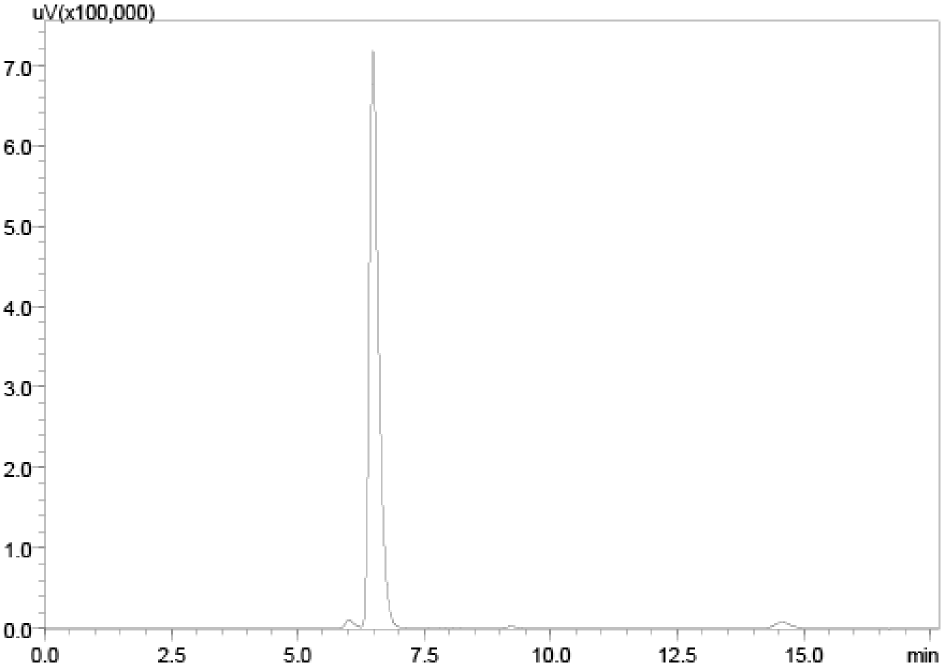

Figure S86: HPLC chromatogram of compound **18**.

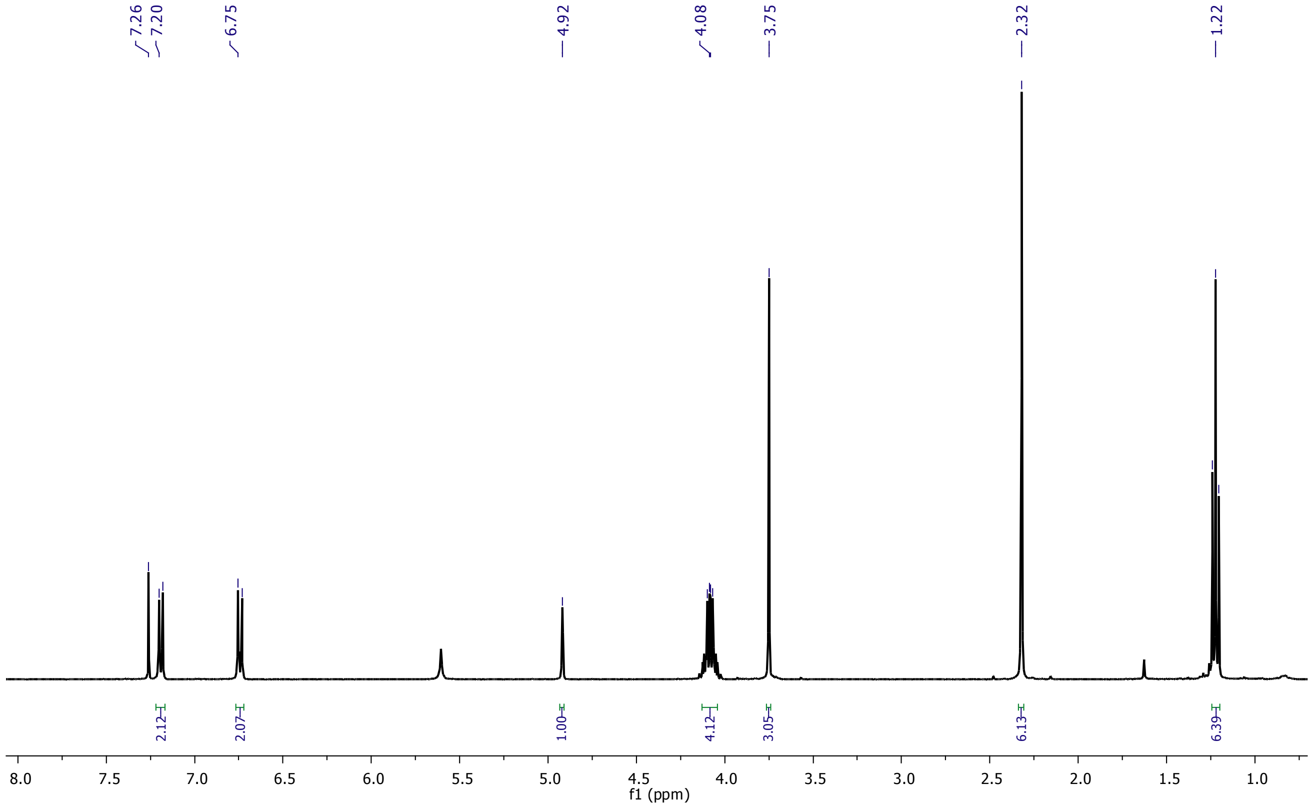

Figure S87.^{1}H NMR spectrum of compound **18** ($CDCl_3$, 400 MHz, TMS).

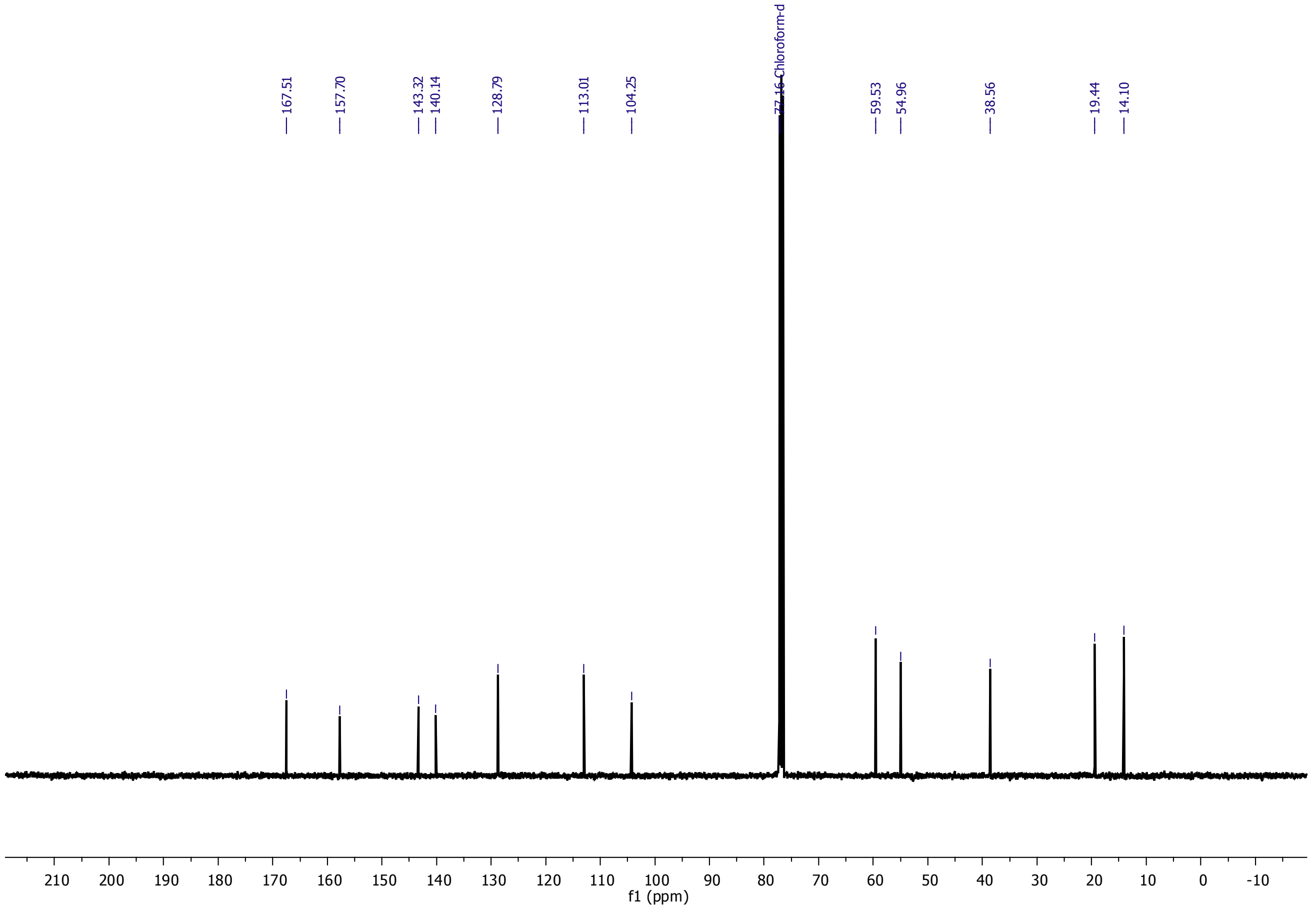

Figure S88.^{13}C NMR spectrum of compound **18** ($CDCl_3$, 100 MHz, TMS).

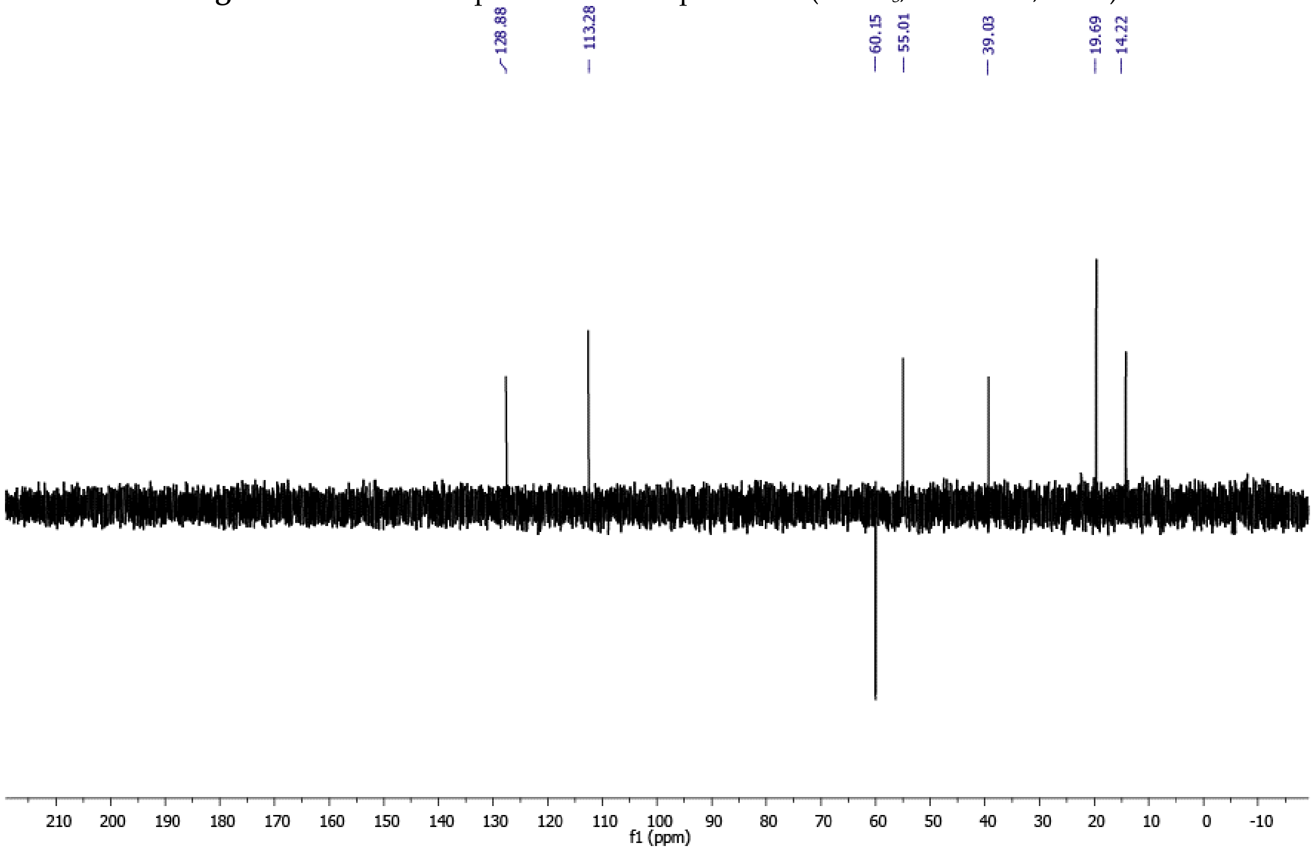

Figure S89. DEPT 135 spectrum of compound **18** ($CDCl_3$, 100 MHz, TMS).

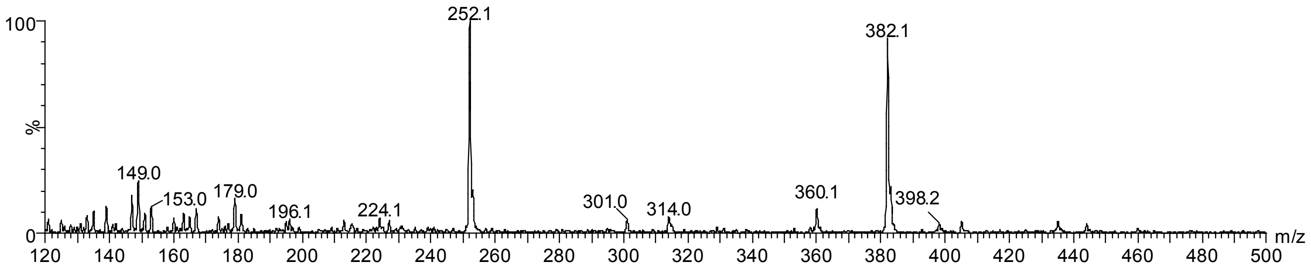

Figure S90. ESI (+) mass spectrum of compound **18.**

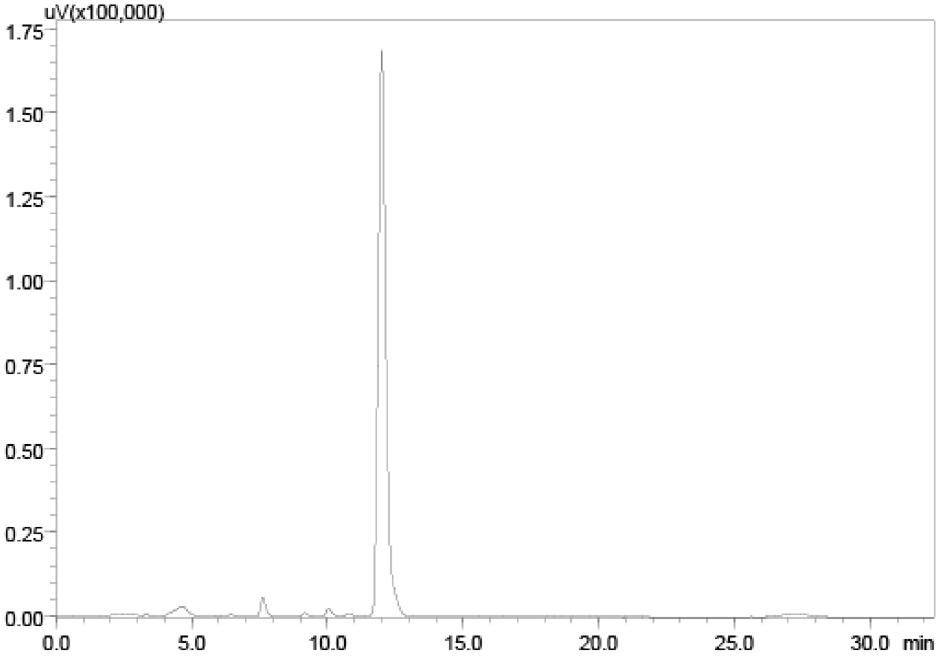

Figure S91: HPLC chromatogram of compound **19**.

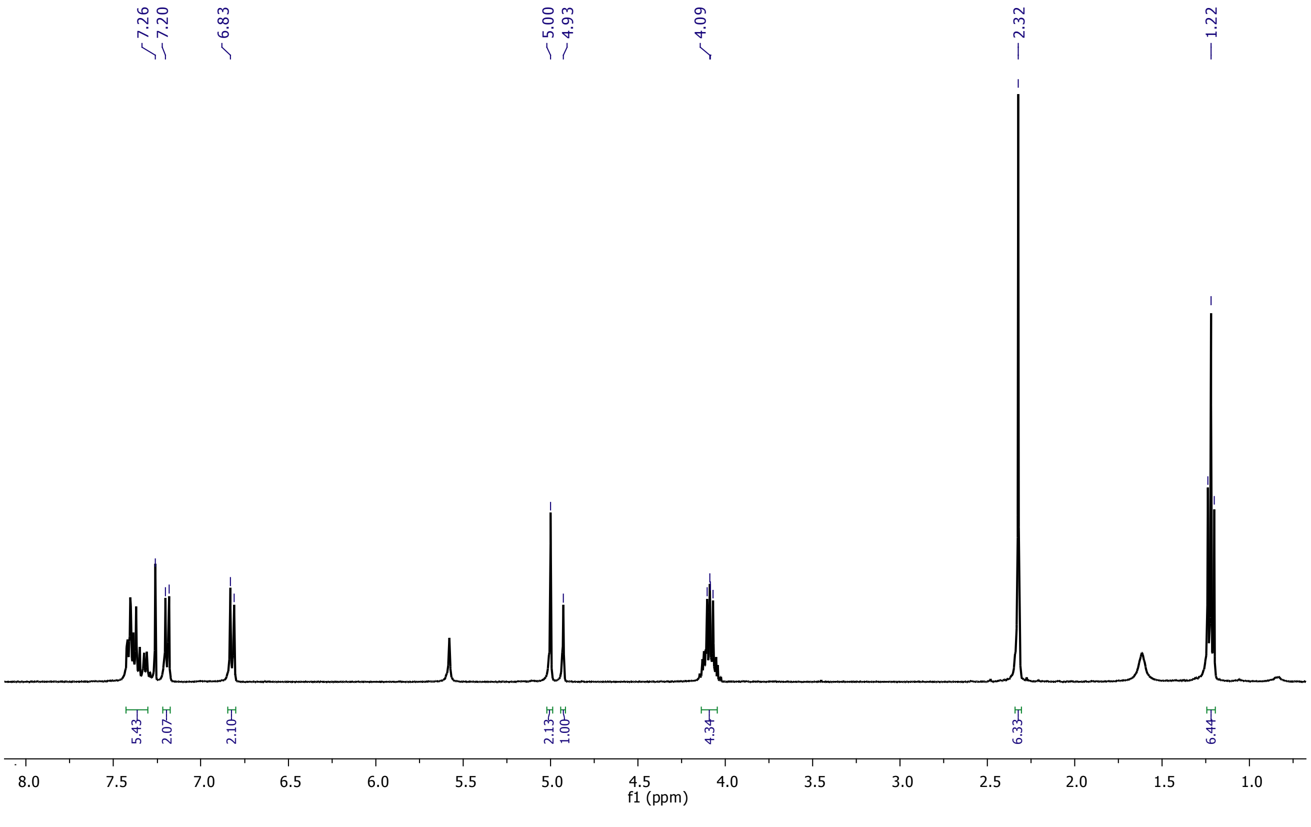

Figure S92.^{1}H NMR spectrum of compound **19** ($CDCl_3$, 400 MHz, TMS).

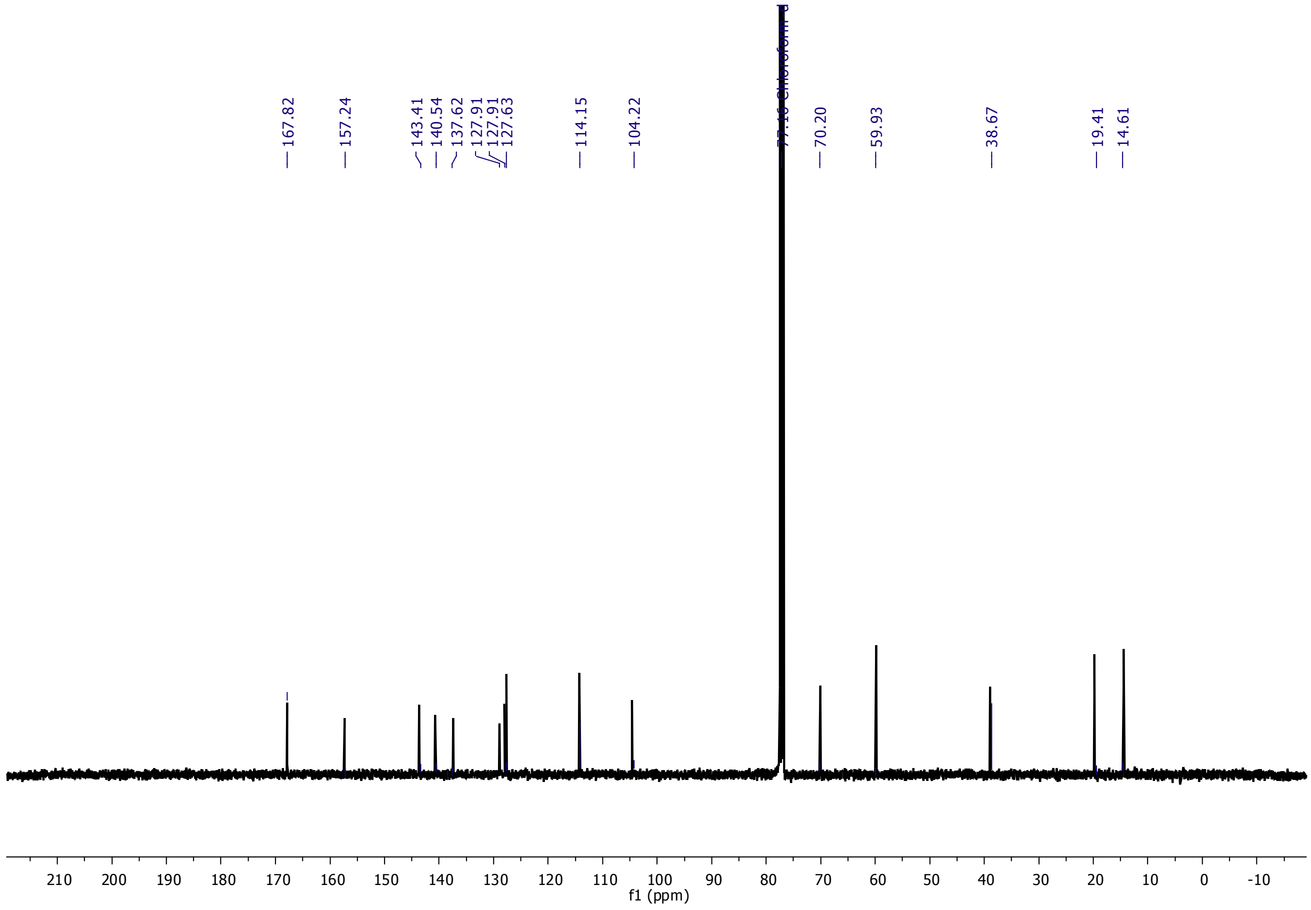

Figure S93.^{13}C NMR spectrum of compound **19** ($CDCl_3$, 100 MHz, TMS).

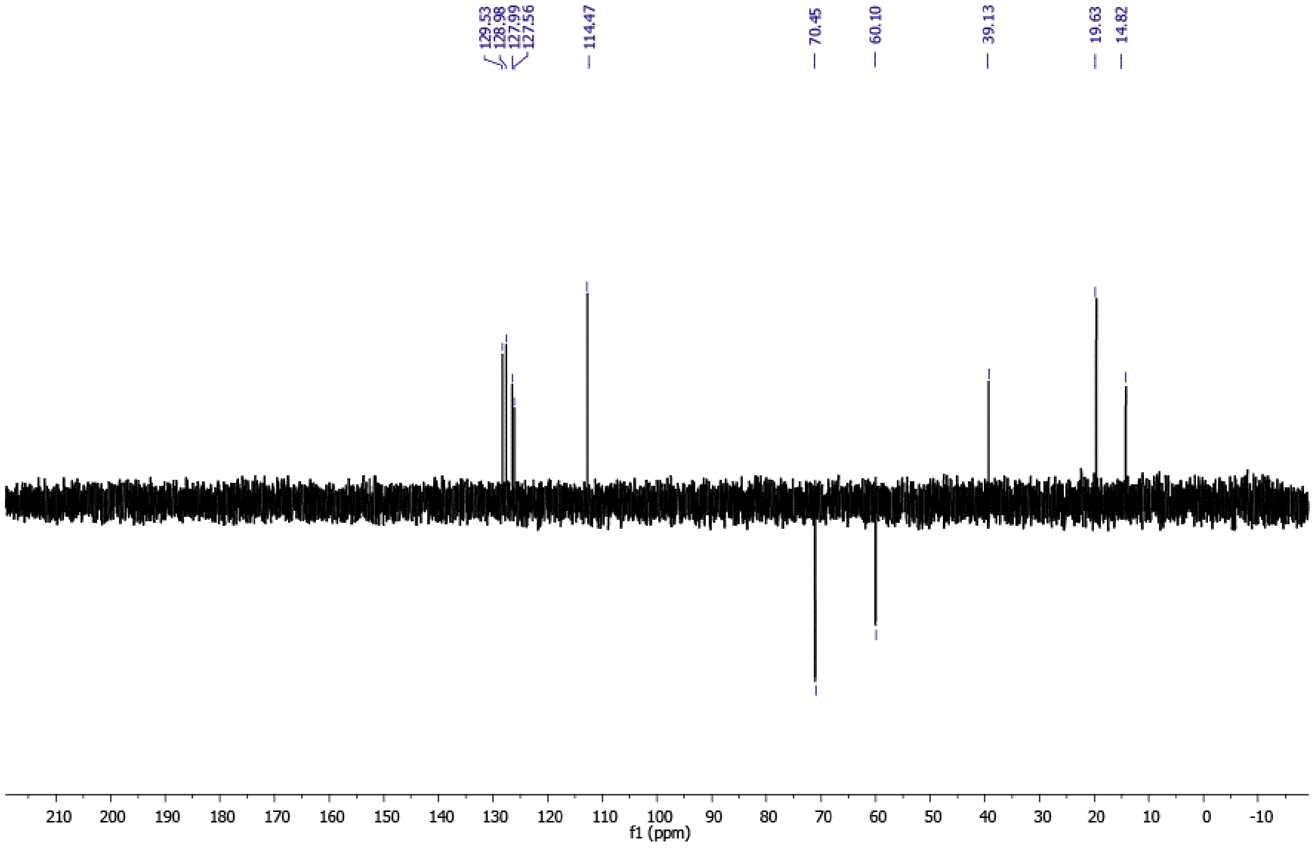

Figure S94. DEPT 135 spectrum of compound **19** ($CDCl_3$, 100 MHz, TMS).

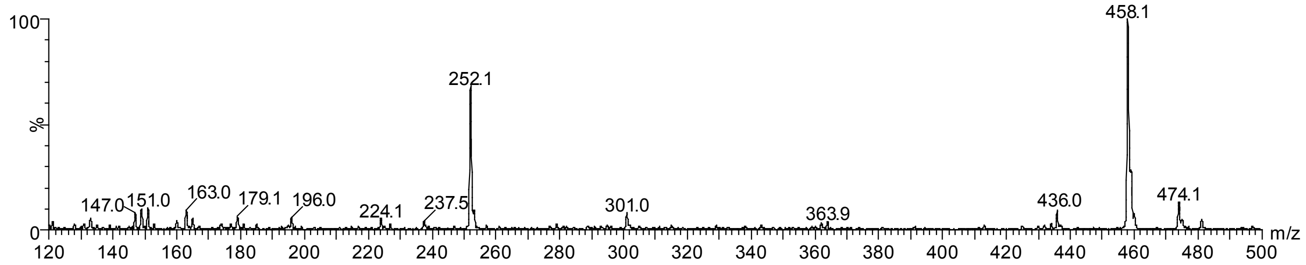

Figure S95. ESI (+) mass spectrum of compound **19.**

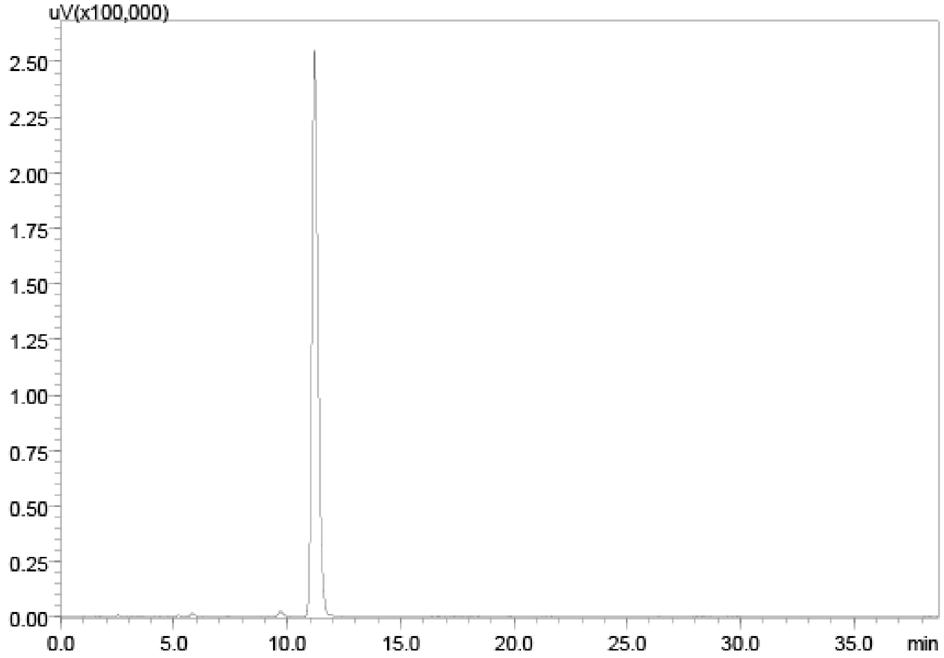

Figure S96: HPLC chromatogram of compound **20**.

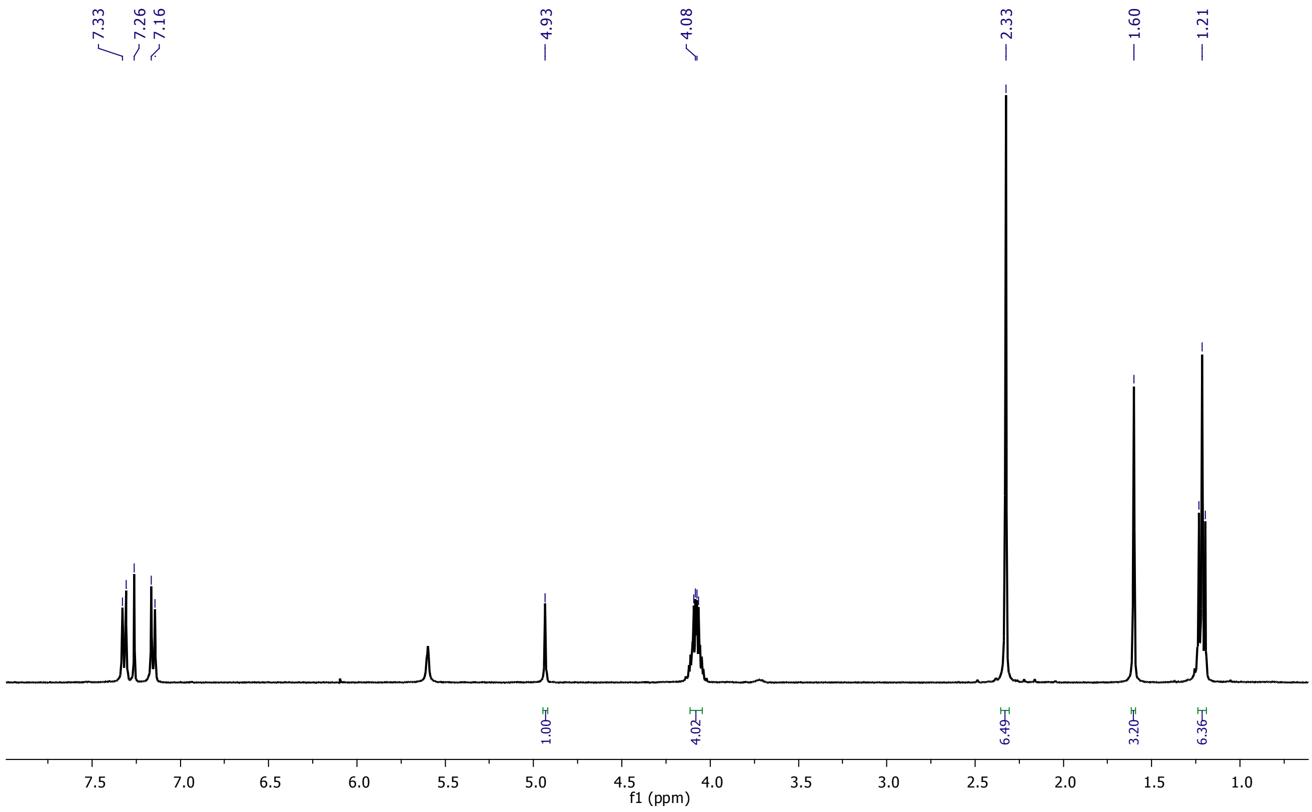

Figure S97.^{1}H NMR spectrum of compound **20** ($CDCl_3$, 400 MHz, TMS).

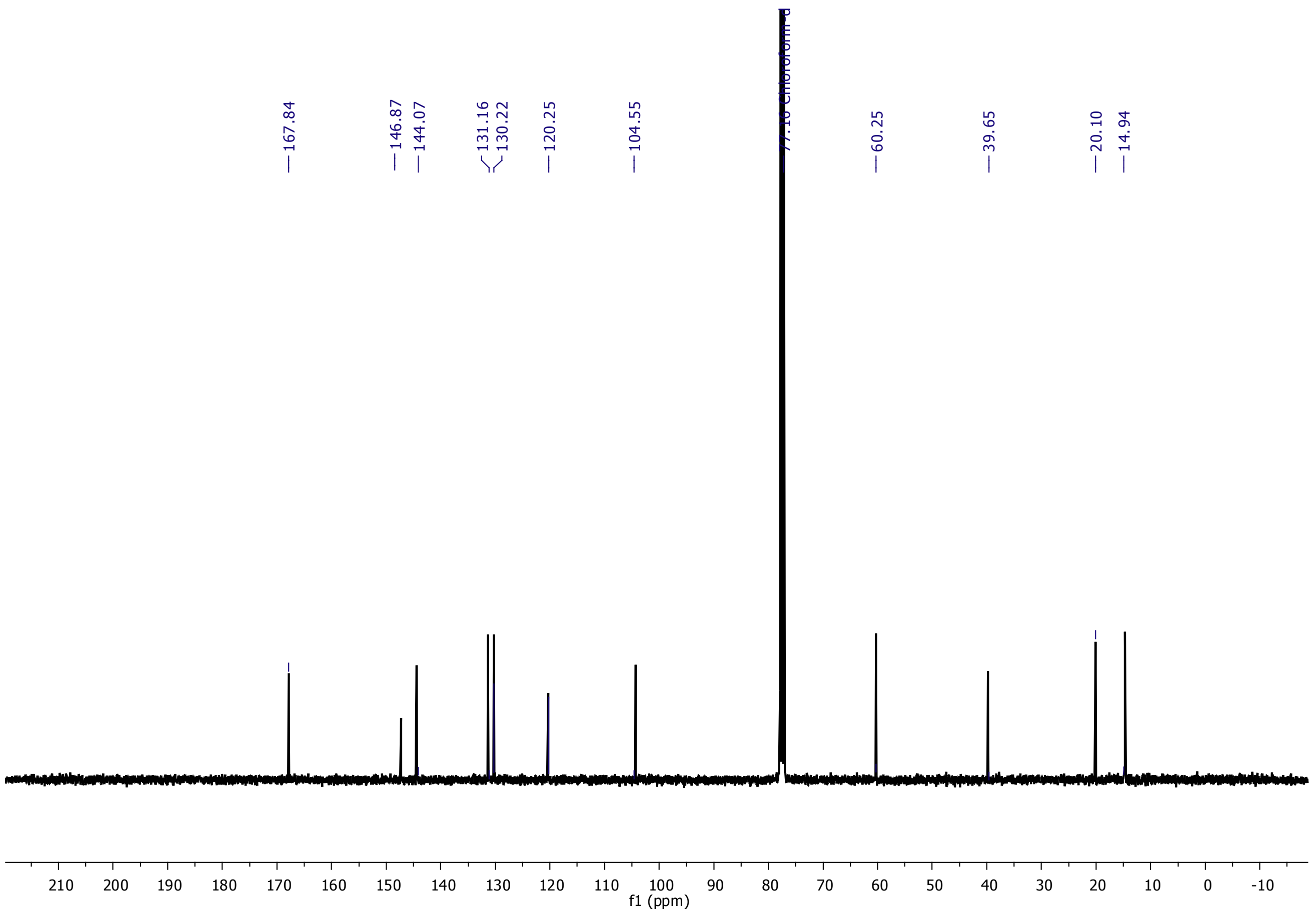

Figure S98.^{13}C NMR spectrum of compound **20** ($CDCl_3$, 100 MHz, TMS).

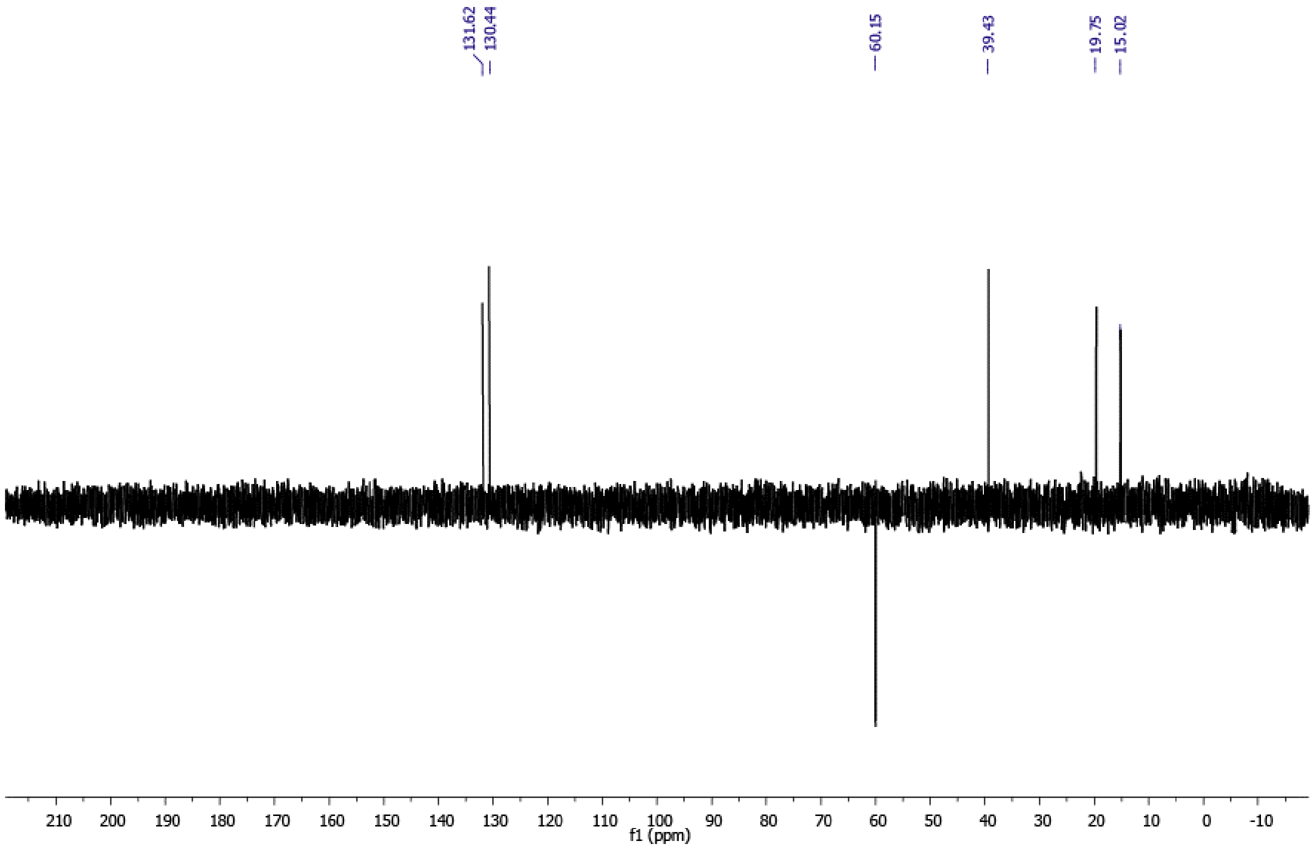

Figure S99. DEPT 135 spectrum of compound **20** ($CDCl_3$, 100 MHz, TMS).

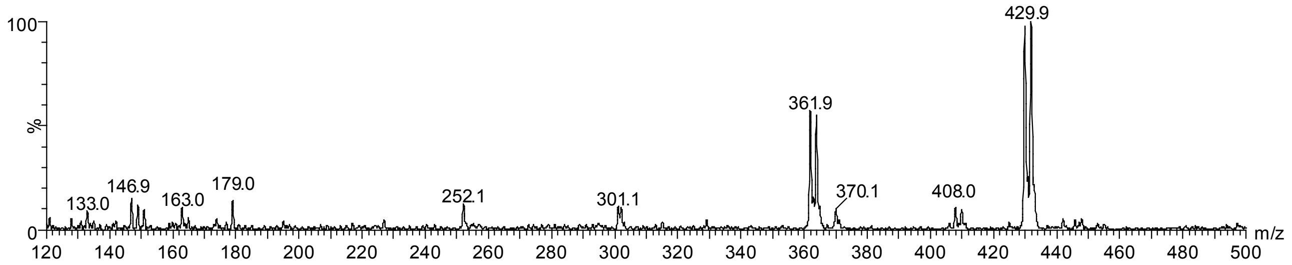

Figure S100. ESI (+) mass spectrum of compound **20.**

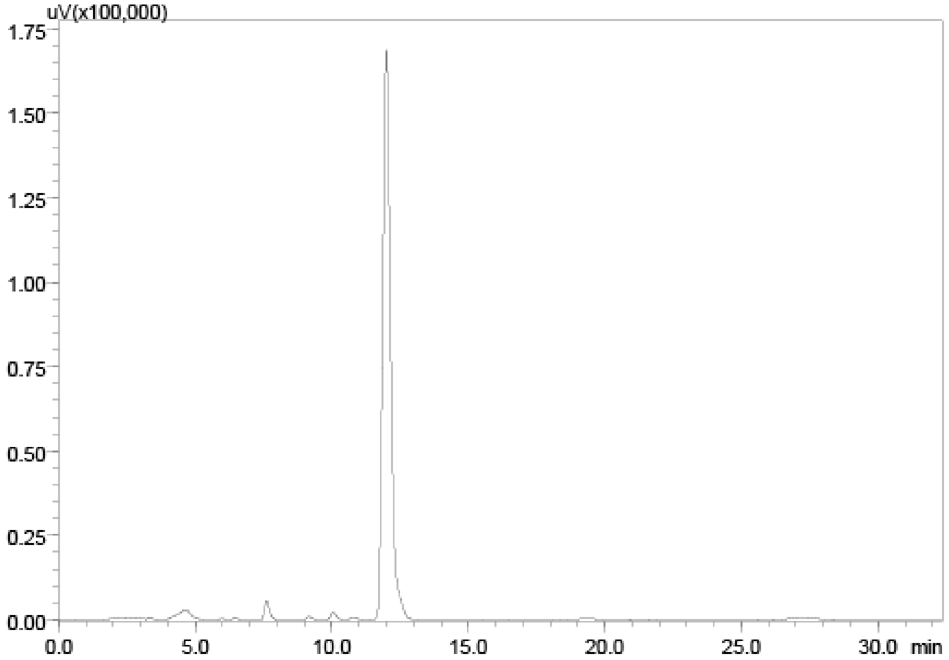

Figure S101: HPLC chromatogram of compound **21**.

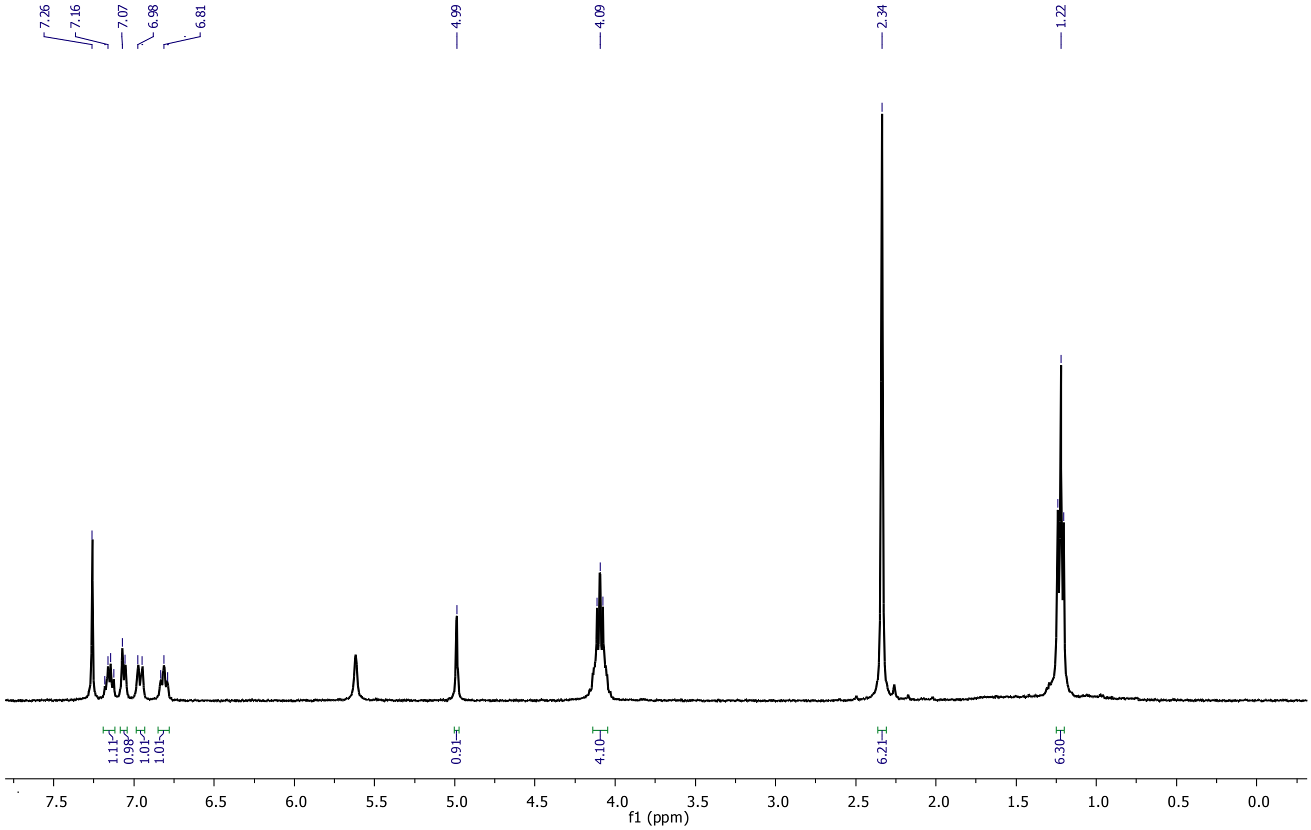

Figure S102.^{1}H NMR spectrum of compound **21** ($CDCl_3$, 400 MHz, TMS).

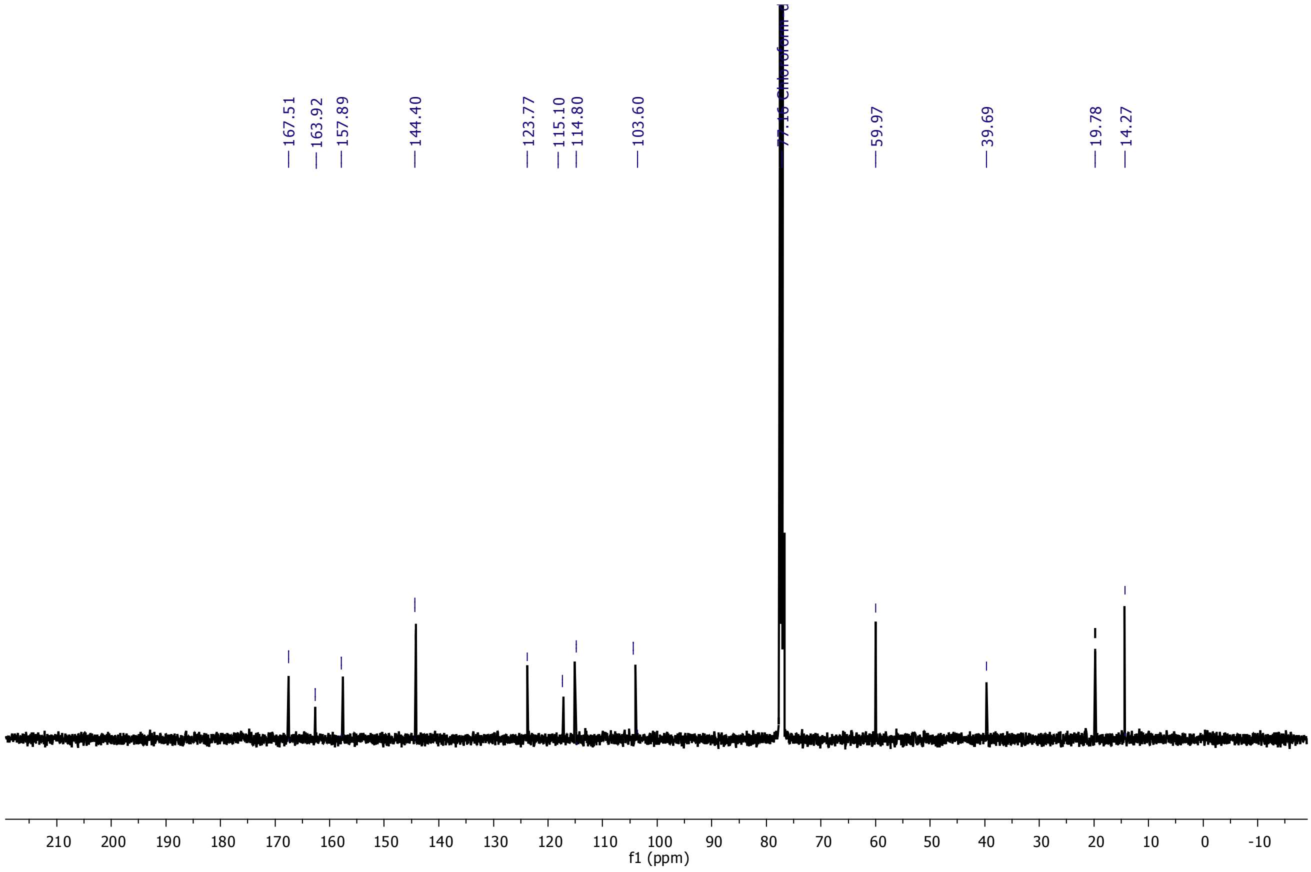

Figure S103.^{13}C NMR spectrum of compound **21** ($CDCl_3$, 100 MHz, TMS).

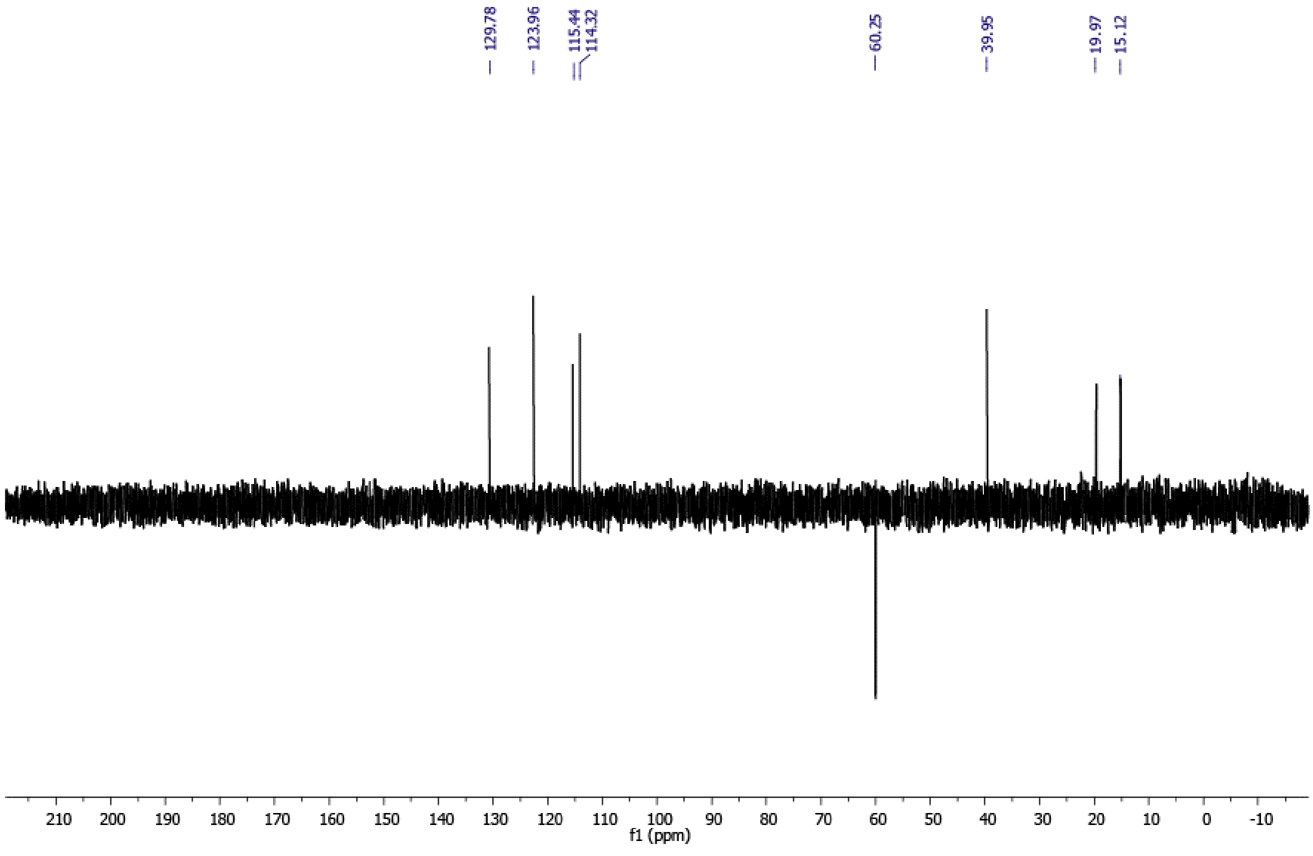

Figure S104. DEPT 135 spectrum of compound **21** ($CDCl_3$, 100 MHz, TMS).

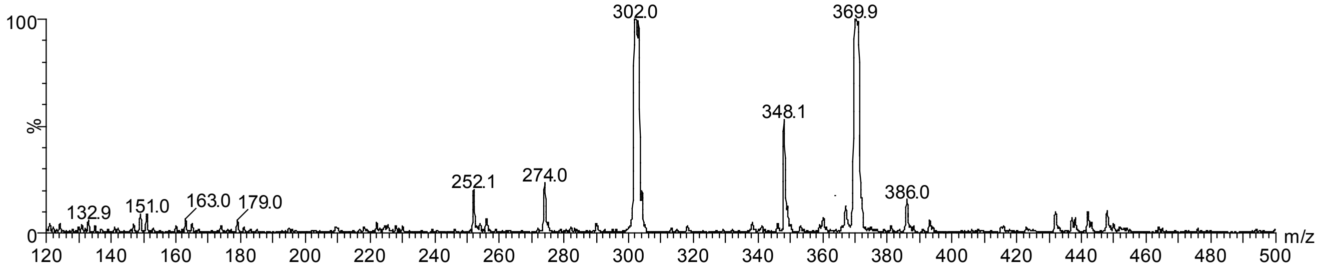

Figure S105. ESI (+) mass spectrum of compound **21.**

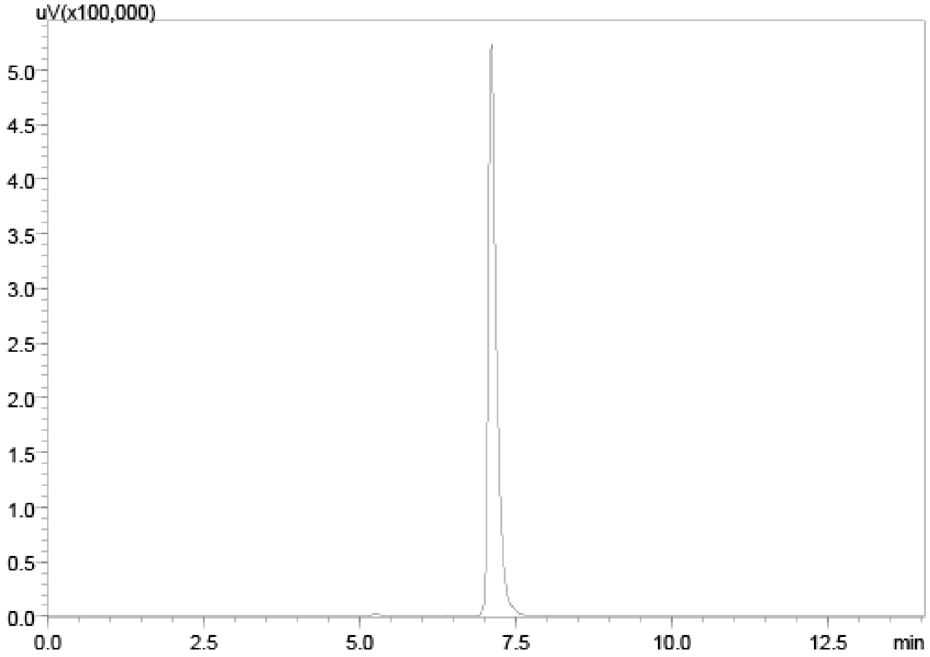

Figure S106: HPLC chromatogram of compound **22**.

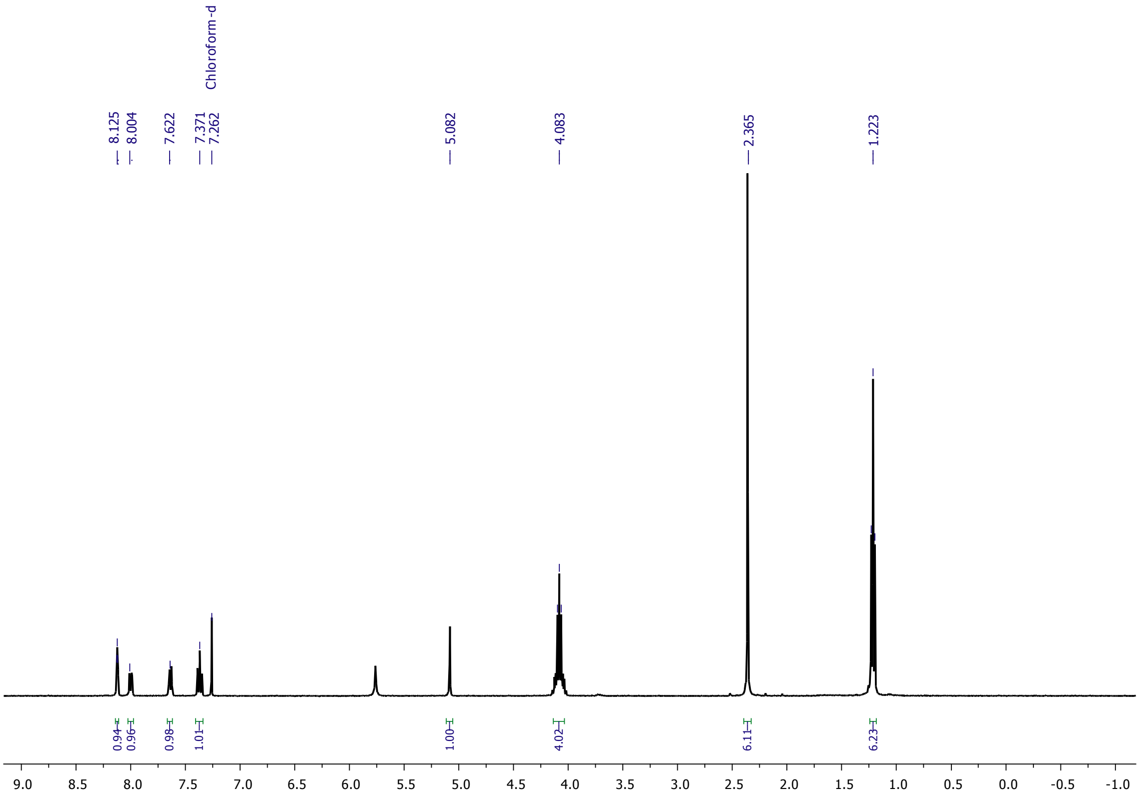

Figure S107.^{1}H NMR spectrum of compound **22** ($CDCl_3$, 400 MHz, TMS).

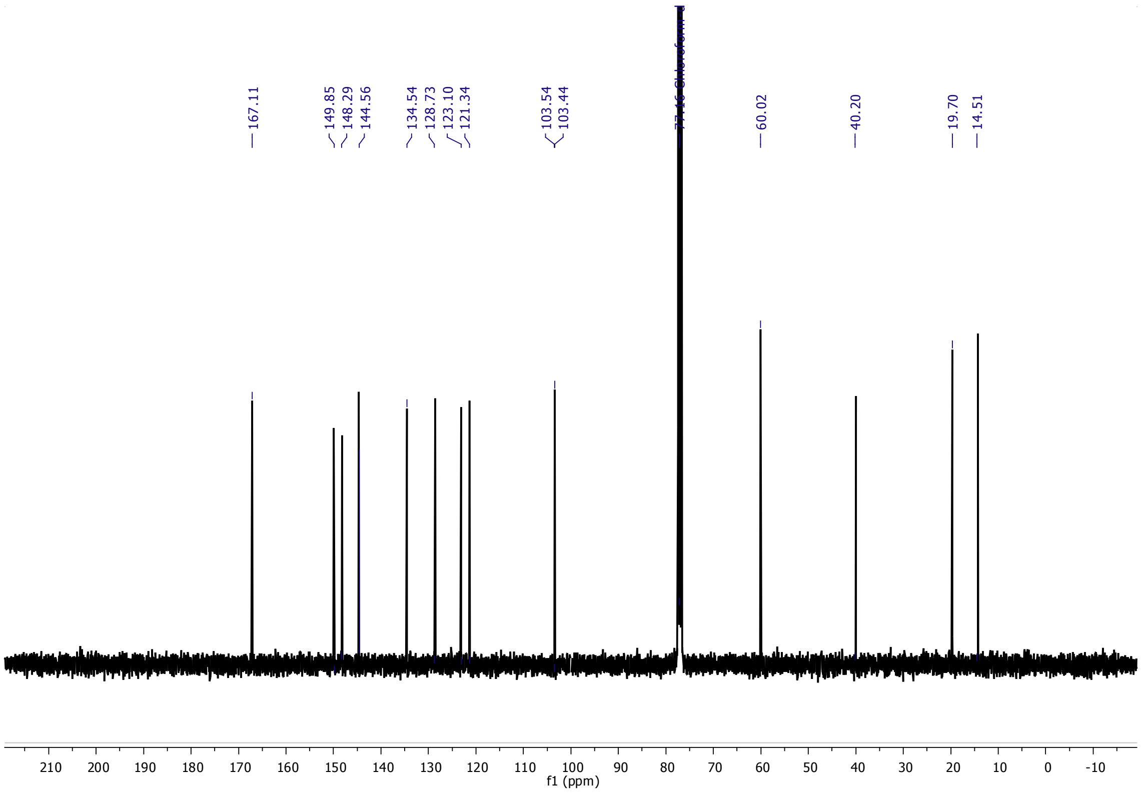

Figure S108.^{13}C NMR spectrum of compound **22** ($CDCl_3$, 100 MHz, TMS).

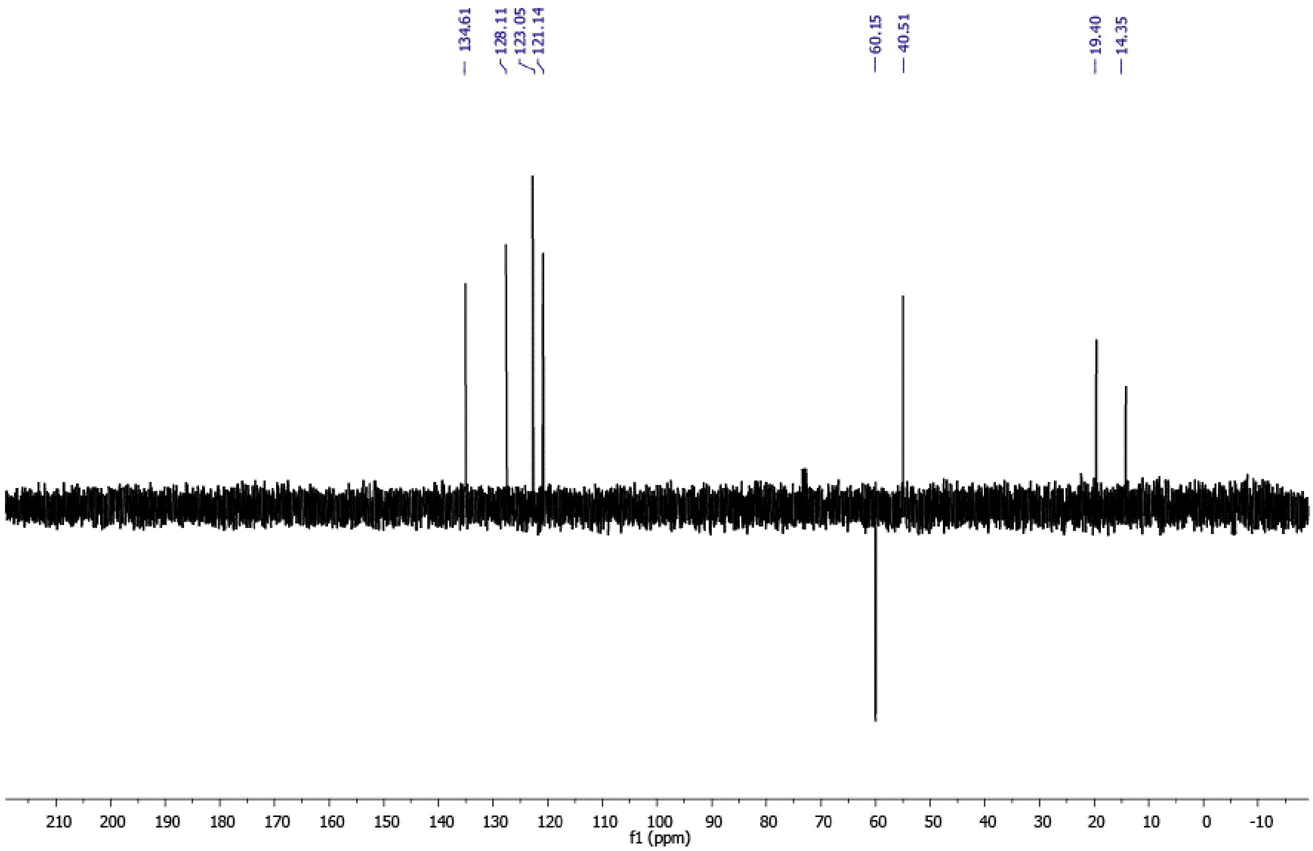

Figure S109. DEPT 135 spectrum of compound **22** ($CDCl_3$, 100 MHz, TMS).

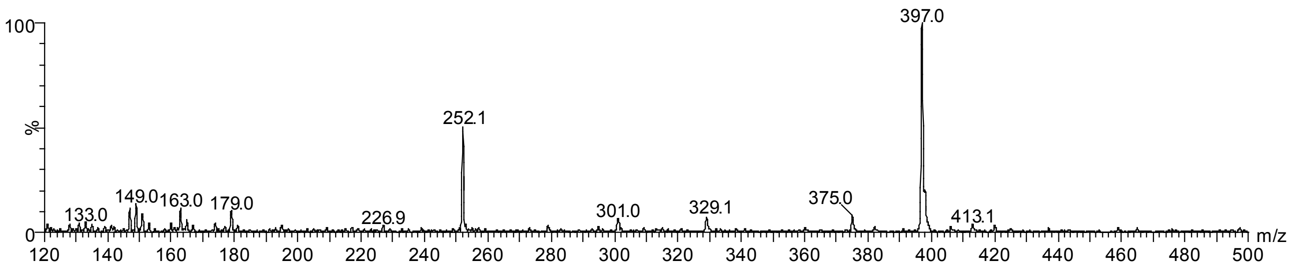

Figure S110. ESI (+) mass spectrum of compound **22.**

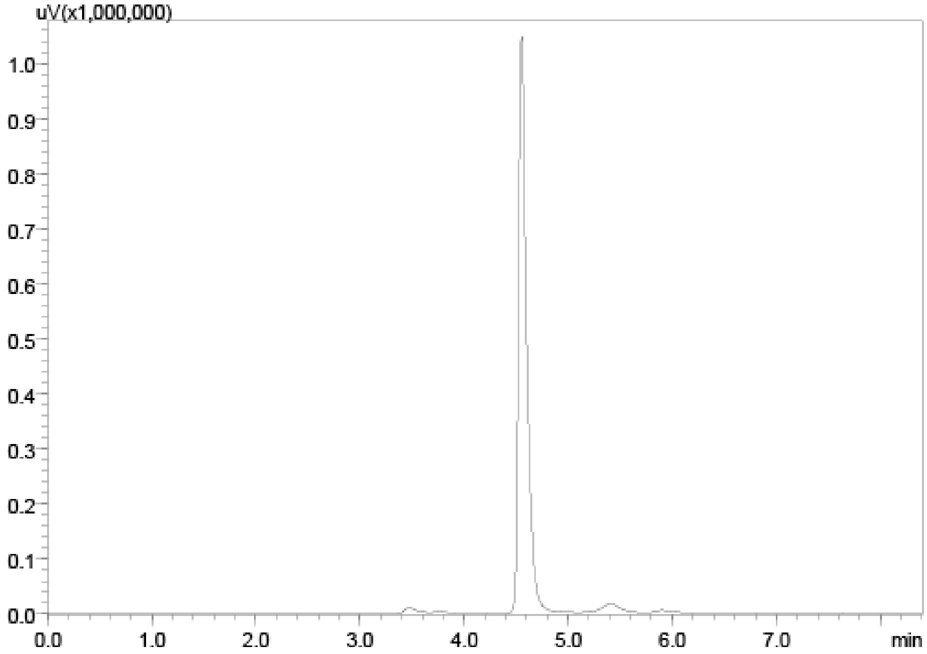

Figure S111: HPLC chromatogram of compound **23**.

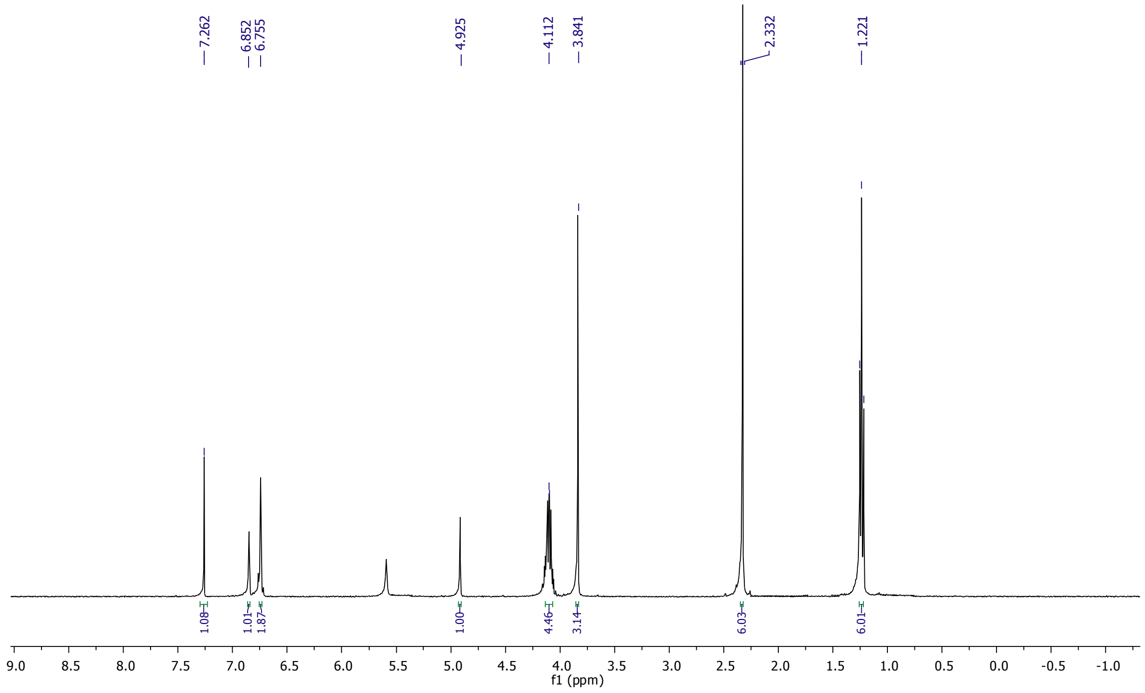

Figur112. ^{1}H NMR spectrum of compound **23** ($CDCl_3$, 400 MHz, TMS).

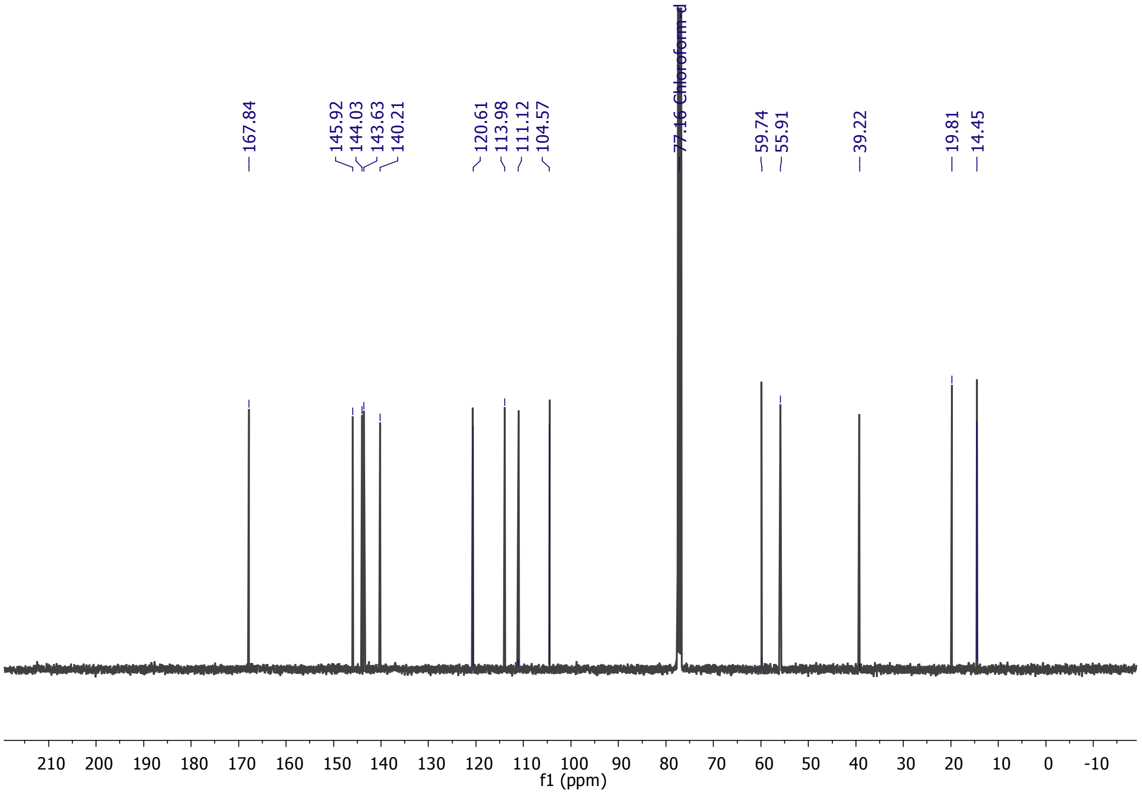

Figure S113.^{13}C NMR spectrum of compound **23** ($CDCl_3$, 100 MHz, TMS).

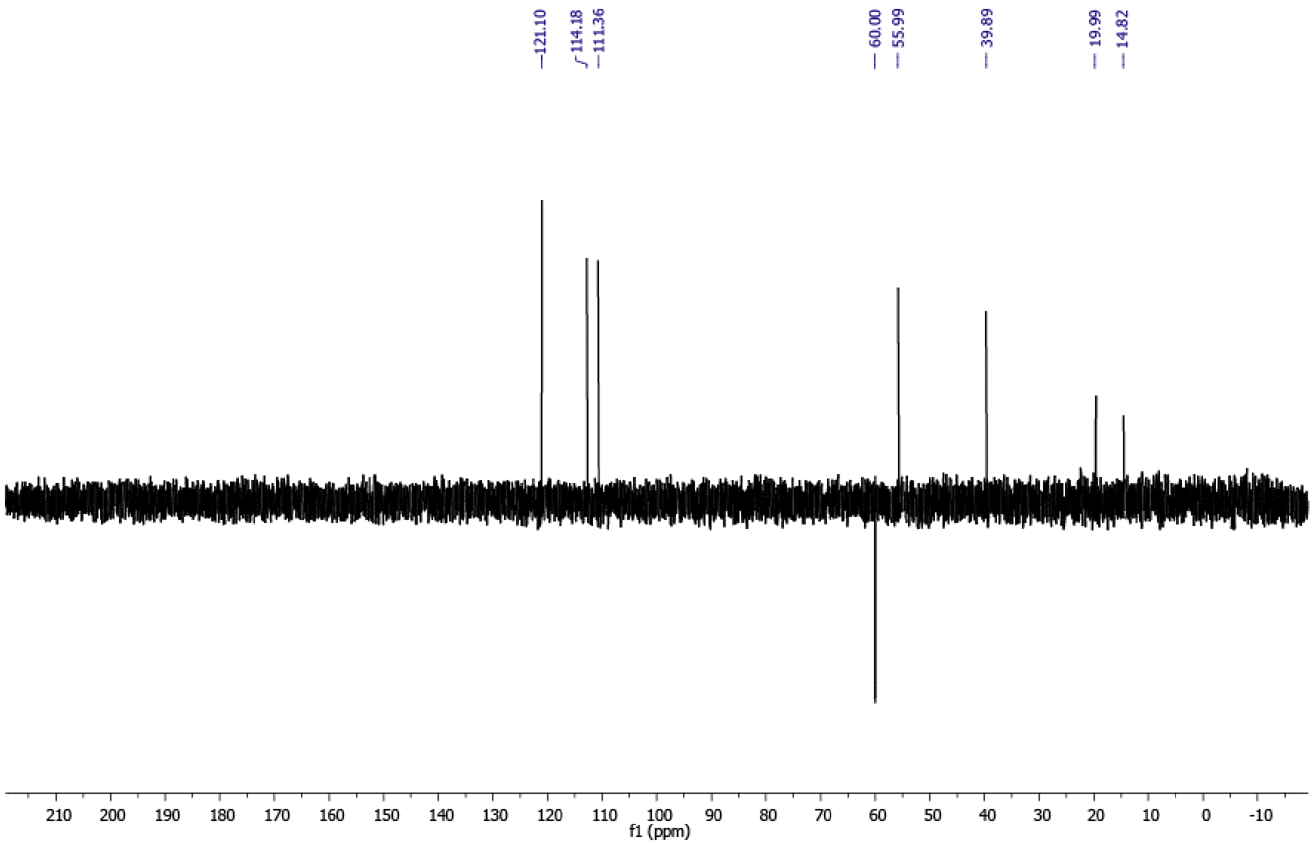

Figure S114. DEPT 135 spectrum of compound **23** ($CDCl_3$, 100 MHz, TMS).

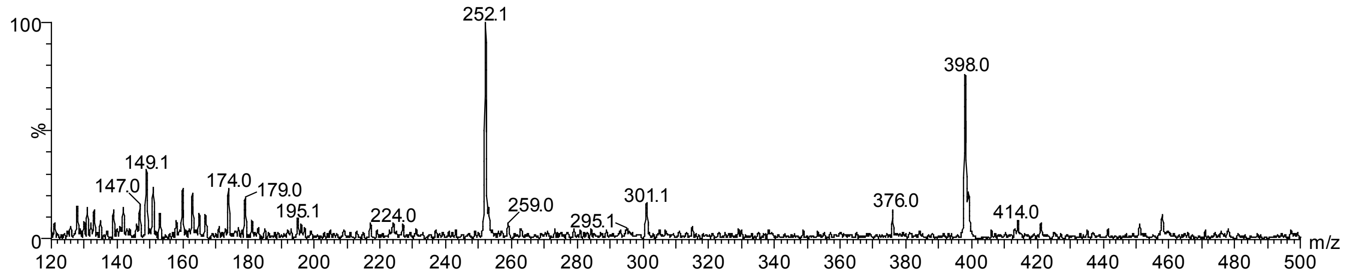

Figure S115. ESI (+) mass spectrum of compound **23.**

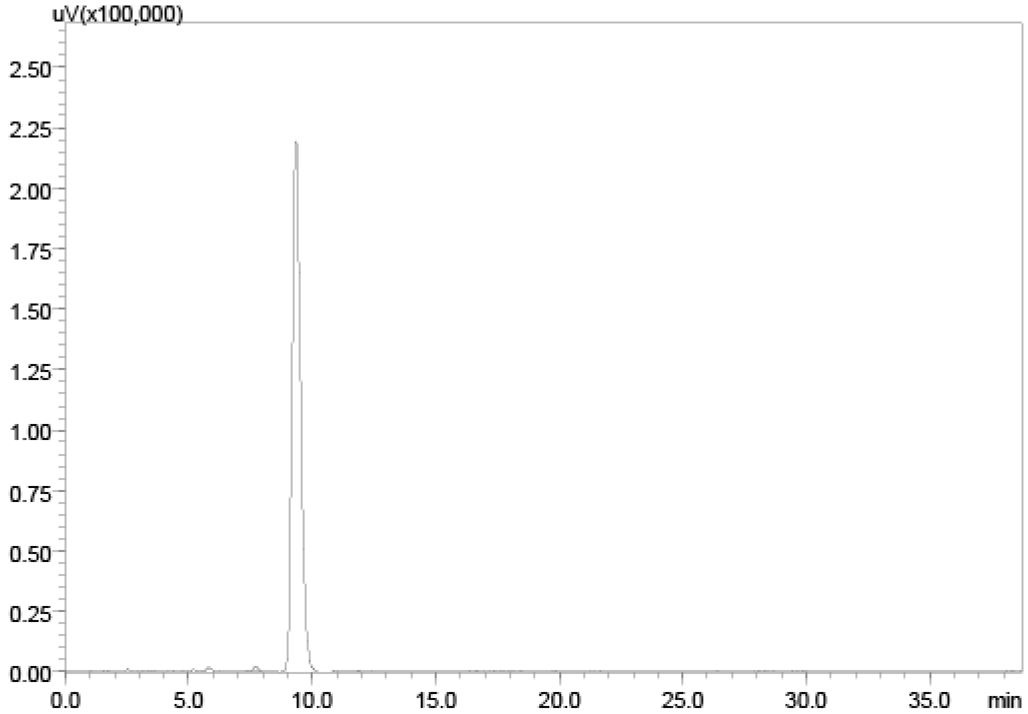

Figure S116: HPLC chromatogram of compound **24**.

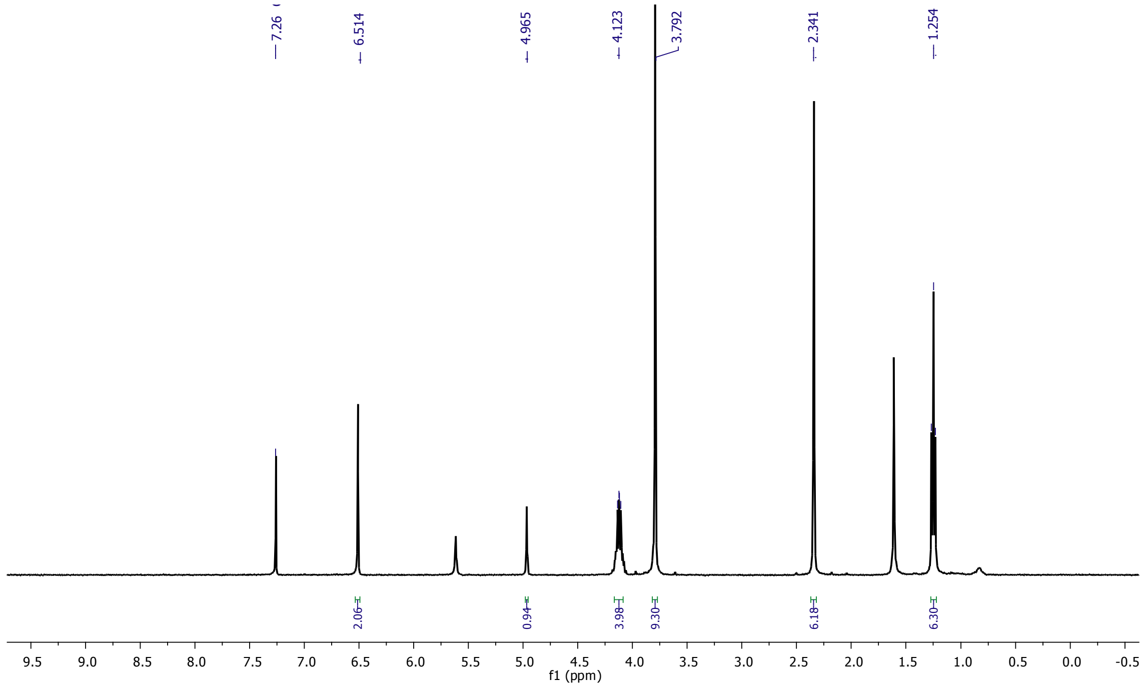

Figure S117.^{1}H NMR spectrum of compound **24** ($CDCl_3$, 400 MHz, TMS).

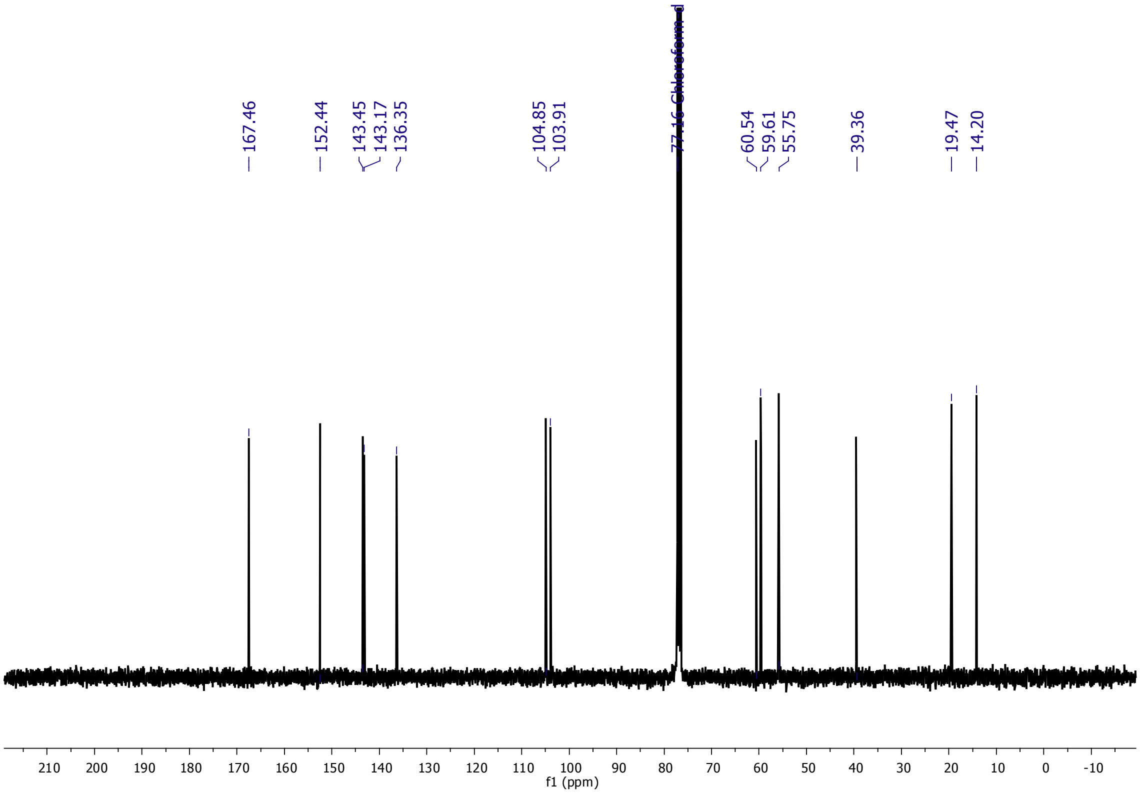

Figure S118.^{13}C NMR spectrum of compound **24** ($CDCl_3$, 100 MHz, TMS).

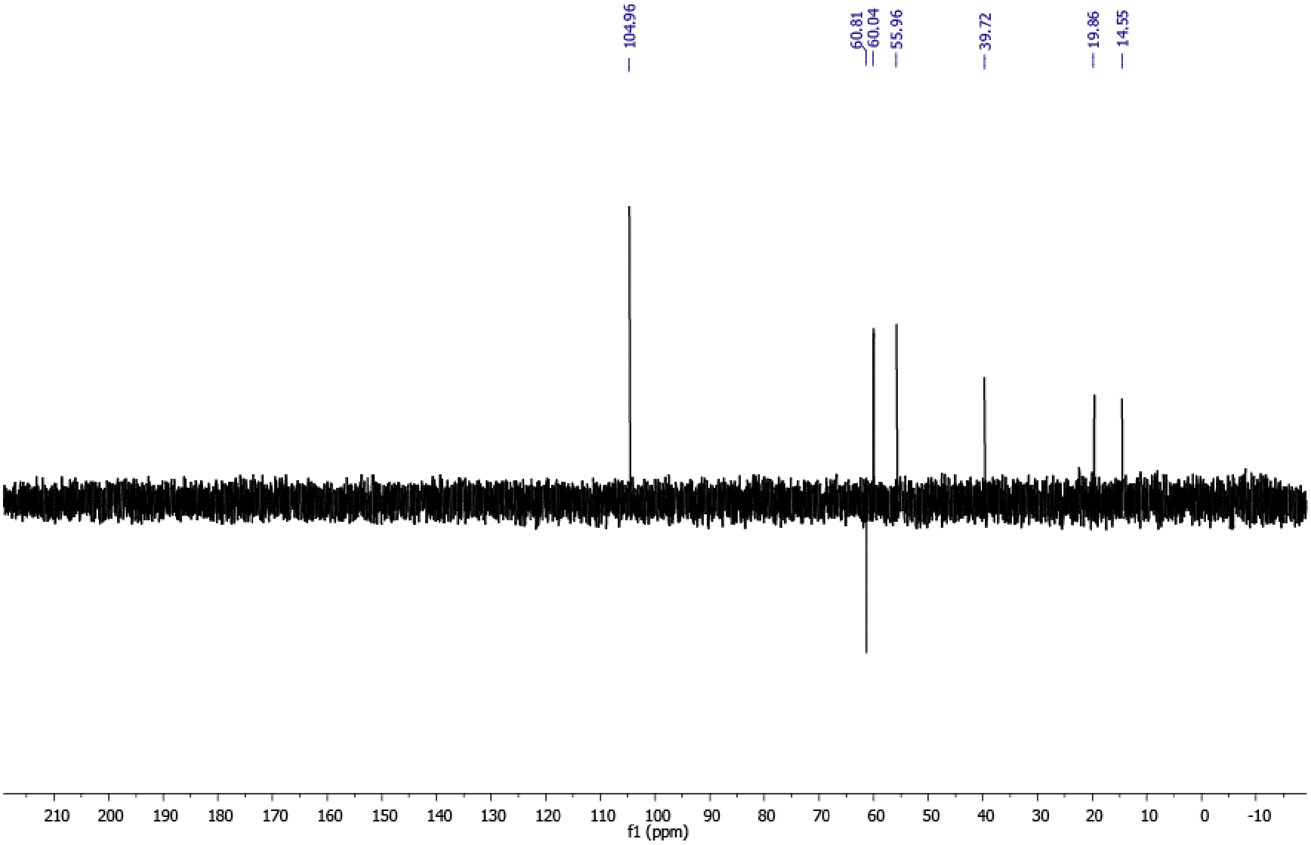

Figure S119. DEPT 135 spectrum of compound **24** ($CDCl_3$, 100 MHz, TMS).

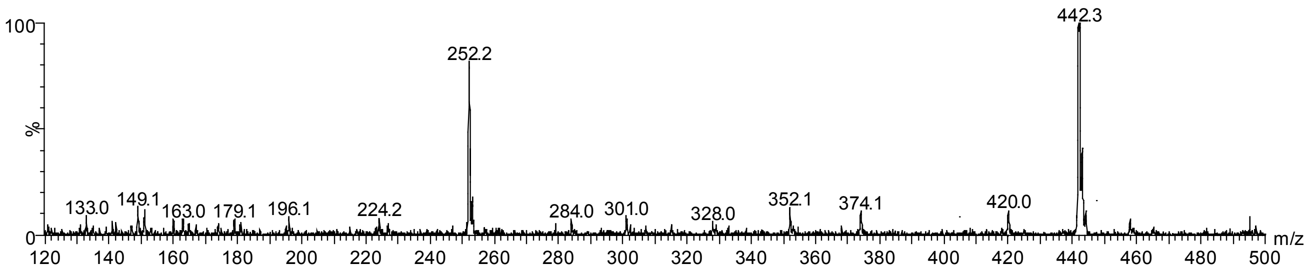

Figure S120. ESI (+) mass spectrum of compound **24.**
